# Robustness encoded across essential and accessory replicons of the ecologically versatile bacterium *Sinorhizobium meliloti*

**DOI:** 10.1371/journal.pgen.1007357

**Published:** 2018-04-19

**Authors:** George C. diCenzo, Alex B. Benedict, Marco Fondi, Graham C. Walker, Turlough M. Finan, Alessio Mengoni, Joel S. Griffitts

**Affiliations:** 1 Department of Biology, University of Florence, Sesto Fiorentino, FI, Italy; 2 Department of Microbiology and Molecular Biology, Brigham Young University, Provo, UT, United States of America; 3 Department of Biology, Massachusetts Institute of Technology, Cambridge, MA, United States of America; 4 Department of Biology, McMaster University, Hamilton, ON, Canada; Universidad de Sevilla, SPAIN

## Abstract

Bacterial genome evolution is characterized by gains, losses, and rearrangements of functional genetic segments. The extent to which large-scale genomic alterations influence genotype-phenotype relationships has not been investigated in a high-throughput manner. In the symbiotic soil bacterium *Sinorhizobium meliloti*, the genome is composed of a chromosome and two large extrachromosomal replicons (pSymA and pSymB, which together constitute 45% of the genome). Massively parallel transposon insertion sequencing (Tn-seq) was employed to evaluate the contributions of chromosomal genes to growth fitness in both the presence and absence of these extrachromosomal replicons. Ten percent of chromosomal genes from diverse functional categories are shown to genetically interact with pSymA and pSymB. These results demonstrate the pervasive robustness provided by the extrachromosomal replicons, which is further supported by constraint-based metabolic modeling. A comprehensive picture of core *S*. *meliloti* metabolism was generated through a Tn-seq-guided *in silico* metabolic network reconstruction, producing a core network encompassing 726 genes. This integrated approach facilitated functional assignments for previously uncharacterized genes, while also revealing that Tn-seq alone missed over a quarter of wild-type metabolism. This work highlights the many functional dependencies and epistatic relationships that may arise between bacterial replicons and across a genome, while also demonstrating how Tn-seq and metabolic modeling can be used together to yield insights not obtainable by either method alone.

## Author summary

*S*. *meliloti*, which has traditionally facilitated ground-breaking insights into symbiotic communication, is also emerging as an excellent model for studying the evolution of functional relationships between bacterial chromosomes and anciently acquired accessory replicons. Multi-replicon genome architecture is present in ~ 10% of presently sequenced bacterial genomes. The *S*. *meliloti* genome is composed of three circular replicons, two of which are dispensable even though they encompass nearly half of the protein-coding genes in this organism. The construction of strains lacking these replicons has enabled a straightforward, genome-wide analysis of interactions between the chromosome and the non-essential replicons, revealing extensive functional cooperation between these genomic components. This analysis enabled a substantial refinement of a metabolic network model for *S*. *meliloti*. The integration of massively parallel genotype-phenotype screening with *in silico* metabolic reconstruction has enhanced our understanding of metabolic network structure as it relates to genome evolution in *S*. *meliloti*, and exemplifies an approach that may be productively applied to other taxa. The combined experimental and computational approach employed here further provides unique insights into the pervasive genetic interactions that may exist within large bacterial genomes.

## Introduction

The prediction of genotype-phenotype relationships is a fundamental goal of genetic, biomedical, and eco-evolutionary research, and this problem underpins the design of synthetic microbial systems for biotechnological applications [1]. Recently, there has been a shift away from the functional characterization of single genes towards whole-genome, systems-level analyses (for recent reviews, see [2,3]). Such studies have been facilitated by methods allowing for the direct interrogation of a genome to determine all genetic elements required for adaptation to a given environment. Two primary methods are *in silico* metabolic modeling [4,5], and massively parallel sequencing of transposon insertions in bacterial mutant libraries (Tn-seq) [6,7].

*In silico* genome-scale metabolic modeling attempts to reconstruct all cellular metabolism, including all biochemical reactions and the genes encoding the participating enzymes, thereby linking genetics to metabolism [8]. Next, mathematical models such as flux balance analysis (FBA) are used to simulate the flux distribution through the reconstructed network [9], allowing predictions of how environmental perturbations or gene disruptions would influence growth. This approach allows for phenotypic predictions of all single, double, or higher-order gene deletion mutations within a matter of days [10,11], which is infeasible using a direct experimental approach. However, the quality of the predictions is highly dependent on the accuracy of the metabolic reconstruction. Outside of a few model species like *Escherichia coli*, experimental genetic and biochemical data are not available at the resolution necessary to provide accurate assignment of all metabolic gene functions.

Tn-seq involves the generation of a library of hundreds of thousands of mutant clones, each containing a single transposon insertion at a random genomic location (refer to [12] for a review on this method). The library of pooled clones is then cultured in the presence of a defined environmental challenge. Insertions resulting in altered fitness in the environment under investigation become under- or over-represented in the population. Deep sequencing is used to identify the genomic location and frequency of all transposon insertions, which is then used as a measure of the growth phenotype brought about by specific mutations: a less than expected number of insertions within a gene is interpreted to reflect that mutation of the gene impairs growth. This approach is imperfect, as important biochemical functions may be redundantly encoded [13–15], or replaced by compensatory alternative processes [16]. Moreover, fitness changes brought about by mutation in one gene may require mutation of a second gene bearing no resemblance to the first—a phenomenon known as a genetic interaction [17,18]. Such genetic interactions may cause the apparent functions of some genes to be strictly dependent on their genomic environment [19]. In other words, a gene may be essential for growth in one organism, but its orthologous counterpart in another organism may be non-essential. This significantly complicates efforts to generalize genotype-phenotype relationships [20].

Resolving the problem of genome-conditioned gene function is of broad significance in the areas of functional genomics, population genetics, and synthetic biology. For example, the ability to design and build optimized minimal cell factories on the basis of single-mutant fitness data is expected to present numerous complications [21], as evidenced by the recent effort to rationally build a functional minimal genome [22]. Tn-seq studies suggest there is as little as 50% to 25% overlap in the essential genome of any two species [23–25]. As a striking example, 210 of the Tn-seq essential genes of *Pseudomonas aeruginosa* PA14 are not even present in the *P*. *aeruginosa* PAO1 genome [26]. Comparison of Tn-seq data for *Shigella flexneri* with deletion analysis data for the closely related species *E*. *coli* suggested only a small number of genes were specifically essential in one species; however, mutation of about 100 genes appeared to result in a growth rate decrease specifically in *E*. *coli* [27]. Similarly, comparison of Tn-seq datasets from two *Salmonella* species revealed that mutation of nearly 40 genes had a stronger growth phenotype in one of the two species [28]. Overall, these studies suggest that the genomic environment (here defined as the genomic components that may vary from organism to organism) influences the fitness contributions of a significant proportion of an organism’s genes. However, no large-scale analysis has been performed to directly examine how the phenotypes of individual genes are influenced after large-scale genomic manipulation.

Here, we provide a quantitative, genome-scale evaluation of how large-scale genomic variance influences genotype-phenotype relationships. The model system used is *Sinorhizobium meliloti*, an α-proteobacterium whose 6.7-Mb genome consists of a chromosome and two additional replicons, the pSymA megaplasmid and the pSymB chromid [29,30]. The pSymA and pSymB replicons constitute 45% of the *S*. *meliloti* genome (~2,900 genes); yet, by transferring only two essential genes from pSymB to the chromosome, both pSymA and pSymB can be completely removed from the genome, yielding a viable single-replicon organism [31]. We report a comparison of gene essentiality (via Tn-seq) for wild-type *S*. *meliloti* and the single-replicon derivative. This experiment was designed to evaluate interactions between individual chromosomal genes and the secondary genome (pSymA/pSymB) as a whole, recognizing that the pSymA/pSymB deletion, while consistent with cell viability, is certainly pleiotropic [32,33]. This enables us to detect the chromosomal side of inter-replicon genetic interactions with single-gene precision, but unable to discern the extrachromosomal side of the interactions. This high-throughput genetic analysis is supplemented by an *in silico* double gene deletion analysis of a *S*. *meliloti* genome-scale metabolic network reconstruction. We further examine how integration of Tn-seq data with *in silico* metabolic modeling, through a Tn-seq-guided reconstruction process, overcomes the limitations of using either of these approaches in isolation to develop a consolidated view of the core metabolism of the organism. This process produced a fully referenced core *S*. *meliloti* metabolic reconstruction.

## Results

### Development and validation of the Tn*5*-based transposon Tn*5*-714

**Fig 1:**
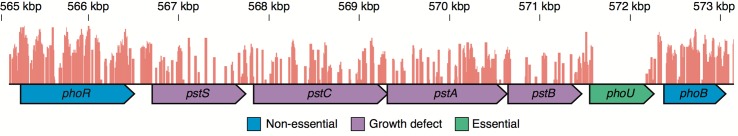
Visualization of the location of transposon insertion sites. An image of the *pst* locus of *S*. *meliloti* generated using the Integrative Genomics Viewer [78]. Chromosomal nucleotide positions are indicated along the top of the image, and the location and relative abundance of transposon insertions are indicated by the red bars. Non-essential genes contain a high density of transposon insertions, whereas essential genes have few to no transposon insertions. Genes are color coded based on their fitness classification. The *pstS*, *pstC*, *pstA*, *pstB*, *phoU*, and *phoB* genes are co-transcribed as a single operon [92], and previous work demonstrated that non-polar *phoU* mutations are lethal in *S*. *meliloti*, whereas polar mutations are not lethal [93]. The lack of insertions within the *phoU* coding region is therefore consistent with the non-polar nature of the transposon.

**Fig 2:**
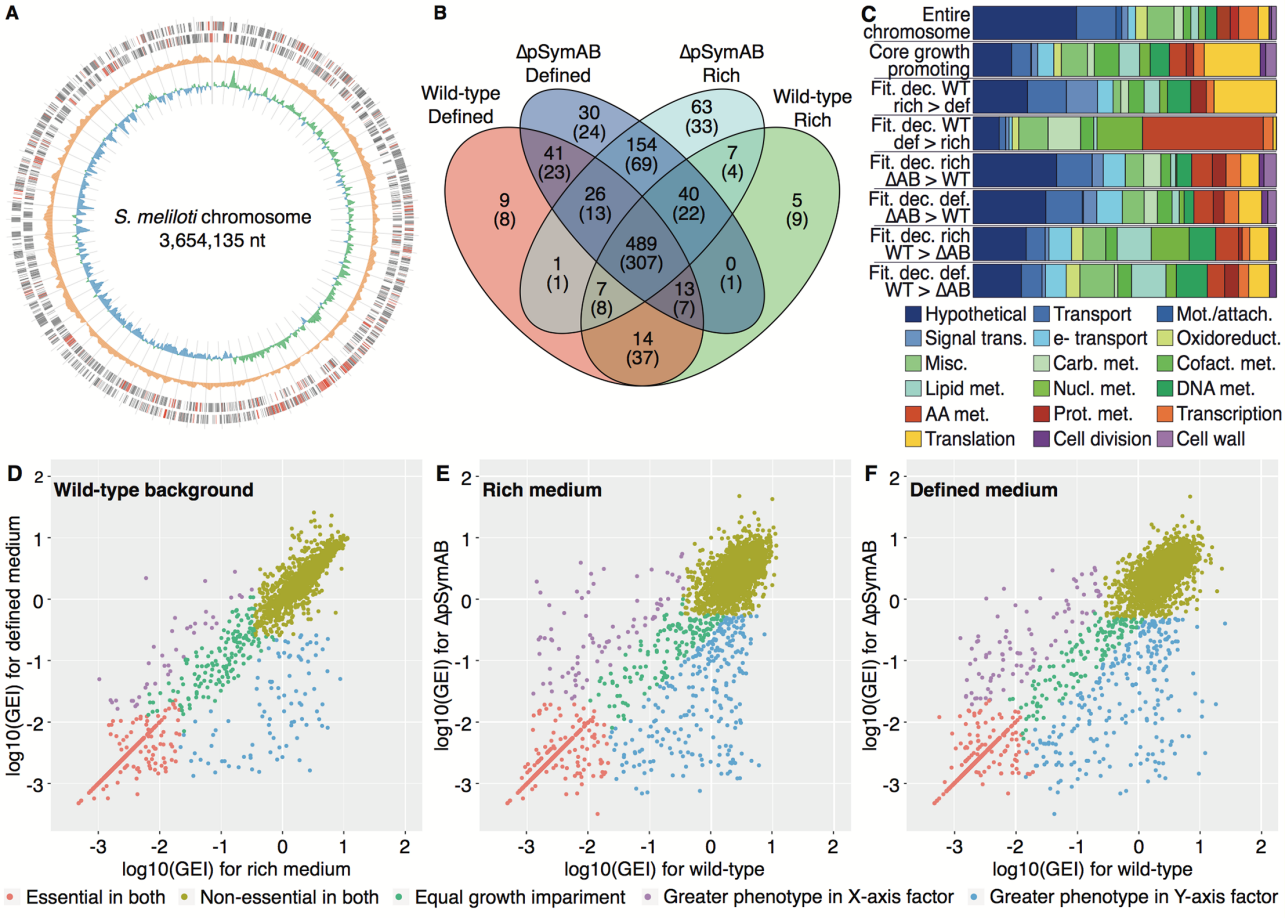
Characteristics of the core genetic components of *S*. *meliloti*. (**A**) A circular plot of the *S*. *meliloti* chromosome is shown. From the outside to inside: positive strand coding regions, negative strand coding regions (for both positive and negative strands, red indicates the position of the core 489 growth promoting genes), total insertion density, and GC skew. The insertion density displays the total transposon insertions across all experiments over a 10,000-bp window. The GC skew was calculated over a 10,000-bp window, with green showing a positive skew and blue showing a negative skew. Tick marks are every 50,000 bp. (**B**) A comparison of the overlap between the growth promoting genome (Group I and II genes, shown first) and the essential genome (Group I genes, values in parentheses) of each Tn-seq dataset. Each dataset is labeled with the strain (wild-type or ΔpSymAB) and the growth medium (defined medium or rich medium). (**C**) Functional enrichment plots for the indicated gene sets. Name abbreviations: Fit–fitness; Dec–decrease; WT–wild-type; ΔAB—ΔpSymAB; Def–defined medium; Rich–rich medium. For example, ‘Fit. dec. WT def > rich' means the genes with a greater fitness decrease in wild-type grown in defined medium compared to rich medium. Legend abbreviations: AA–amino acid; Attach–attachment; Carb–carbohydrate; Cofact–cofactor; e-–electron; Met–metabolism; Misc–miscellaneous; Mot–motility; Nucl–nucleotide; Oxidoreduct–oxidoreductase activity; Prot–protein; Trans–transduction. (**D**-**F**) Scatter plots comparing the fitness phenotypes, shown as the log_10_ of the GEI scores (Gene Essentiality Index scores; i.e., number of insertions within the gene divided by gene length in nucleotides) of (**D**) wild-type grown in rich medium versus wild-type grown in defined medium, (**E**) wild-type grown in rich medium versus ΔpSymAB grown in rich medium, and (**F**) wild-type grown in defined medium versus ΔpSymAB grown in defined medium.

To interrogate the *S*. *meliloti* genome using a Tn-seq approach, we first developed a new construct based on the Tn*5* transposon as described in the Materials and Methods. The resulting transposon (S1 Fig) contains constitutive promoters reading out from both ends of the transposon to ensure the production of non-polar mutations; without such promoters, non-essential genes at the beginning of an operon may be incorrectly classified as essential if a downstream operon gene is essential. However, the inclusion of outward facing promoters means that surrounding genes are constitutively expressed, potentially resulting in growth impairments, and this must be kept in mind during examination of the data. Analysis of the insertion site locations validated that the transposon performed largely as expected. Gene disruptions caused by transposon insertions were confirmed to be non-polar as illustrated by the case reported in Fig 1, and there was no strong bias in the distribution of insertions around the chromosome (Fig 2A, S2 Fig). However, there did appear to be somewhat of a bias for integration of the transposon in GC-rich regions (S3 Fig). As there was little relationship between GC content and Tn-seq derived gene essentiality scores (S4 Fig), it is unlikely that this moderate bias had a discernable influence on the results of this study.

### Overview of the Tn-seq output

Our Tn-seq experiments were undertaken with two primary aims: i) to identify the core set of genes contributing to *S*. *meliloti* growth under laboratory conditions, and ii) to determine the extent to which the phenotypic consequence of a gene deletion is influenced by the genomic environment (i.e. presence/absence of the secondary replicons). To accomplish this, Tn-seq libraries were prepared for two isogenic *S*. *meliloti* strains: RmP3496 (ΔpSymAB), which lacks the pSymA and pSymB replicons; and RmP3499 (wild type), resulting from the restoration of pSymA and pSymB into RmP3496 [34]). Strain RmP3499 was used as the wild-type as whole-genome sequencing revealed only three chromosomal polymorphisms between RmP3496 and RmP3499; in contrast, there are 23 polymorphisms between the chromosomes of RmP3496 and the wild-type Rm2011 [34].

Transposon library sizes were skewed to compensate for the difference in genome sizes (~ 1.8-fold more insertion mutants were collected for the wild type), resulting in nearly identical insertion site density for each library (S1 Table). Both libraries were passed in duplicate through selective growth regimens in either complex BRM broth (rich medium containing yeast extract, tryptone, and sucrose) or minimal defined broth (a medium containing a minimal set of salts required for growth, including sucrose, ammonium, sulfate, and phosphate). These media were chosen as both were found to support growth of both strains, and they represent two very different nutritional environments. Following approximately nine generations of growth, the locations of the transposon insertions in the population were determined, a gene essentiality index (GEI) was calculated for all chromosomal genes, and each gene was classified into one of five fitness categories (Table 1) using the procedure described in the Materials and Methods. Four genes that were found to be essential in the Tn-seq data (*pdxJ*, *fumC*, *smc01011*, *smc03995*) were independently tested in the wild-type background by targeted knock-out. In all cases, the mutations yielded the expected no-growth phenotype (S5 Fig), supporting the accuracy of the Tn-seq output. All Tn-seq data are available as S1 Dataset. Data for the pSymA and pSymB replicons in the wild-type strain are also provided in S1 Dataset, but these data will not be considered further in this manuscript.

**Table 1:** Fitness classification of chromosomal genes. Genes were ranked from lowest to highest GEI, with the lowest GEI being at the 0 percentile and the highest GEI being at the 100^th^ percentile. The approximate break points for the groupings, determined as described in the Materials and Methods, are shown for each condition. The ranges did not play a role in the classification of the genes, but serve to summarize the number of genes in each category.

		GEI percentile range			
Group	Description	Wild-type, rich medium	ΔpSymAB, rich medium	Wild-type, defined medium	ΔpSymAB, defined medium
I	Essential	0–12	0–14	0–12	0–14
II	Strong growth defect	12–17	14–23	12–18	14–24
III	Moderate growth defect	17–36	23–49	18–28	24–47
IV	Little to no growth impact	36–100	49–96	28–99	47–99
V	Growth improvement	N/A	96–100	99–100	99–100

A strong correlation was observed between the number of insertions per gene in each set of duplicates (S6 Fig), indicating that there was high reproducibility of the results and that differences between conditions were unlikely to reflect random fluctuations in the output. On average, insertions were found in 190,000 unique chromosomal positions with a median of 39 unique insertion positions per gene (S1 Table). The similarity in the number of unique insertion positions between samples confirmed that equal coverage of the two strains was obtained, and suggested that differences in the Tn-seq outputs were unlikely to be an artifact of the quality of the libraries.

### Elucidation of the core genetic components of *S*. *meliloti*

The growth phenotype of mutating each of the *S*. *meliloti* chromosomal genes was inferred based on the density of transposon insertions within the gene, with fewer insertions suggesting a larger growth impairment when the gene is mutated. Based on this approach, 307 genes were classified as essential independent of growth medium or strain (Fig 2B). This set of 307 genes includes those encoding functions commonly understood to be essential: the DNA replication apparatus, the four RNA polymerase subunits, the housekeeping sigma factor, the general transcriptional termination factor Rho, 40 out of 55 of the annotated ribosomal protein subunits, 18 out of 20 of the annotated aminoacyl-tRNA synthetases, and 6 out of 10 of the annotated ATP synthase subunits. Considering genes classified as essential plus those genes whose mutation resulted in a large growth defect (Groups I and II in Table 1), a core growth promoting genome of 489 genes, representing ~ 15% of the chromosome, was identified (Fig 2B). This expanded list includes 51 out of 55 of the annotated ribosomal protein subunits, 19 out of 20 of the annotated aminoacyl-tRNA synthetases, and 9 out of 10 of the annotated ATP synthase subunits. These 489 genes appeared to be mostly dispersed around the chromosome, although there was a bias for these genes to be found in the leading strand (Fig 2A), and many ribosomal and RNA polymerase genes are grouped together in one locus (the 5 o’clock position in Fig 2A). Based on published RNA-sequencing data for *S*. *meliloti* grown in a glucose minimal medium, these 489 genes tend to be highly expressed, with a median expression level above the 90^th^ percentile (S7 Fig). Compared to the entire chromosome (Fisher exact test, p-value < 0.05 following a Bonferroni correction for 18 tests), this set of 489 genes was enriched for genes involved in translation (5.2-fold), lipid metabolism (2.7-fold), cofactor metabolism (3.3-fold), and electron transport (2.1-fold), whereas genes involved in transport (2.1-fold), motility/attachment (9.4-fold), and hypothetical genes (2.7-fold) were under-represented (Fig 2C).

A clear influence of the growth medium on the fitness phenotypes of gene mutations was observed. Focusing on the wild-type strain, a core of 519 genes were identified as contributing equally to growth in both media (Fig 2D). Forty genes were identified as more important during growth in rich medium than in defined medium, and these genes had a median GEI fold change of 7. Only translation functions (5.8-fold) displayed a statistically significant enrichment in these genes, which may reflect the faster growth rate in the rich medium (S8 Fig), while there was also a non-statistically significant enrichment in signal transduction (5.1-fold) (Fig 2C). The extent of specialization for growth in the defined medium was more pronounced; 93 genes were more important during growth in the defined medium with a median GEI fold change of 20. These genes were enriched (statistically significant) in amino acid (9.0-fold) and nucleotide (6.7-fold) metabolism presumably due to the requirement of their biosynthesis, and carbohydrate metabolism (3.6-fold) likely as the sole carbon source was a carbohydrate (Fig 2C). The same overall patterns were observed between media for the ΔpSymAB strain (S9 Fig).

### Mutant fitness phenotypes are strongly influenced by their genomic environment

The Tn-seq datasets for the wild-type and the ΔpSymAB strains were compared to evaluate fitness phenotypes across the two genetic backgrounds. Similar global trends were observed for both growth media (rich and defined), suggesting that the results were generalizable and not medium-specific. Mutation of 488 (rich) or 484 (defined) chromosomal genes decreased growth in both genetic backgrounds. However, we also observed striking strain-dependent phenotypic effects. In ΔpSymAB cells, mutations of 250 (rich) or 251 (defined) genes led to stronger growth impairments than in wild-type cells; and conversely, in wild-type cells, mutations of 81 (rich) or 89 (defined) genes led to stronger growth impairments than in ΔpSymAB cells (Fig 2E and 2F and Table 2). Only minor functional biases were observed in the genes associated with larger fitness defects in the ΔpSymAB background (Fig 2C); in both media, only electron transport (3-fold) and oxidoreductases (9.5-fold) were over- and under-represented, respectively. Similarly, few functional biases were detected in genes associated with larger fitness defects in the wild-type background (Fig 2C); in both media, lipid metabolism (4.5-fold) and hypothetical genes (2-fold) were over- and under-represented, respectively, while nucleotide metabolism (5.5-fold) was also enriched in the rich medium data. Overall, these results are consistent with pervasive functional connections between the chromosome and the secondary replicons, showing no strong bias toward specific biochemical pathways.

**Table 2:** Sample genes showing strain specific phenotypes. The top ten genes from each of the indicated groupings, as determined based on the ratio of GEI (Gene Essentiality Index) scores of the two strains, are shown. GEI scores are shown first for the wild-type (WT) followed by the scores for the ΔpSymAB (dAB) strain.

Gene	Function	GEI (WT … dAB)	Gene	Function	GEI (WT … dAB)
More Important ΔpSymAB—Rich Medium			More Important ΔpSymAB—Defined Medium		
*kpsF3*	capsule expression protein	5.951 … 0.002	*smc03782*	signal peptide protein	9.670 … 0.001
*groEL*	chaperonin GroEL	2.926 … 0.001	*amiC*	N-acetylmuramoyl-L-alanine amidase	12.273 … 0.003
*aidB*	oxidoreductase	2.658 … 0.001	*groEL*	chaperonin GroEL	2.755 … 0.001
*proA*	γ-glutamyl phosphate reductase	4.505 … 0.001	*amn*	AMP nucleosidase	2.200 … 0.001
*amiC*	N-acetylmuramoyl-L-alanine amidase	2.016 … 0.002	*ndvA*	cyclic beta-1,2-glucan ABc transporter	1.434 … 0.001
*smc03782*	signal peptide protein	1.847 … 0.001	*smc02495*	translaldolase	3.933 … 0.003
*etfA1*	electron transfer flavoprotein	2.447 … 0.002	*glnA*	glutamine synthetase	2.535 … 0.002
*smc02495*	translaldolase	3.127 … 0.003	*glmS*	glucosamine—fructose-6P aminotransferase	1.918 … 0.002
*exoN2*	UTP—glucose-1P uridylyltransferase	2.292 … 0.002	*smc00717*	ABC transporter ATP-binding protein	6.624 … 0.006
*glnA*	glutamine synthetase	2.153 … 0.002	*etfA1*	electron transfer flavoprotein	2.156 … 0.002
More Important Wild-type—Rich Medium			More Important Wild-type—Defined Medium		
*carB*	carbamoyl phosphate synthase	0.001 … 1.930	*folD2*	5,10-methylene-THF dehydrogenase	0.003 … 1.026
*argG*	argininosuccinate synthase	0.002 … 1.281	*nuoK1*	NADH dehydrogenase subunit K	0.006 … 1.476
*carA*	carbamoyl phosphate synthase	0.007 … 3.928	*prfC*	peptide chain release factor RF-3 protein	0.001 … 0.232
*purB*	adenylosuccinate lyase	0.002 … 0.799	*smc00714*	1-acyl-SN-glycerol-3P acyltransferase	0.005 … 0.522
*hrm*	histone-like protein	0.011 … 2.586	*fpr*	ferredoxin—NADP reductase	0.002 … 0.248
*nuoK1*	NADH dehydrogenase subunit K	0.006 … 1.330	*smc00532*	hypothetical protein	0.002 … 0.203
*folD2*	5,10-methylene-THF dehydrogenase	0.018 … 3.110	*ubiE*	ubiquinone biosynthesis methyltransferase	0.002 … 0.209
*coaA*	pantothenate kinase	0.004 … 0.595	*asd*	aspartate-semialdehyde dehydrogenase	0.002 … 0.157
*argF1*	ornithine carbamoyltransferase	0.012 … 1.776	*secE*	preprotein translocase subunit SecE	0.010 … 0.796
*smc00914*	oxidoreductase	0.002 … 0.240	*smc01038*	hypothetical protein	0.040 … 2.940

Of 16 arbitrarily chosen genes predicted to be important for growth specifically in one of the two strains, nine of them, when disrupted in a targeted manner, led to the expected strain-specific strong growth inhibition (S10 Fig). Of the other seven genes, three (*cbrA*, *ppk*, *tig*) were non-lethal but displayed growth rate defects or extended lag phases during liquid culture experiments (S2 Table and S11 Fig). The remaining four genes may represent false positives from the Tn-seq screen; alternatively, the contrasting results may reflect differences in the growth conditions of the Tn-seq experiment (competitive growth in a genetically complex population) compared to the validation experiment (monoculture). The results for the *feuQ* gene may be representative of how the failure to validate a result may be due to difference in experimental set-ups and not due to a false positive in the Tn-seq data (see the Supplementary Results in S1 File). Regardless, the observation that at least 75% of the selected genes were confirmed to have a genome content-dependent fitness phenotype indicate that the large majority of the strain specific phenotypes observed in the Tn-seq screen represent true differences.

### Level of genetic and phenotypic conservation of the essential *S*. *meliloti* genes

**Fig 3:**
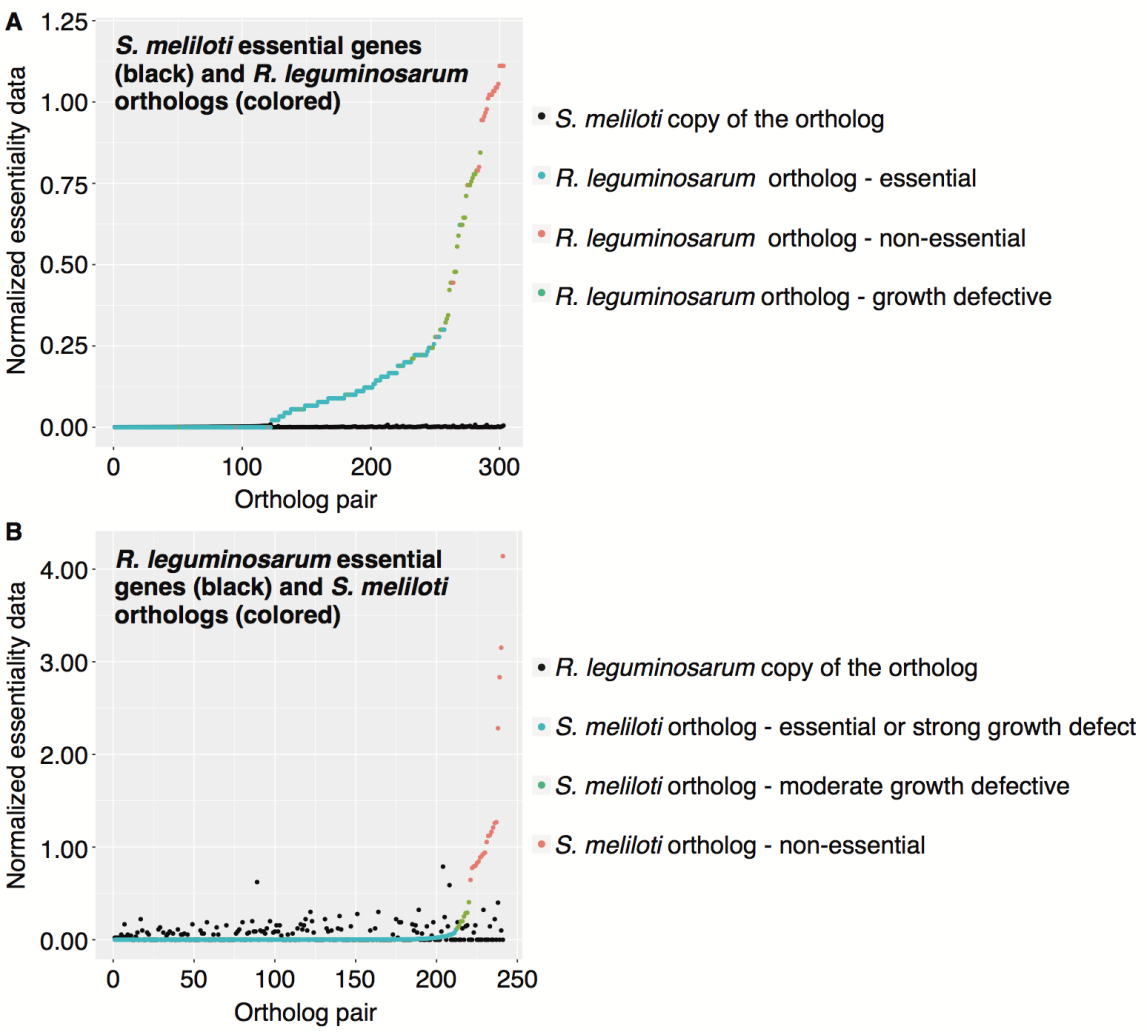
Comparison of *S*. *meliloti* and *R*. *leguminosarum* Tn-seq data. (**A**) The fitness phenotypes of essential *S*. *meliloti* genes, as determined in this study, are compared to the fitness phenotypes of the orthologous *R*. *leguminosarum* genes, as determined by Perry *et al*. [36]. The data for the *S*. *meliloti* copy of the genes are shown in black, while the data for the *R*. *leguminosarum* copy of the genes are colored according to their classification by Perry *et al*. [36]. (**B**) The fitness phenotypes of essential *R*. *leguminosarum* genes is compared to the fitness phenotypes of the orthologous *S*. *meliloti* genes. The data for the *R*. *leguminosarum* copy of the genes are shown in black, while the data for the *S*. *meliloti* copy of the genes are colored according to their classification in this study. (**A**,**B**) Normalized fitness values are used to facilitate direct comparison between the studies as different output statistics were calculated. For *S*. *meliloti*, the GEI score of each gene for wild-type cells grown in minimal medium broth was divided by the median GEI for all genes under the same conditions. For *R*. *leguminosarum*, the insertion density of each gene during growth on minimal medium plates was divided by the median insertion density of all genes. The data underlying this figure and the corresponding gene names are provided in S3 Dataset.

Several recent studies have used Tn-seq to study the essential genome of *Rhizobium leguminosarum* [35–37]. We compared our Tn-seq datasets with those reported by Perry *et al*. [36] to examine the conservation of the essential genome of these two closely related legume symbionts. Putative orthologs for ~ 75% of all *S*. *meliloti* chromosomal genes were identified in *R*. *leguminosarum* via a Blast Bidirectional Best Hit (Blast-BBH) approach (S2 Dataset). Much higher conservation of the growth promoting genome was observed; 97% of the 489 core growth promoting genes and 99% of the 307 core essential genes had a putative ortholog in *R*. *leguminosarum*. However, conservation of the gene did not necessarily correspond to conservation of the phenotype. Considering only the 303 core essential *S*. *meliloti* genes with a putative ortholog in *R*. *leguminosarum*, 8% (25 of 303) of the *R*. *leguminosarum* orthologs were classified as having little contribution to growth on defined medium (Fig 3A). Two genes (*fumC*, *pdxJ*) identified as essential in *S*. *meliloti* but non-essential in *R*. *leguminosarum* based on the Tn-seq data were mutated by targeted disruption in *S*. *meliloti*. In both cases, the genes were confirmed to be essential in *S*. *meliloti* (S5 Fig), supporting the Tn-seq data. A similar pattern is observed when starting with the *R*. *leguminosarum* genes classified as essential in both minimal and complex medium by Perry *et al*. [36]. Of the 241 core essential *R*. *leguminosarum* genes with an ortholog on the *S*. *meliloti* chromosome, 21 (9%) of the orthologs were classified as having no contribution to growth in defined medium in *S*. *meliloti* (Fig 3B). Whether these species-specific essentiality phenotypes are unique to the tested environment, or if they would also be observed in a more ecologically relevant environment, remains unclear.

To further test the species-specificity of the above-mentioned genes, the mutational analysis was replicated *in silico*, based on metabolic reconstructions. Fifteen of the 25 orthologs specifically essential in *S*. *meliloti* were present in our existing *S*. *meliloti* genome-scale metabolic model [38] as well as in a draft *R*. *leguminosarum* metabolic model (see Materials and Methods). Flux balance analysis was used to examine the *in silico* growth effect of deleting these 15 pairs of orthologs. Three pairs were classified as essential in both models, five were classified as non-essential in both models, and seven were classified as essential specifically in the *S*. *meliloti* model (S3 Dataset). The analysis was repeated using a more rudimentary draft *S*. *meliloti* model to see if the results were strongly influenced by the curation level of the models. In this case, three pairs of orthologs were classified as essential in both models, seven were classified as non-essential in both models, and five were classified as essential specifically in the *S*. *meliloti* model (S3 Dataset). Thus, even with the draft metabolic models, the *in silico* metabolic simulations corroborate at least some of the inter-species gene essentiality differences observed in the Tn-seq data.

### *In silico* analyses support a high potential for genetic redundancy in the *S*. *meliloti* genome

The results of the previous two sections are consistent with a strong genomic environment effect on the phenotypic consequences of gene mutations. One possible explanation is the presence of widespread genetic redundancy, at the gene and/or pathway level. In support of this, ~ 14% of chromosomal genes had a significant Blast hit when the chromosomal and pSymA/pSymB proteomes was compared against each other (S4 Dataset). Therefore, this phenomenon was further explored using a constraint-based metabolic modeling approach.

**Fig 4:**
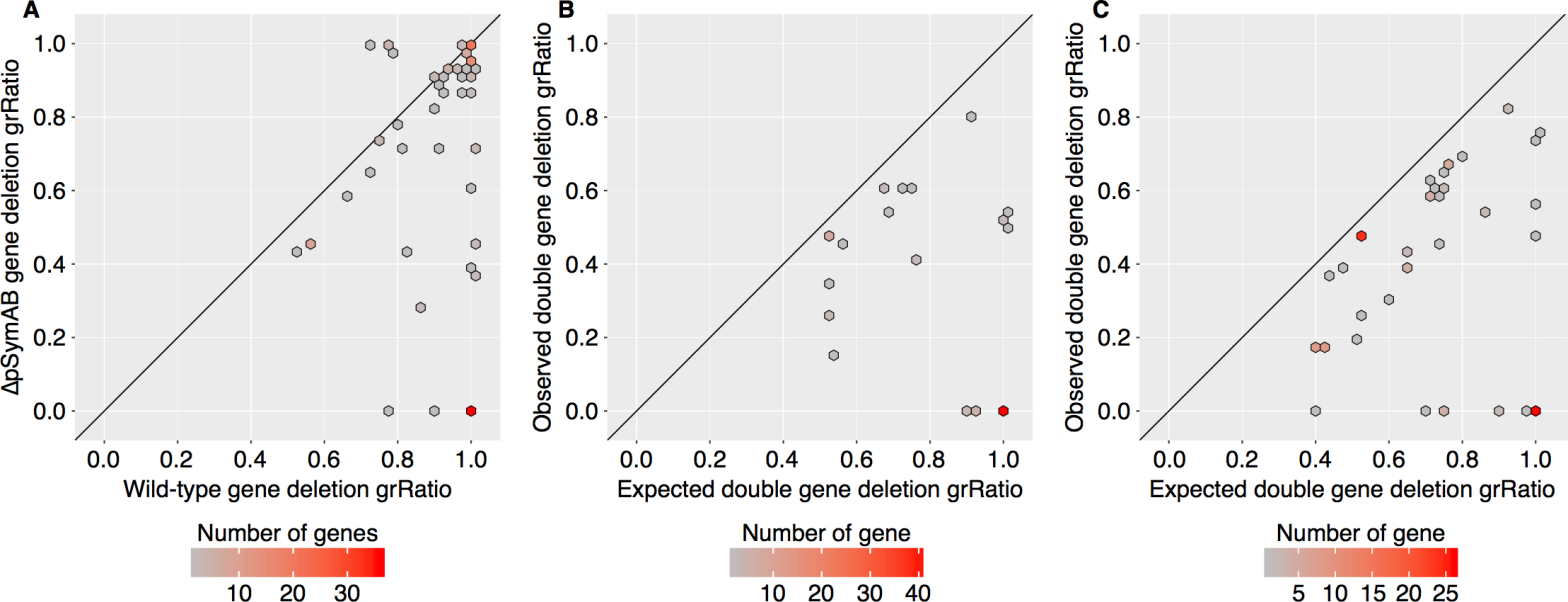
*In silico* analysis of genetic redundancy in *S*. *meliloti*. The effects of single or double gene deletion mutants were predicted *in silico* with the genome-scale *S*. *meliloti* metabolic model. All data in this figure comes from *in silico* metabolic modeling. (**A**-**C**) The color of each hexagon is representative of the number of genes, gene pairs, or reaction pairs plotted at that location according to the density bar below each panel. The diagonal line serves as a reference line of a perfect correlation (i.e., where all data would fall if no effects were observed). The data underlying this figure and the corresponding gene/reaction names are provided in S7 Dataset. (**A**) A scatter plot comparing the grRatio (growth rate of mutant / growth rate of non-mutant) for gene deletion mutations in the presence (wild-type) versus absence (ΔpSymAB) of the pSymA/pSymB model genes. Genes whose deletion had either no effect or were lethal in both cases are not included in the plot. (**B**) A scatter plot comparing the grRatio for each double gene deletion pair (where one gene was on the chromosome and the other on pSymA or pSymB) observed *in silico* versus the predicted grRatio based on the grRatio of the single deletions (grRatio1 * grRatio2). Only gene pairs with an observed grRatio at least 10% less than the expected are shown. (**C**) A scatter plot comparing the grRatio for each double gene deletion pair (both genes on the chromosome) observed *in silico* versus the predicted grRatio. Only gene pairs with an observed grRatio at least 10% less than the expected are shown.

We first tested the *in silico* effect of chromosomal single gene deletions on growth rate in the presence and absence of pSymA/pSymB (Fig 4A). This analysis identified 67 genes (~ 7% of all chromosomal model genes) as having a more severely impaired growth phenotype when deleted in the absence of pSymA/pSymB genes, 38 of which were lethal. This appeared to be due to a combination of direct functional redundancy of the gene products as well as through metabolic bypasses, as deletion of 50 reactions dependent on chromosomal genes had a more severe phenotype in the absence of pSymA/pSymB, 42 of which were lethal (S12 Fig). However, there was little overlap between the *in silico* and Tn-seq data (S13 Fig); in cases of differences, we would consider the Tn-seq data to be more accurate. Differences between datasets may be a result of errors in the model, or could reflect that the model does not account for regulation of gene expression; i.e., two genes may be correctly predicted as functionally redundant, but this is not observed experimentally as they are expressed in unique conditions.

Next, a double gene deletion analysis was performed to examine the effect on growth rate of deleting every possible pair of genes in the model. This analysis suggested that 75 chromosome-pSymAB gene pairs (encompassing 49 chromosomal genes) had a more significant impact on growth than expected based on the combination of predicted single-mutant phenotypes (Fig 4B). Additionally, synthetic negative phenotypes were observed when simultaneously deleting 111 chromosome-chromosome gene pairs (totaling 97 chromosomal genes) (Fig 4C). Overall, 14% of chromosomal genes were predicted to have a synthetic negative phenotype when co-deleted with a second gene. Even if metabolic modeling over-predicts the presence of functional redundancy in a specific environment (for example, due to regulatory differences), these results are consistent with a high potential for metabolic robustness being encoded both within and between replicons in the *S*. *meliloti* genome.

### A consolidated view of core *S*. *meliloti* metabolism through Tn-seq-guided *in silico* metabolic reconstruction

The results described in the previous sections made it evident that a Tn-seq approach alone is insufficient to elucidate all processes contributing to growth in a particular environment. This is especially true if also considering non-essential metabolism that is nevertheless actively present in wild-type cells, such as exopolysaccharide production. Moreover, it is difficult to fully comprehend the core functions of a cell by simply examining a list of essential genes and their predicted functions. We therefore attempted to overcome these limitations by using the Tn-seq data to guide a manual *in silico* reconstruction of the core metabolic processes of *S*. *meliloti*. A detailed description of this process is provided in the Supplementary Methods of S1 File. In brief, we began with a reconstruction that consisted of only one reaction, the biomass production reaction that combined all biomass components (e.g., protein, DNA, RNA, lipids, cofactors, etc.) into ‘biomass’. Initially, only protein was included as a biomass component. Pathways required to produce protein were built reaction by reaction in this new reconstruction, drawing from the reaction pool present in the existing genome-scale metabolic reconstruction, where possible. At the same time, the genes associated with each reaction were compared to the Tn-seq data and published literature (see S5 Dataset) to confirm the linkage of the correct gene(s) to each reaction. Only when all reactions necessary for production of protein were added to the reconstruction, as determined by the model being able to produce protein and convert it to biomass in FBA simulations, was the next biomass precursor (e.g., DNA) added to the biomass reaction. This process was repeated until all biomass components (S3 Table) could be produced by the model and combined into biomass. As the final model was required to grow, where necessary, reactions required to complete essential pathways were added to the core model even if the associated gene(s) were not essential.

**Fig 5:**
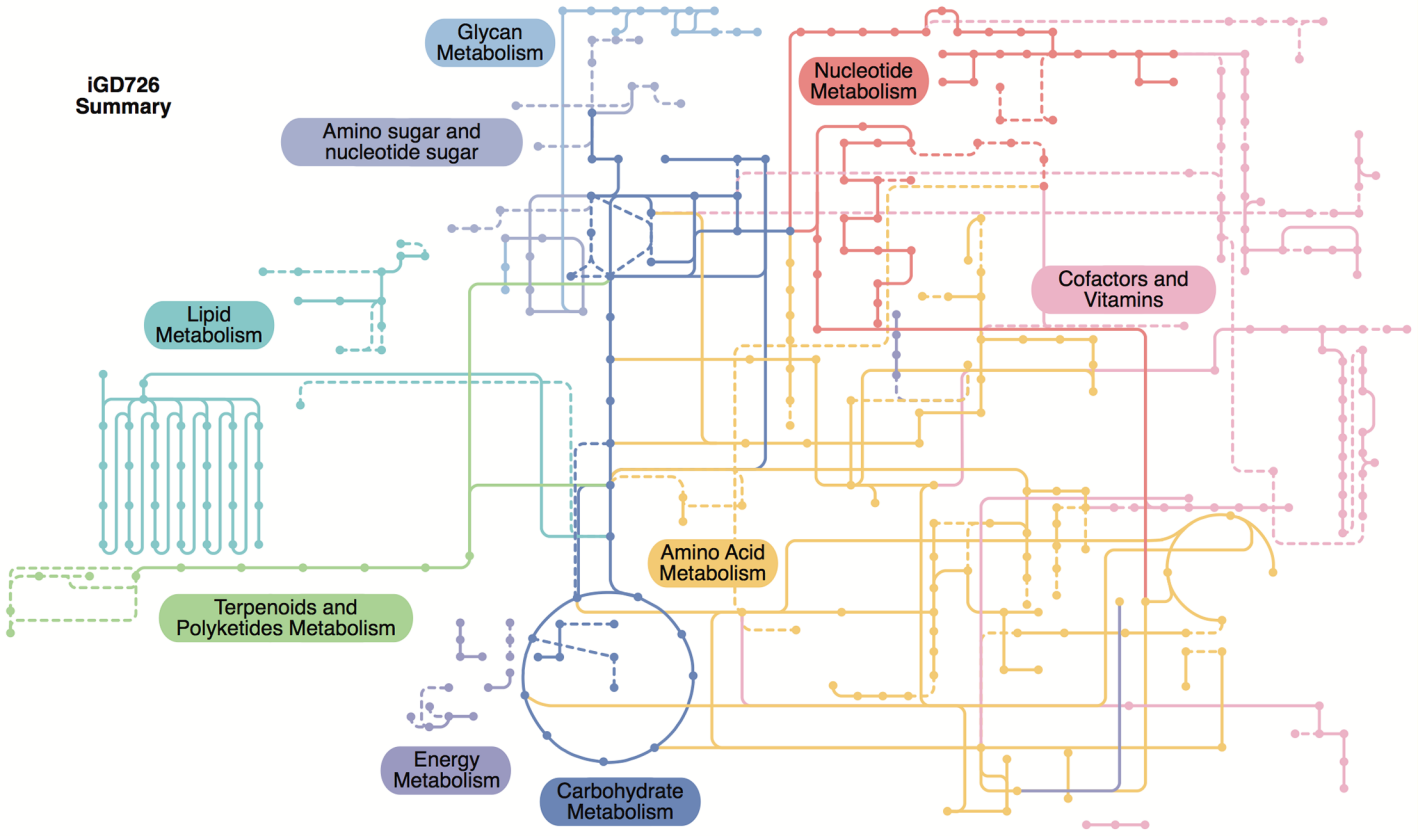
Summary schematic of core *S*. *meliloti* metabolism. The iGD726 core metabolic model was visualized using the iPath v2.0 webserver [83], which maps the reactions of the metabolic model to KEGG metabolic pathways; it therefore does not capture metabolism not present in the KEGG pathways included in iPath. Reactions and metabolites are color coded according to their biological role, as indicated. Reactions whose associated genes were not identified as growth promoting in this study are in dashed lines.

The resulting core model, termed iGD726 and included in SBML format in S2 File, is summarized in Fig 5 and Table 3, and the entire model, including genes, reaction formulas, and references is provided as an easy to read Excel table in S5 Dataset. The process of integrating the Tn-seq data with *in silico* metabolic reconstruction resulted in a major refinement of the core metabolism compared to the existing genome-scale model, while also improving predictions of gene essentiality (S13 Fig): 228 new reactions were added, 115 new genes were added, and the genes associated with 135 of the 432 reactions common to the existing genome-scale reconstruction and the new core reconstruction were updated.

**Table 3:** Summary of iGD726. The last column indicates reactions whose gene associations are supported by the Tn-seq data of this study. Percentage of all reactions in that category are indicated in brackets.

Pathways	Genes	Reactions	Reactions supported by Tn-seq
Overall	726	681	444 (65%)
Carbon metabolism, oxidative phosphorylation	105	54	37 (69%)
Amino acid metabolism	116	93	72 (77%)
Nucleotide metabolism	34	40	39 (98%)
Fatty acid, lipid metabolism	42	227	143 (63%)
Peptidoglycan, lipopolysaccharide, exopolysaccharide metabolism	47	43	27 (63%)
Nucleotide sugar metabolism	25	17	6 (35%)
Vitamin, cofactor, coenzyme metabolism	109	121	83 (69%)
Miscellaneous metabolism	23	15	7 (47%)
Transcription, translation, DNA replication, cell division	153	29	28 (97%)
Transport reactions	75	21	3 (14%)
Exchange reactions	0	21	N/A

In addition to improving the metabolic reconstruction, this process significantly expanded the view of core *S*. *meliloti* metabolism compared to that gained solely through the application of Tn-seq. The genes associated with approximately one third of the reactions in the core model were not detected as growth promoting in the Tn-seq datasets (Fig 5, Table 3). While many of the additional reactions present in the core model are due to the inclusion of non-essential biomass components, which are part of the wild-type cell but are nonetheless dispensable for growth, others are from essential metabolic pathways (Fig 5, S14 Fig). Overall, the combined approach of integrating Tn-seq data and *in silico* metabolic modeling allowed for the development of a high-quality representation of core *S*. *meliloti* metabolism in a way that neither approach alone was capable of accomplishing.

### Tn-seq-guided *in silico* metabolic reconstruction facilitates novel gene annotation

Initially, over 20 of the reactions in the core metabolic reconstruction could not be associated with a particular *S*. *meliloti* gene. Similarly, many genes with no clear biological function were found to be essential in the Tn-seq screen. By attempting to fill the gaps in the *in silico* model with the uncharacterized essential genes, we were able to assign putative functions to eight previously uncharacterized genes, of which five have good support for their new annotation (S4 Table). Two of these genes were chosen for further characterization: *smc01361* and *smc04042*. The *smc01361* gene was annotated as encoding a dihydroorotase, and targeted disruption of *smc01361* resulted in pyrimidine auxotrophy (S15 Fig). Given its location next to *pyrB*, and the presence of an essential PyrC dihydroorotoase encoded elsewhere in the genome (S1 Dataset), we propose that *smc01361* encodes an inactive dihydroorotase (PyrX) required for PyrB activity as has been observed in some other species including *Pseudomonas putida* [39,40]. The essential *smc04042* gene was annotated as encoding an inositol-1-monophosphatase family protein. It was previously observed that rhizobia lack a gene encoding a classical L-histidinol-phosphate phosphohydrolase, and it was suggested an inositol monophosphatase family protein may fulfill this function instead [41]. Targeted disruption of *smc04042* resulted in histidine auxotrophy (S15 Fig), consistent with this enzyme fulfilling the role of a L-histidinol-phosphate phosphohydrolase. It is likely that this is true for most rhizobia, as putative orthologs of this gene were identified in all 10 of the examined *Rhizobiales* genomes (S2 Dataset and S5 Dataset). These examples illustrate the power of the combined Tn-seq and metabolic reconstruction process in the functional annotation of bacterial genomes.

## Discussion

In this study, we developed a new variant of the Tn*5* transposon for construction of non-polar insertion mutations that should be readily adaptable for use with other α-proteobacteria. Although Tn*5* is generally considered to be non-specific with respect to insertion site, the consensus sequence of ~ 190,000 unique insertion locations revealed a bias for a particular GC rich motif (S3 Fig), largely consistent with previous studies [42–44]. While this bias appeared to have little effect in mutagenesis of the high-GC genome of *S*. *meliloti* (S4 Fig), accounting for this bias may be more important when applying Tn*5* mutagenesis to species with low-GC genomes.

In *S*. *meliloti*, each replicon appears to have a distinct evolutionary trajectory [45]. The pSymA replicon is a much more recent addition to the genome and is present only in a few closely related species, such as *Sinorhizobium medicae* [46]. Although present in all sequenced *S*. *meliloti* isolates, there is high genic variation between the pSymA replicons of individual strains, indicative of continual gene gain and gene loss [45,47]. This replicon is required for the symbiotic interaction with plants [48], but few other functions can be attributed to it. On the other hand, the pSymB replicon is thought to be an old addition to the genome, acquired prior to the split from *Brucella* and sharing common ancestry with the second chromosome of the genus *Brucella* and the linear chromosome of the genus *Agrobacterium* [49]. The pSymB replicon is present in all sequenced *S*. *meliloti* isolates, and generally shows high synteny between isolates [45,47]. Functionally, pSymB is notable for controlling exopolysaccharide biosynthesis [50] and for encoding numerous solute transporters and a broad range of accessory metabolic pathways [31,51], and it is likely specialized for adaptation to the rhizosphere environment [38].

Greater than 10% of bacterial species with a sequenced genome contain a genomic architecture similar to *S*. *meliloti*, that is, with at least two large DNA replicons [30,52], including many plant symbionts (e.g., the rhizobia) and plant and animal pathogens (e.g. *Burkholderia* and *Vibrio*). Several studies have revealed that, in many ways, each replicon acts as a functionally and evolutionarily distinct entity (for a review, refer to [52]); yet, there can also be regulatory interactions [53], as well as the exchange of genetic material between the replicons [54]. The Tn-seq analyses reported here provide new insights into the functional integration among replicons in a compound bacterial genome. The prevailing view is that secondary replicons such as pSymA and pSymB encode few to no essential genes. However, a large number of chromosomal genes—across many functional groups (Fig 2)—became conditionally essential following the removal of pSymA and pSymB. This demonstrates that secondary replicons actually encode many proteins able to fulfill essential cellular functions in concert with chromosomally encoded proteins. Gene functions on secondary replicons may thus remain cryptic due to functional redundancy across replicons. We hypothesize that complete functional redundancy (e.g. via gene duplication) would be a transient phenomenon, and that on an evolutionary time-scale, either i) one of the genes would be lost, ii) functional divergence would occur, or iii) regulatory divergences may occur that result in differential expression of the genes as for proline biosynthesis in *Brucella* [55]. Thus, secondary replicons have the potential to fine-tune the functionality or regulation of genes to help an organism adapt to new environments, leading to a robust genetic network encoded by both the chromosome and the secondary replicons.

Previous studies have illustrated that the fitness phenotypes of orthologous genes in related species may differ [21,23–28,56], and even that intercellular effects within microbial communities can modify the essential genome of a species [57]. The data reported here more directly address the topic of how a gene’s genotype-phenotype relationship is influenced by its genomic environment, by comparing the fitness phenotypes of mutating the exact same set of ~ 3,500 genes in two very different genomic environments. It was found that the non-essential genome had a remarkable influence on what was classified as a growth-promoting gene, with 10% of *S*. *meliloti* chromosomal genes exhibiting fitness-based genetic interactions with the non-essential component of the genome (Fig 2). This observation was not growth medium-dependent, was not unique to a specific gene functional class, and was not simply due to an overall reduced fitness of the ΔpSymAB strain as the findings could be largely replicated *in silico* (Fig 4).

**Fig 6:**
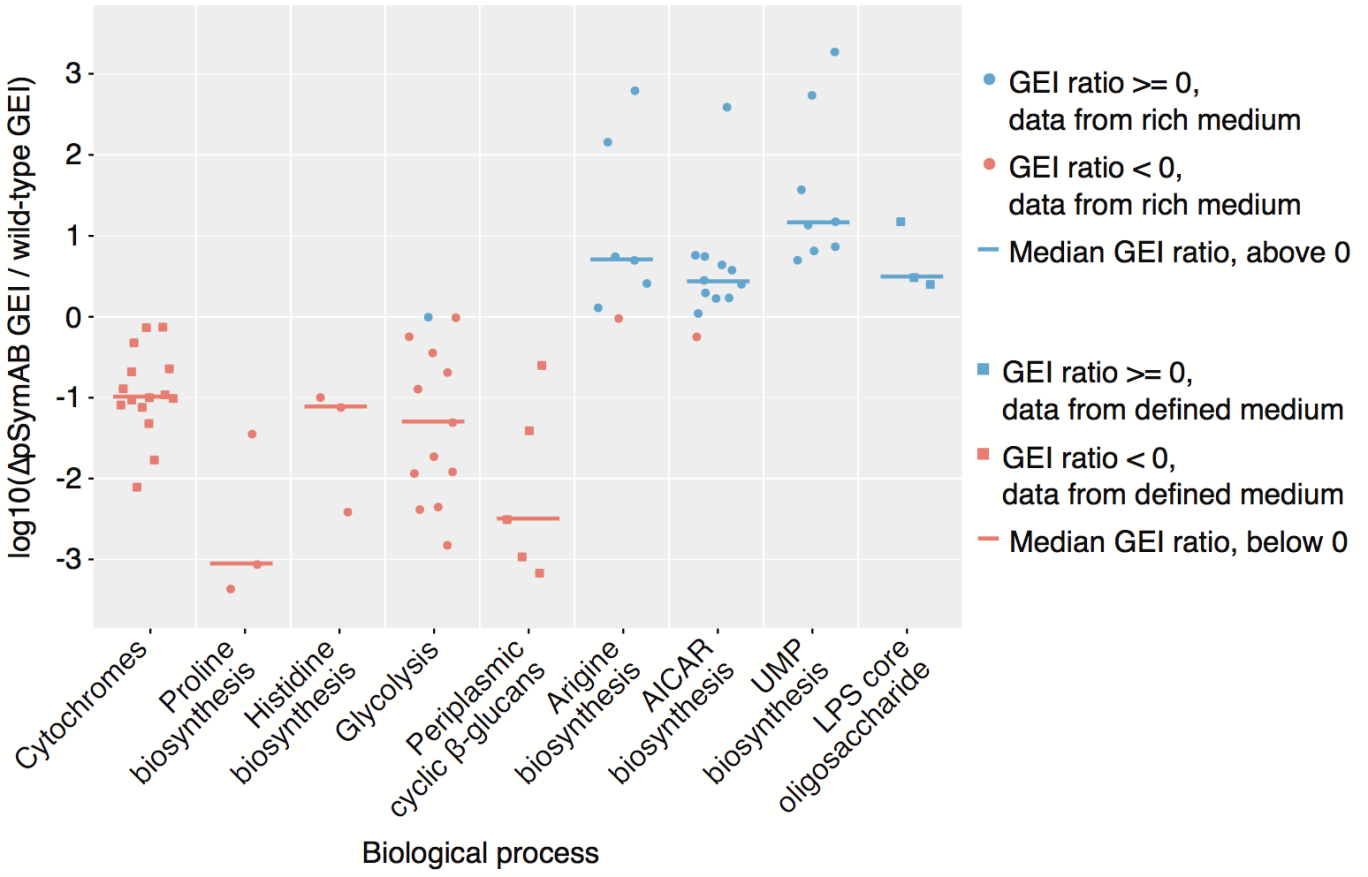
Gene essentiality index (GEI) changes for genes of selected biological pathways. Each data point represents an individual gene, and shows the log_10_ of the ratio of the GEI for that gene in the ΔpSymAB background compared to the wild-type background. Lines indicate the median value of all genes included from the biological process. The underlying data is given in S9 Table. Genes included in each process are as follows: Cytochrome C oxidase related genes–*ctaB*, *ctaC*, *ctaD*, *ctaE*, *ctaG*, *ccsA*, *cycH*, *cycJ*, *cycK*, *cycL*, *ccmA*, *ccmB*, *ccmC*, *ccmD*, *ccmG*; Proline biosynthesis–*proA*, *proB1*, *proC*; Histidine biosynthesis–*hisB*, *hisD*, *smc04042*; Glycolysis and related genes–*glk*, *frk*, *pgi*, *zwf*, *pgl*, *edd*, *eda2*, *gap*, *pgk*, *gpmA*, *eno*, *pykA*, *pyc*; Periplamic cyclic β-glucan biosynthesis–*feuN*, *feuP*, *feuQ*, *ndvA*, *ndvB*; Arginine biosynthesis–*argB*, *argC*, *argD*, *argF1*, *argG*, *argH1*, *argJ*; AICAR biosynthesis–*purB*, *purC*, *purD*, *purE*, *purF*, *purH*, *purK*, *purL*, *purM*, *purN*, *purQ*, *smc00494*; UMP biosynthesis–*carA*, *carB*, *pyrB*, *pyrC*, *pyrD*, *pyrE*, *pyrF*, *smc01361*; LPS core oligosaccharide biosynthesis–*lpsC*, *lpsD*, *lpsE*.

The majority of the genes whose fitness phenotype was dependent on the genomic environment became more important for fitness following the genome reduction. In many cases, this may reflect a loss of functional redundancy. For example, the increased importance of the chromosomal cytochrome genes (see Fig 6) likely reflects a compensation for the loss of the pSymA/pSymB encoded cytochrome complexes. In other cases, increased gene essentiality after genome reduction may reflect pathways that must compensate for loss of a non-identical housekeeping pathway. Proline and histidine biosynthesis during growth in the rich medium was specifically essential in the ΔpSymAB strain (Fig 6), possibly to compensate for the inability of this strain to transport these metabolites [33]. Similarly, glycolysis appeared specifically essential in the ΔpSymAB strain in rich medium (Fig 6), likely as the reduced metabolic capacity of this strain [31] led to a greater reliance on catabolism of the abundant sucrose in the medium used for these experiments. Specific gene essentiality in the ΔpSymAB background may also occur as a result of synthetic negative interactions not associated with direct redundancy; for example, synthetic effects of disrupting two independent aspects of the cell envelope. This is evident in the ΔpSymAB-specific essentiality of the *feuNPQ* and *ndvAB* genes involved in production of periplasmic cyclic β-glucans (Fig 6) [58–61], as the cell envelope of the ΔpSymAB strain is expected to be significantly altered relative to the wild-type [33,50,62].

Somewhat surprisingly, approximately a quarter of the genes with a genomic environment effect had a greater fitness defect in the wild-type strain. In some cases this may have been due to the reduced nutrient demand of the ΔpSymAB strain as a result of the smaller genome content. For example, mutations of genes for arginine biosynthesis and the biosynthesis of common purine and pyrimidine precursors (AICAR [5-Aminoimidazole-4-carboxamide ribonucleotide] and UMP) led to fitness defects in rich medium specifically in the wild type (Fig 6). Potentially, the uptake of these nutrients is growth-limiting to the wild type in the absence of their *de novo* synthesis, whereas this is not the case in the ΔpSymAB strain due to the reduced genome size, and thus lower nutrient requirement, and the already reduced growth rate (S8 Fig). Another possibility is that removal of pSymAB evokes phenotypes that are epistatic to many of those brought about by chromosomal mutations. For example, effects of impairing biosynthesis of the lipopolysaccharide core oligosaccharide (Fig 6) may be phenotypically masked in the absence of pSymB due to the existing cell membrane alterations brought about by removal of pSymB [33,50,62].

Our work in integrating the Tn-seq data with *in silico* metabolic modeling made it evident that Tn-seq alone is insufficient to identify the entire core metabolism of an organism. Almost a third of the reactions present in the core metabolic reconstruction were not supported by Tn-seq data (Fig 5, Table 3, S14 Fig). Conversely, the Tn-seq data supported substantial refinement of gene-reaction relationships in the model. In some cases, the gaps in the Tn-seq data were due to genomic environment effects; in other cases it was due to the inclusion of non-essential reactions that are nonetheless part of ‘wild-type’ metabolism, and sometimes the gene associated with a reaction is simply unknown. Furthermore, as Tn-seq involves growth of a complex population of mutants, there can be phenotypic complementation through cross-feeding if the metabolite is secreted and transferred to the mutant from the rest of the population.

That the Tn-seq approach fails to identify many central metabolic reactions could have a significant practical impact in synthetic biology. The results of Tn-seq studies may guide engineering of designer microbial factories with specific properties [63], or assist in the identification of putative new therapeutic targets [25,64]. While Tn-seq studies undoubtedly give invaluable information to be used toward these goals, such studies alone are insufficient as evidenced in the recent efforts to design and synthesize a minimal bacterial genome [22]. Importantly, this limitation can be overcome by combining Tn-seq with metabolic modeling. Only a few other studies have used both Tn-seq data and metabolic reconstruction [65–69], which almost always focus on using the Tn-seq data to refine the metabolic reconstruction. As illustrated here, combining an experimental Tn-seq approach with a ground-up *in silico* metabolic reconstruction strategy can also improve the interpretation of Tn-seq data. A Tn-seq-guided reconstruction forces the identification of missing essential reactions, improves gene-reaction associations, and can facilitate functional annotation of uncharacterized genes. This process allows one to obtain a very high-quality representation of the metabolism (and underlying genetics) of an organism in the given environment. The resulting model can serve as a blueprint for understanding the workings of the cell in its native state, and for engineering new cell-based factories.

## Materials and methods

### Bacterial strains, media, and growth conditions

The wild-type and ΔpSymAB strains used throughout this work are the RmP3499 and RmP3496 strains, respectively, whose construction was described previously [34]. All *E*. *coli* or *S*. *meliloti* strains used in this study are described in S5 Table and were grown at 37°C or 30°C, respectively. BRM medium was used as the rich medium for growth of the *S*. *meliloti* strains, and it consisted of 5 g/L Bacto Tryptone, 5 g/L Bacto Yeast Extract, 50 mM NaCl, 2 mM MgSO_4_, 2 μM CoCl_2_, 0.5% (w/v) sucrose, and supplemented with the following antibiotics, as appropriate: streptomycin (Sm, 200 μg/ml), neomycin (Nm, 100 μg/ml), gentamycin (Gm, 15 μg/ml). The defined medium for growth of *S*. *meliloti* contained 50 mM NaCl, 10 mM KH_2_PO_4_, 10 mM NH_4_Cl, 2 mM MgSO_4_, 0.2 mM CaCl_2_, 0.5% (w/v) sucrose, 2.5 μM thiamine, 2 μM biotin, 10 μM EDTA, 10 μM FeSO_4_, 3 μM MnSO_4_, 2 μM ZnSO_4_, 2 μM H_3_BO_3_, 1 μM CoCl_2_, 0.2 μM Na_2_MoO_4_, 0.3 μM CuSO_4_, 50 μg/ml streptomycin, and 30 μg/ml neomycin. *E*. *coli* strains were grown on Luria-Bertani (LB) medium supplemented with the following antibiotics as appropriate: chloramphenicol (30 mg/ml), kanamycin (Km, 30 μg/ml), gentamycin (Gm, 3 μg/ml).

### Growth curves

Overnight cultures grown in rich medium with the appropriate antibiotics were pelleted, washed with a phosphate buffer (20 mM KH_2_PO_4_ and 100 mM NaCl), and resuspended to an OD_600_ of 0.25. Twelve μl of each cell suspension was mixed with 288 μl of growth medium, without antibiotics, in wells of a 100-well Honeycomb microplate. Plates were incubated in a Bioscreen C analyzer at 30°C with shaking, and OD_600_ recorded every hour for at least 48 hours.

### *S*. *meliloti* mutant construction for Tn-seq validation

Single-gene knockout mutants were generated through single cross-over plasmid integration of the suicide plasmid pJG194 [70]. Approximately 400-bp fragments homologous to the central portion of the target genes were PCR amplified using the primers listed in S6 Table. PCR products as well as the pJG194 vector were digested with the restriction enzymes *Eco*RI/*Hind*III, or *Sal*I/*Xho*I, and each PCR fragment was ligated into appropriately digested pJG194 using standard techniques [71], and all recombinant plasmids were verified by Sanger sequencing. Recombinant plasmids were mobilized from *E*. *coli* to *S*. *meliloti* via tri-parental matings as described [60], and transconjugants were isolated on BRM Sm Nm agar plates. All *S*. *meliloti* gene disruption mutants were verified by PCR.

Transduction of the integrated plasmids into the *S*. *meliloti* wild-type and ΔpSymAB strains was performed using bacteriophage N3 as described elsewhere [72], with transductants recovered on BRM medium containing the appropriate antibiotics.

### Construction of the transposon delivery vector pJG714

The plasmid pJG714 is a variant of the previously reported mini-Tn*5* delivery plasmid, pJG110 [70], with the primary modifications being removal of the *bla* gene and pUC origin of replication, and introduction of the *pir*-dependent R6K replication origin. A map of pJG714 is given in S1A Fig, and the complete sequence of the transposable region is provided in S1B Fig. This delivery plasmid is maintained in *E*. *coli* strain MFD*pir* [73], which possesses chromosomal copies of R6K *pir* and RK2 transfer functions. MFD*pir* is unable to synthesize diaminopimelic acid (DAP), thus disabling growth on rich or defined medium lacking supplemental DAP. The MFD*pir*/pJG714 strain was cultured on rich medium containing kanamycin and 12.5 μg/ml DAP.

### Tn-seq experimental setup

Transposon mutagenesis was accomplished in the wild-type and ΔpSymAB strains in parallel. Flask cultures of MFD*pir*/pJG714 and the two *S*. *meliloti* strains were grown overnight to saturation, and pellets were washed and suspended in BRM to a final OD_600_ value of approximately 40. Equal volumes of each suspension were mixed as bi-parental matings, to accomplish mobilization of the transposon delivery vector into the *S*. *meliloti* recipient strains. These cell mixtures were plated on BRM supplemented with 50 μg/ml DAP and incubated at 30°C for 6 h. Mating mixtures were collected in BRM with 10% glycerol, and cell clumps were broken up by shaking the suspended material for 30 min at 225 rpm. Aliquots were stored at -8°C. For selection of transposants, mating mixes were thawed and plated at a density of 15,000 cfu/plate (150-mm plates) on BRM supplemented with Sm and Nm. To accomplish equivalent coverage of each genome with transposon insertions, 675,000 and 360,000 colonies were selected for the wild-type and ΔpSymAB strains, respectively. For each recipient, transposon mutant colonies were collected and cell clumps were broken up as described above. The selected clone libraries were aliquoted and stored at -80°C.

For whole-population selection and massively parallel sequencing of transposon ends, 1x10^9^ cells from each of the two clone libraries were transferred into 500 ml of either BRM or defined medium, allowing approximately 8–10 generations of growth at 30°C before reaching saturation. At this stage, cells were pelleted, DNA was extracted using the MoBio Microbial DNA isolation kit (#12255–50), and the resulting DNA was fragmented with NEB fragmentase (#M0348S) to an average molecular weight of 1000 bp. After clean-up (Qiagen #27106), the resulting DNA fragments were appended with short 3’ homopolymer (oligo-dCTP) tails using terminal deoxynucleotidyl transferase (NEB #M0315S), and this sample was used as the template for a two-round PCR process that gave rise to the final Illumina-ready libraries. In the first round, a transposon end-specific primer (1TN) and oligo-G primer (1GG) were used (all primer sequences can be found in S6 Table). After clean-up, a portion of the first-round product was used as the template for the second-round reaction employing a nested transposon-specific primer (2TNA-C) and a reverse index-incorporating primer (2BAR01-08). A series of three 2TN primers (A-C) were designed to incorporate base diversity in the opening cycles of Illumina sequencing, and a series of eight 2BAR primers were designed to uniquely identify each experimental condition in a single multiplexed sequencing sample. After PCR amplification of transposon-flanking sequences with concomitant incorporation of Illumina adapters and barcodes, the samples were size-selected for 200-600-bp fragments, and sequenced on an Illumina Hi-Seq instrument as 50-bp single-end reads. Raw sequencing data was deposited to the Sequence Read Archive (SRA) as part of a Bioproject (accession: PRJNA427834).

### Tn-seq data analysis and calculation of gene essentiality indexes

Raw DNA-sequencing reads were used as input into a custom-built Tn-seq analytical pipeline, which was recently described [64]. In brief, the pipeline first processed the fastq files and discarded reads not containing the transposon sequence. It then aligned reads to the genome with Bowtie2 [74], counted the number of reads to each annotated gene in the genome, and normalized the results based on reads mapping to intergenic regions. Reads mapping to the last 5% of the gene were discarded [75], and all other options were left at their default settings.

The normalized read counts were used as a proxy of the transposon insertion density within each gene. To calculate the Gene Essentiality Index (GEI) scores, a pseudo count of one was first added to all normalized gene read counts for each replicate. GEI scores were then calculated by summing the number of reads that mapped to the gene in both replicates, and dividing this number by the nucleotide length of the gene. GEI scores were calculated for each gene separately in each medium and in each strain. All GEI values are available in S1 Dataset, as are fold changes between conditions.

### Statistical analysis of the Tn-seq output

The output of the Tn-seq analysis pipeline for the chromosomal genes was used in the fitness classification of genes as detailed further in the Supplementary Methods of S1 File. Briefly, all genes with no observed insertions (i.e., no reads) were classified as essential. Although this step may result in small genes (that lack insertions by chance) being falsely annotated as essential, manually checking these genes showed many are expected to be truly essential (e.g. ribosomal proteins). Next, GEI scores were imported into R version 3.2.3 and log transformed. Initial clustering of the data was performed through the fitting of an optimal number of overlapping Guassian distributions to the log transformed GEI scores (S16 Fig), using the *Mclust* function of the *Mclust* package in R [76]. Clusters were then refined through the use of the affinity propagation statistical approach, implemented in the *apcluster* function of the *apcluster* package of R [77]. Genes with GEI scores significantly different between conditions were determined through clustering of the log transformed fold changes and the fitting of overlapping Guassian distributions with *Mclust* in R (S16 Fig).

Subsequent to the above analyses, the validity of the clustering-based approach in identifying essential genes was examined by re-analyzing the data with the recently published TSAS pipeline [69]. The pipeline was run independently for each sample (both replicates were considered together) using the one-sample analysis setting and the default parameters. The TSAS pipeline uses binomial probability to identify the probability that a gene has fewer insertions than expected by chance, but does not distinguish between essential and growth defective genes. Output from this tool is included in S1 Dataset, and overall, the analysis i) supported the gene classifications determined using the clustering approach above, and ii) supported that the general conclusions drawn from this work are unlikely to be significantly impacted by choice of analysis pipeline. Additional description of the comparison of the methods can be found in the Supplementary Results of S1 File.

### Gene functional enrichments

Assignment of chromosomal genes into specific functional categories was performed largely based on the annotations provided in the *S*. *meliloti* Rm1021 online genome database (iant.toulouse.inra.fr/bacteria/annotation/cgi/rhime.cgi). This website pulls annotations from several databases including PubMed, Swissprot, trEMBL, and Interpro. Additionally, it provides enzyme codes, PubMed IDs, functional classifications, and suggested Gene Ontology (GO) terms for most genes. The numerous classifications were simplified to 18 functional categories, designed to adequately cover all core cellular processes, as indicated in the Supplementary Methods of S1 File. In cases of ambiguous or conflicting annotations, the annotations were refined through an approached based on BLASTp searches, as described in the Supplementary Methods of S1 File. The functional annotations of all chromosomal genes are provided in S6 Dataset.

### Data visualization

Tn-seq results were visualized using the Integrative Genomics Viewer v2.3.97 [78]. Scatter plots, functional enrichment plots, box plots, and line plots were generated in R using the *ggplot2* package [79]. Venn diagrams were produced in R using the *VennDiagram* package [80]. The genome map was prepared using the circos v0.67–7 software [81]; the sliding window insertion density was calculated with the *geom_histogram* function of *ggplot2*, and the GC skew was calculated using the analysis of sequence heterogeneity sliding window plots online webserver [82]. The metabolic model was visualized using the iPath v2.0 webserver [83]. The logo of the transposon insertion site specificity was generated by first extracting the nucleotides surrounding all unique insertion sites in one replicate of the wild type grown in rich medium using Perl v5.18.2, followed by generation of a hidden Markov model with the *hmmbuild* function of HMMER v3.1b2 [84] and visualization with the Skylign webserver [85].

### Blast Bidirectional Best Hit (Blast-BBH) strategy

Putative orthologous proteins between species were identified with a Blast-BBH approach, implemented using a modified version of our in-house Shell and Perl pipeline [86]. Proteomes were downloaded from the NCBI repository, and the Genbank annotations were used. To limit false positives, Blast-BBH pairs were only maintained if they displayed a minimum of 30% amino acid identify over at least 60% of the protein. To identify putative, functionally duplicated proteins in *S*. *meliloti*, the same Blast approach was employed to compare the *S*. *meliloti* chromosomal proteome with the proteins encoded by pSymA and pSymB. The Blast-BBH approach was used to identify putative orthologs of all *S*. *meliloti* proteins in 10 related species within the order *Rhizobiales*. All results are given in S2 Dataset and S5 Dataset in order to facilitate easy identification of the Tn-seq data and metabolic reconstruction data for a *S*. *meliloti* ortholog of a gene of interest in these other species.

### *In silico* metabolic modeling procedures

All simulations were performed in MATLAB 2017a (Mathworks) with scripts from the COBRA Toolbox (downloaded May 12, 2017 from the openCOBRA repository) [87], and using the Gurobi 7.0.2 solver (gurobi.com), the SBMLToolbox 4.1.0 [88], and libSBML 5.13.0 [89]. Boundary conditions for simulation of the defined medium are given in S7 Table. Single and double gene deletion analyses were performed using the *singleGeneDeletion* and *doubleGeneDeletion* functions, respectively, using the Minimization of Metabolic Adjustment (MOMA) method. All MATLAB scripts used in this work are provided as S3 File. For all deletion mutants, the growth rate ratio (grRatio) was calculated as: growth rate of mutant / growth rate of wild-type. Single gene deletion mutants were considered to have a growth defect if the grRatio was < 0.9. For the double gene deletion analysis, if the grRatio of the double mutant was less than 90% of the expected grRatio (based on multiplying the grRatio of the two corresponding single mutants), the double deletion was said to have a synthetic negative phenotype.

### Preparation of the genome-scale metabolic network reconstructions

*In silico* analysis of redundancy in the *S*. *meliloti* genome was performed using the existing *S*. *meliloti* genome-scale metabolic network reconstruction [38], modified as described in the Supplementary Methods of S1 File. Removal of pSymA and pSymB *in silico* is described in the Supplementary Methods of S1 File. A draft, fully automated model containing no manual curation for *R*. *leguminosarum* bv. *viciae* 3841 was built using the KBase webserver (kbase.us), based on the Genbank file of the *R*. *leguminosarum* genome [90]. Similarly, a draft *S*. *meliloti* Rm1021 model was built using KBase, starting with the Genbank file for the *S*. *meliloti* genome [29]. Draft model reconstruction is described further in the Supplementary Methods of S1 File. All metabolic reconstructions used in this work are provided in SBML and MATLAB format in S2 File.

### Building the *S*. *meliloti* core metabolic reconstruction, iGD726

The iGD726 core model was built from the ground-up using the existing genome-scale model as a reaction and GPR database, and with the Tn-seq data as a guide. This process is described in detail in the Supplementary Methods of S1 File. Briefly, iGD726 began with no reactions except for a biomass reaction that contained only a single substrate (e.g., protein). All pathways required to produce protein were then added to the core model, using the Tn-seq data as a guide and drawing reactions from the original genome-scale model, or when necessary, from the Kyoto Encyclopedia of Genes and Genomes (KEGG) database [91]. When all reactions necessary for the production of protein were present in the model, as confirmed by the ability of the model to produce biomass in FBA simulations, the next biomass component was added to the biomass reaction. This process was repeated until the core model could produce all biomass components (S3 Table). As the original model is a full genome-scale metabolic reconstruction, encompassing core and accessory metabolism, not all reactions were transferred to the core model; only those essential for biomass production or to accurately capture the Tn-seq data were included in iGD726. Throughout the above process, the Tn-seq data were used to refine the gene-reaction associations. Each time a new reaction was added to the core reconstruction, the genes associated with the reaction were checked against the Tn-seq data, and a literature search for each associated gene was performed. The gene associations were then modified as necessary to ensure the model accurately captured the experimental data. Additionally, putative functions were assigned to uncharacterized genes during model construction by trying to link essential Tn-seq genes to essential reactions lacking an associated gene, as described in the Supplementary Methods of S1 File.

The final model contained 726 genes, 681 reactions, and 703 metabolites, and is provided in SBML and MATLAB format in S2 File, and as an Excel file in S5 Dataset. The Excel file contains all necessary information for use as a *S*. *meliloti* metabolic resource, including the reaction name, the reaction equation using the real metabolite names, the associated genes/proteins, and references. Additionally, for each reaction, the putative orthologs of the associated genes in 10 related *Rhizobiales* species are included, allowing the model to provide useful information for each of these organisms.

## Supporting information

S1 File Supplementary text. This file contains the Supplementary Text, the associated references, and the references for S5 Dataset.
(PDF)

S2 File Metabolic reconstructions prepared in this study. Contains all metabolic reconstructions that were prepared in this study in both SBML format and in COBRA format, saved as a MATLAB file. A *README* file is included in the archive to explain the contents of the file.
(ZIP)

S3 File MATLAB script for constraint-based modeling. A MATLAB script for replicating the constraint-based modeling analyses of this study is provided.
(TXT)

S1 Table Tn-seq library coverage. (PDF)

S2 Table Generation times (hours) of *S*. *meliloti* strains. (PDF)

S3 Table Core model (iGD726) biomass composition. (PDF)

S4 Table Newly predicted functions for eight genes previously hypothetical or broadly annotated genes. (PDF)

S5 Table Bacterial strains and plasmids. (PDF)

S6 Table Oligonucleotides used in this study. (PDF)

S7 Table Boundary conditions for *in silico* metabolic modeling analyses. (PDF)

S8 Table Biomass composition of iGD1575b and iGD1575c. (PDF)

S9 Table Gene essentiality data represented in Fig 6. (PDF)

S1 Fig Description of the engineered transposon Tn5-714. (**A**) Genetic map of the delivery vector pJG714, indicating the locations of the transposase-encoding gene (*tnp*), transposon ends (TE), the kanamycin/neomycin resistance determinant (*kan/neo*), the *Salmonella trp* promoter (P*trp*), the *cis*-acting mobilization determinant from plasmid RK2 (*oriT*), and the *pir*-dependent replication origin from plasmid R6K (*oriV*). The transposable region is highlighted in grey. No transcriptional stop sequences are present between the *trp* or *kan/neo* promoters and the downstream TEs. (**B**) Sequence of Tn*5*-714 (highlighted in grey in A), indicating the transposon ends (red), P*trp* and *kan/neo* promoters (dotted arrows), the *kan/neo* coding region (green, underlined), and the oligo-C region synthesized in the process of Illumina library preparation. Primers used for amplification of transposon-genome junctions, and for appending the appropriate Illumina adapter sequences, are shown, with first-round primers named with the “1” prefix and second-round primers named with the “2” prefix. Asterisks indicate the location of 6-nt barcodes used to identify each experimental sample after multiplexed sequencing. Actual primer sequences are given in S6 Table.
(TIF)

S2 Fig Distribution of transposon insertions along the *S*. *meliloti* chromosome. Read counts for each Tn-seq replicate were normalized by applying a multiplicative factor such that each replicate had an equal number of total reads. The reads for each replicate were the grouped into 25,000 base pair bins, with each bin shifted by 5,000 bp. The value of each bin across all eight replicates were summed, giving the final total normalized read count in each 25,000 base pair bin. The results of this analysis are provided in this figure. The X-axis represents the *S*. *meliloti* chromosome, starting at nucleotide position one in the RefSeq genome annotation. The Y-axis indicates the total number of normalized read counts in each 25,000 base pair bin.
(TIF)

S3 Fig Logo of the transposon insertion sequence specificity. The location of each chromosomal transposon insertions in one replicate of the wild-type strain grown in rich medium was determined, and 186,102 unique positions were identified. Shown are the 10 nucleotides before the insertion site (-10 to -1), the 9 nucleotides that are part of the duplicated region (D1 to D9), and the 10 nucleotides following the insertion site (+1 to +10). The logo was generated from the hidden Markov model of the 186,102 unique insertion positions. The enrichment of G and C in the left and right ends of the logo are simply a consequence of the high GC content of the genome.
(TIF)

S4 Fig Effect of GC content on the Tn-seq output. (**A**) These box and whisker plots summarize the distribution of the GC content of all chromosomal genes (left), the 489 core growth promoting genes (middle), and the 307 core essential genes (right). (**B**) A scatter plot showing the Gene Essentiality Index (GEI) scores for each gene against the GC content of the genes. The R^2^ value and the residual standard errors are given.
(TIF)

S5 Fig Independent validation of Tn-seq essential genes. Four genes identified as essential based on the Tn-seq output were independently validated by attempting to disrupt the coding sequences through single cross-over plasmid integration. Three plasmids were designed for each gene, two that would disrupt the coding sequence (KO1 and KO2), and one that would not disrupt the coding sequence (ND) as a control. The locations of each construct are shown on the left. Each plasmid was transferred to wild-type *S*. *meliloti* via conjugation, and transconjugants selected on rich medium. Pictures of the selective plates are shown on the right. In all cases, colonies were only observed for conjugations involving the ND plasmids, validating what was observed in the Tn-seq data.
(TIF)

S6 Fig Reproducibility of the Tn-seq data. Duplicate experiments were performed for each strain in each medium. The insertion density of each gene in each experiment was calculated as the number of insertions per nucleotide. These graphs plot the insertion density of the two duplicates of each experiment. The linear regression line of the data is shown in red, and the adjusted R^2^ values and the residual standard errors are shown in the top left of each graph. Depending on the condition, between 9 and 34 genes had an average of more than 5 reads per nucleotide, and these genes were excluded from these graphs. (**A**) Wild-type grown in the rich medium, (**B**) ΔpSymAB grown in the rich medium, (**C**) wild-type grown in the defined medium, and (**D**) ΔpSymAB grown in the defined medium.
(TIF)

S7 Fig Expression level of the Tn-seq identified growth promoting genome. The RPKM values for all *S*. *meliloti* genes (including the chromosome, pSymB, and pSymA) when grown in a glucose minimal medium were extracted from the dataset of diCenzo *et al*. [94]. Genes were assigned to a percentile value, with higher expressed genes belonging to higher percentiles. These box and whisker plots summarize the distribution of expression values for all chromosomal genes (left), and for the 489 core growth promoting genes (right) as identified in this study.
(TIF)

S8 Fig Growth profiles of wild-type and ΔpSymAB. Growth profiles are shown for the wild-type (RmP3499) and ΔpSymAB (RmP3496) *S*. *meliloti* strains grown in (**A**) rich medium or (**B**) defined medium. Data points represent the average of triplicate samples, and do not have the blank value subtracted.
(TIF)

S9 Fig Growth medium effect on the fitness phenotype of gene disruptions in ΔpSymAB. (**A**) A scatter plot comparing the fitness phenotypes of ΔpSymAB grown in rich medium versus ΔpSymAB grown in defined medium. (**B**) Functional enrichment plots for the indicated gene sets. Name abbreviations: Fit–fitness; Dec–decrease; ΔAB—ΔpSymAB; Def–defined medium; Rich–rich medium. For example, ‘Fit. dec. ΔAB def > rich' means the genes with a greater fitness decrease in ΔpSymAB grown in defined medium compared to rich medium. Legend abbreviations: AA–amino acid; Attach–attachment; Carb–carbohydrate; Cofact–cofactor; e-–electron; Met–metabolism; Misc–miscellaneous; Mot–motility; Nucl–nucleotide; Oxidoreduct–oxidoreductase activity; Prot–protein; Trans–transduction.
(TIF)

S10 Fig Validation of the Tn-seq determined strain specific phenotypes. Knockout mutations, generated through single cross-over plasmid integration, of 17 genes were prepared. Numbers shown are the CFU/mL of transductions (based on both large and small colonies), and the % CFU/mL of the *rhaK* control is indicated. All transductions were performed at least two independent times, and numbers from a representative experiment are shown. These included one control gene (*rhaK*) whose mutation was expected to have no phenotype in these conditions, 14 genes identified as specifically required in the ΔpSymAB based on the Tn-seq data, and two genes (*carA*, *coaA*) identified as specifically required in the wild-type for growth on rich media based on the Tn-seq data. All mutations were originally made in the wild-type background, except for *carA* that was made in the ΔpSymAB background. Mutations were then moved by transduction into both the wild-type and ΔpSymAB strains, and transductants recovered on selective media. Rich medium selection plates from the transductions are shown. Plates involving the ΔpSymAB strain were incubated for two days longer than those involving the wild-type.
(TIF)

S11 Fig Growth curves for gene mutants with a Tn-seq fitness phenotype specific to ΔpSymAB. Growth curves are shown for *S*. *meliloti* strains with mutations of the seven genes determined to be specifically required in the ΔpSymAB strain based on the Tn-seq data, but that were non-essential when independently mutated based on the transduction experiment (S10 Fig). The ‘no mutation’ genotype refers to the wild-type or ΔpSymAB strains without additional mutations, and the *rhaK* strain is a control mutation expected to have no effect on growth. Data points represent the average of triplicate samples and do not have the blank value subtracted, while the error bars show the standard error. Growth is shown for (**A**) wild-type derivatives grown in rich medium, (**B**) wild-type derivatives grown in defined medium, (**C**) ΔpSymAB derivatives grown in rich medium, and (**D**) ΔpSymAB derivatives grown in defined medium.
(TIF)

S12 Fig Growth rate correlation between *in silico* chromosomal reaction deletion mutants of wild-type and ΔpSymAB *S*. *meliloti* strains. Each reaction dependent on a chromosomal gene in the *S*. *meliloti* genome-scale metabolic model was deleted in the presence and absence of pSymA/pSymB dependent reactions, and the effect on growth recorded as a grRatio value (growth rate of mutant / growth rate of non-mutant). The grRatio of the reaction deletions in the presence (X-axis) compared to the absence (Y-axis) of the pSymA/pSymB reactions was plotted, and the points binned using hexagonal binning. Reactions whose deletion had no effect in both models or whose deletion was lethal in both models are not included in this figure. The color of each hexagon is representative of the number of reactions plotted at that location, as illustrated by the density bar at the bottom of the figure. The diagonal line serves as a reference line for reactions having equal effect in both conditions.
(TIF)

S13 Fig Overlap between experimental and *in silico* data. Venn Diagrams are shown to illustrate the extent of overlap between different experimental and *in silico* datasets. All Tn-seq data are taken for strains grown in defined medium. (**A**) For chromosomal genes included in iGD1575 (the original genome-scale *S*. *meliloti* metabolic model), the overlap between those observed to have a synthetic negative phenotype in the absence of pSymA and pSymB as determined with Tn-seq and metabolic modeling is shown. (**B**) For chromosomal genes included in iGD1575, the overlap between those observed as important to growth (group I and II genes) in the Tn-seq dataset with those predicted by metabolic modeling to result in at least a 50% decrease in growth rate when deleted in iGD1575 are shown. (**C**) For chromosomal genes included in iGD1575, the overlap between those observed as important for growth (group I and II genes) in the Tn-seq dataset with those predicted by metabolic modeling to result in at least a 50% decrease in growth rate when deleted in iGD726 (the core model) is shown.
(TIF)

S14 Fig Amino acid biosynthesis in *S*. *meliloti*. A summary schematic of the amino acid biosynthetic pathways of *S*. *meliloti* are shown, based on the iGD726 core metabolic reconstruction. Central carbon metabolism is displayed in grey. The 20 amino acids and important precursor compounds are labeled. Genes associated with the reactions in solid blue lines were supported by the Tn-seq data of this study, while the genes associated with the reactions in dashed red lines were not supported by the Tn-seq data of this study.
(TIF)

S15 Fig Auxotrophy analysis of *S*. *meliloti* single gene knockouts. Growth curves of five *S*. *meliloti* mutants are shown in minimal medium supplemented with the indicated nutrients. ‘Defined medium’ indicates the medium was not supplemented with any nutrients aside from the base defined medium composition, while other designations indicate which nutrient(s) were added to the base defined medium. BCAA stands for ‘branched chain amino acids’. Data points represent the average of triplicate samples and do not have the blank value subtracted, while the error bars show the standard error. (**A**-**D**) The mutations are in the wild-type (RmP3499) background. (**E**) The mutation is in the ΔpSymAB (RmP3496) background as the *argD* mutation does not result in auxotrophy in the wild-type background.
(TIF)

S16 Fig Sample data analysis with the *Mclust* function. Sample input data and clustering as determined using *Mclust* is shown. In each figure, the bars represent a histogram that shows the complete distribution of the data. The smooth red line is the estimated density as calculated by *Mclust* based on the data distribution. The colored lines along the top represent the clustering as performed by *Mclust*; individual lines represent individual genes, with the color indicating the different clusters (names of the clusters are indicated). (**A**) Data and clustering for the Gene Essentiality Index (GEI) scores for the wild-type grown in the rich medium. (**B**) Data and clustering of the fold changes in the GEI scores of genes in the wild-type when grown in defined medium relative to rich medium. (**C**) Data and clustering of the fold changes in the GEI scores of genes in the ΔpSymAB strain relative to the wild-type strain when grown in defined medium.
(TIF)

S1 Dataset Tn-seq output summary. This file contains the output and summary indexes of all Tn-seq experiments, for all three replicons, reported in this study. A *legend* sheet is included to explain the contents of the file.
(XLSX)

S2 Dataset Summary of the Blast-BBH search between *S*. *meliloti* and related *Rhizobiales*. This file contains a list of putative orthologs for all *S*. *meliloti* proteins encoded by 10 other species of the family *Rhizobiales*. A *legend* sheet is included to explain the contents of the file.
(XLSX)

S3 Dataset Comparison of the core essential genomes of *S*. *meliloti* and *R*. *leguminosarum*. This file includes information on the core essential genomes of *S*. *meliloti* and *R*. *leguminosarum*, the overlap between the core genes, and the *in silico* predicted phenotypes where applicable. A *legend* sheet is included to explain the contents of the file.
(XLSX)

S4 Dataset Summary of the Blast-BBH search between the *S*. *meliloti* chromosome and pSymA/pSymB proteomes. This file contains a list of Blast-BBH hits when the *S*. *meliloti* chromosomal proteome was compared against the combined *S*. *meliloti* pSymA/pSymB proteome. A *legend* sheet is included to explain the contents of the file.
(XLSX)

S5 Dataset Excel file of the *S*. *meliloti* core metabolic network reconstruction iGD726. This file contains an Excel formatted version of the iGD726 core metabolic network reconstruction. A *legend* sheet is included to explain the contents of the file. All references present in this file are listed in S1 File.
(XLSX)

S6 Dataset Functional annotation of the *S*. *meliloti* chromosome. The functional annotation of each gene on the *S*. *meliloti* chromosome as determined in this study. A *legend* sheet is included to explain the contents of the file.
(XLSX)

S7 Dataset Output of the *in silico* gene and reaction deletion analyses. This file contains output related to the *in silico* gene and reaction deletion analyses that form the basis of Fig 4 and S12 Fig. A *legend* sheet is included to explain the contents of the file.
(XLSX)
