# Supplementary material for: Robustness encoded across essential and accessory replicons of the ecologically versatile bacterium Sinorhizobium meliloti
Source: PLoS Genet. 2018 Apr 19;14(4):e1007357. doi: 10.1371/journal.pgen.1007357 (PMC5929573; doi:10.1371/journal.pgen.1007357)
Supplement: S1 File — This file contains the Supplementary Text, the associated references, and the references for S5 Dataset. (PDF) [file pgen.1007357.s001.pdf]

SUPPLEMENTARY METHODS

Statistical analysis of the Tn-seq output.

The output of the Tn-seq analysis pipeline for the chromosomal genes was used in the fitness classification of genes as follows. First, all genes with no observed insertions (i.e., no reads) were classified as essential. Although this step may result in small genes (that lack insertions by chance) being falsely annotated as essential, manually checking these genes showed many are expected to be truly essential (e.g. ribosomal proteins), supporting the inclusion of this step. Next, GEI scores were imported into R version 3.2.3 and log transformed. Initial clustering of the log transformed GEI scores into fitness categories was performed using the *Mclust* function of the *Mclust* package in R [1]. In short, this function attempted to explain the distribution of GEI values by fitting a series of overlapping Guassian distributions, with the number and shape of the distributions determined by *Mclust* (Bayesian Information Criteria was used to determine the optimal number of distributions [and hence clusters] that best explains the data). The data were then assigned to different categories based on the probability of the data point arising from each of the distributions. Sample output data from *Mclust* showing the distribution of the data and the clustering is provided as Figure S16. As high uncertainty in the classification of genes at the borders of groupings exists, the clusters were refined through the use of affinity propagation implemented by the *apcluster* function of the *apcluster* package of R [2], which groups data points in a method independent of *k-means* or Gaussian distributions. All genes belonging to the same group as determined by affinity propagation implemented in *apcluster* that contained an essential gene, as determined in any of the previous steps, were re-annotated as essential. Additionally, all genes belonging to the same group in the *apcluster* output that spanned the border of two groups as determined by *Mclust* were transferred to the same classification, based on which cluster the genes had a higher median probability of being derived from in the *Mclust* analysis. Finally, genes that were classified as 'essential' in one medium and 'large growth impairment' in the second medium, but that were identified as having no medium specificity based on clustering of the fold changes between media, were considered as essential in both media. Few genes were impacted by this final step, and these were those that straddle the threshold between these two fitness categories. The reclassification was performed based on the logic that a gene cannot i) contribute to growth equally in both media but ii) be essential in only one of the two media.

Genes with GEI scores significantly different between conditions were determined as follows. The GEI fold changes between the conditions of interest were imported into R, log transformed, and the following clustering performed independently for each fold change comparison. The log transformed fold changes were clustered using *Mclust* in R as described above for the GEI scores. In the case of the media comparisons, three clusters were produced by *Mclust*: 'Little to no difference', 'Moderate difference', and 'Large difference'; only genes with a fold change classified as 'Large difference' were considered to display growth medium specificity. In the case of comparing between strains, only two clusters were produced by *Mclust*: 'Little to no difference' and 'Difference between strains'. Sample output data from *Mclust* showing the distribution of the fold change data and the clustering is provided in Figure S16, which helps to illustrate why there is a difference in the number of categories for these comparisons. These clusters were then refined using *apcluster* in R as described above for the GEI scores.

Gene functional enrichments.

Assignment of chromosomal genes into specific functional categories was performed largely based on the annotations provided in the *S. meliloti* Rm1021 online genome database (https://iant.toulouse.inra.fr/bacteria/annotation/cgi/rhime.cgi). This website pulls annotations from several databases including PubMed, Swissprot, trEMBL, and Interpro. Additionally, it provides enzyme codes, PubMed IDs, functional classifications, and suggested Gene Ontology (GO) terms for most genes. The numerous classifications were simplified to 18 functional categories, designed to adequately cover all core cellular processes. Each category was based on a generic GO term, and the categories (with related GO terms in parentheses) were: signal transduction (GO:0007165), lipid metabolism (GO:0006629), amino acid metabolism (GO:0006520), translation (GO:0006412), electron transport (GO:0009055), transport (GO:0006810), carbohydrate metabolism (GO:0005975), nucleic acid metabolism (GO:0090304), cell division (GO:0051301), motility and attachment (GO:0048870 and GO:0022610), oxidoreductase activity (GO:0016491), cofactor metabolism (GO:0051186), DNA metabolism (GO:0006259), transcription (GO:0097659 plus GO:1903506), cell wall (GO:0042546), hypothetical (no GO term), and miscellaneous (no GO term).

Occasionally, ambiguous or conflicting annotations were observed. In these cases, protein BLASTp searches through the National Center for Biotechnology Information (NCBI) server were performed against the non-redundant protein database. If putative domains were detected within the amino acid sequence, a combination of the best hit (lowest E-value) and consensus among domain annotations were used to categorize the gene in question. If no putative domains were detected, the functional annotation was based on the best scoring protein hits in the database (E-value threshold of $1x10^{-25}$). The functional annotations of all chromosomal genes are provided in Data Set S6.

Development of iGD1575b.

For *in silico* analysis of redundancy in the *S. meliloti* genome, the previously published *S. meliloti* genome-scale metabolic model iGD1575 [3] was modified slightly. As indicated in Table S8, the biomass composition was updated to include 31 additional compounds at trace concentrations, including vitamins, coenzymes, and ions, in order to ensure the corresponding transport or biosynthetic pathways were essential. However, the original model iGD1575 was unable to produce vitamin B12 (calomide; cpd00166) and holo-carboxylate (cpd12847), as the addition of either compound into the biomass reaction prevented growth of the model. To rectify this, the reversibility of rxn00792_c0 was changed from 'false' to 'true', as this reaction must function in the reverse direction for production of holo-carboxylate, and the reactions rxn01609, rxn06864, and rxnBluB were added to the model. However, no new genes were included in the model. This updated model was termed iGD1575b and is available in SBML and MATLAB format in File S2.

Simulating the removal of pSymA and pSymB *in silico*.

Several modifications to iGD1575b were required in order to produce a viable model following the deletion of all pSymA and pSymB genes. As described previously [3], succinoglycan was removed from the biomass composition, 'gapfill' GPRs (gene-protein-reaction relationships) were added to the reactions 'rxn01675_c0', 'rxn01997_c0', 'rxn02000_c0', and 'rxn02003_c0' in order to allow the continued production of the full LPS molecule, as well as to 'rxn00416_c0' to allow asparagine biosynthesis. Additionally, 'gapfill'

GPRs were added to the reactions 'rxn03975_c0' and 'rxn03393_c0' so that removal of pSymA and pSymB did not prevent biosynthesis of vitamin B12 and ubiquinone-8, respectively. Finally, a glycerol export reaction via diffusion (rxnBLTPcpd00100b) was added to remove the glycerol build-up resulting from cardiolipin biosynthesis. The modified version of the model was termed iGD1575c, and is available in in SBML and MATLAB format in File S2. For simulating the removal of pSymA and pSymB in MATLAB, all pSymA and pSymB genes were deleted from the iGD1575b model using the *deleteModelGenes* function, followed by the removal of all constrained reactions using the *removeRxns* function.

Building the draft *R. leguminosarum* and *S. meliloti* metabolic models.

A draft, fully automated model containing no manual curation for *R. leguminosarum* bv. *viciae* 3841 was built using the KBase webserver (www.kbase.us). The Genbank file (GCA_000009265.1_ASM926v1_genomic.gbff) of the *R. leguminosarum* genome [4] was uploaded to KBase and re-annotated using the 'annotate microbial genome' function, maintaining the original locus tags. An automated metabolic model was then built using the 'build metabolic model' function, with gap-filling. This model included 1537 genes, 1647 reactions, and 1731 metabolites, and is available in in SBML and MATLAB format in File S2. The biomass composition was not modified from the default Gram negative biomass of KBase. All essential model genes were determined using the COBRA Toolbox in MATLAB with the *singleGeneDeletion* function and the MOMA protocol, with exchange reaction bounds set as provided in Table S7.

A draft, fully automated model containing no manual curation for *S. meliloti* Rm1021 was built as described above for *R. leguminosarum* but starting with the Genbank file (GCA_000006965.1_ASM696v1_genomic.gbff) for the *S. meliloti* genome [5]. The draft model included 1254 genes, 1698 reactions, and 1666 metabolites, and is available in in SBML and MATLAB format in File S2.

Building the *S. meliloti* core metabolic reconstruction, iGD726.

The iGD726 model was built from the ground-up using the existing genome-scale model as a reaction and GPR database, and with the Tn-seq data as a guide. The iGD726 model began as an empty SBML formatted file, and the first reaction that was added was a biomass reaction involving a single substrate (e.g., protein). The pathways (including central carbon metabolism) required to produce this biomass substrate were added reaction-by-reaction to the new reconstruction file. These reactions were taken from the original model, or were taken from other sources, primarily the Kyoto Encyclopedia of Genes and Genomes (KEGG) database [6], if an appropriate reaction was missing in the original model. Pathway reconstruction was guided by the metabolic maps of KEGG and the Tn-seq data; if multiple pathways could be used to generate the same compound, the essentiality data of the Tn-seq screen were used to inform the decision of which pathway to include. When the necessary pathways were built, the model was converted to COBRA format, and FBA simulations were run to test if biomass could be produced. If not, we attempted to identify gaps both manually, and with the *detectDeadEnds* function of the COBRA Toolbox. In cases where we remained unable to identify the missing reaction(s), all reactions from the original model were transferred to the new reconstruction, following which they were cumulatively deleted until only the essential reaction(s) remained. When it was understood why these reactions were essential, they were either retained in the new reconstruction, or replaced with an alternative pathway, guided by the Tn-seq data. When the

model was capable of producing biomass (based on FBA simulations), the next biomass component was added to the biomass reaction, and the process repeated. This iterative process continued until the model could produce all biomass reactions. As the original model is a full genome-scale metabolic reconstruction, encompassing core and accessory metabolism, not all reactions were transferred to iGD726; only those essential for biomass production or to accurately capture the Tn-seq data were included in iGD726.

Throughout the above process, the Tn-seq data were used to refine the gene-reaction associations. Each time a new reaction was added to the core reconstruction, the genes associated with the reaction were checked against the Tn-seq data, and a literature search for each associated gene was performed. The gene associations were then modified as necessary to ensure the model accurately captured the experimental data. For example, if a gene was experimentally determined to be essential (in the Tn-seq data or the literature), but the corresponding reaction for the gene was associated with multiple alternative genes, all genes but the essential gene were removed from the reaction. Similarly, for essential reactions associated with non-essential genes, either i) all genes associated with the reaction in the original model were added; ii) if only one gene was associated with the reaction in the original model, an Unknown was added to reflect the apparent redundancy in the genome; or iii) an alternative, redundant pathway was added. Where possible, Unknowns in the gene associations were replaced with genes whose gene product may catalyze the reaction; this was only done if there was some level of experimental support for doing so.

Core model reconstruction was performed providing sucrose as a carbon source during the FBA simulations. Once a functional model was prepared, it was expanded to include the necessary metabolic and transport reactions to also allow growth with glucose or succinate as the carbon source. Finally, when biomass could be produced using any of the three carbon sources, the list of model genes was compared with the list of 489 core growth promoting genes to identify genes not included in the model but experimentally determined to contribute to growth. When possible, missing genes and their corresponding reactions were added to the core model.

During the construction of the core model, we additionally updated the biomass composition. This included modifying the membrane lipid composition to include lipids with different sized fatty acids based on the ratio experimentally determined [7]; the original genome-scale model contained only one representative per each membrane lipid class. Additionally, essential vitamins, cofactors, and ions were added to the biomass composition at trace concentrations to ensure that their biosynthesis or transport was essential. The complete biomass composition is provided in Table S3.

The final model contained 726 genes, 681 reactions, and 703 metabolites, and is provided in SBML and MATLAB format in File S2, and as an Excel file in Data Set S5. The Excel file contains all necessary information for use as a *S. meliloti* metabolic resource, including the reaction name, the reaction equation using the real metabolite names, the associated genes/proteins, and references. Additionally, for each reaction, the putative orthologs of the associated genes in 10 related Rhizobiales species are included, allowing the model to provide useful information for each of these organisms.

SUPPLEMENTARY RESULTS

Validation of the between-strain differences in the Tn-seq data.

In an attempt to independently validate the results of the Tn-seq experiment, 16 genes showing strain-specific phenotypes (14 genes whose disruption appeared to result in a larger growth defect in the ΔpSymAB strain, and 2 genes whose disruption appeared to result in a larger growth defect in the wild-type strain) were mutated through single cross-over plasmid integration. As described in the Results section of this manuscript, the between-strain differences of ~ 75% of these genes were confirmed by the validation experiment. Even if only 75% of the between-strain differences in the Tn-seq data are real, this is still a remarkable number of between-strain differences. However, we expect that many of the differences between the Tn-seq and validation experiments are not false positives, but instead a result of differences in growth conditions. More specifically, the validation experiments involve isogenic cultures, whereas the Tn-seq experiment is based on competitive growth in which less than 0.01% of the initial cell population would contain the mutation of interest.

A possible example of the growth condition differences influencing the results is the data for *feuQ*. The *feuN*, *feuP*, and *feuQ* genes encode a system resulting expression of *ndvA* and *ndvB*, whose products are involved in periplasmic β-glucan biosynthesis [8,9]. In the Tn-seq data, all five of these related genes contained many fewer insertions in the ΔpSymAB background than in the wild-type background, and this was observed in both of the media. However, in the validation experiment, mutation of *feuQ* did not result in the expected phenotype (none of the other four genes were tested). Given that all five of these genes (encoded as three operons in two different chromosomal loci) are involved in a common process, and that all five have the same phenotype in the Tn-seq data (independent of medium), it is unlikely that these represent false positives. Instead, we interpret the difference between the Tn-seq and validation experiments as reflective of the difference in growth conditions of these two experiments, highlighting how a failure to validate a phenotype does not necessarily mean that the Tn-seq observed phenotype is a false positive.

In summary, it is expected that some of the genes identified as having between-strain differences in the Tn-seq data are false positives. However, many of those that could not be independently confirmed in the validation experiment many not represent false positives and instead reflect differences in experimental settings.

Comparison of pipelines for classification of gene fitness phenotypes.

The analyses presented in the manuscript are based on gene classifications performed using the clustering based approach described in the Materials and Methods. Subsequently, the TSAS pipeline [10] was run to verify that the clustering based approach did not result in the inclusion of excessive false positives. Here, these approaches are compared.

The main step of the clustering based approach involved assuming that the data are explained by multiple overlapping Guassian distributions (i.e., non-essential genes form one distribution, essential genes form an independent distribution, etc.). The number and shape of distributions that best explains the data were then found, and genes classified based on the probability that they belonged to each of the fitted distributions.

In the case of TSAS, the reads are reduced to the number of unique insertions; i.e., all reads mapping to the same location are reduced to a single insertion. The data are then assumed to belong to a single Binomial distribution, and the probability of finding the detected number of

insertions by chance is calculated. Different p-value corrections are then applied to correct for multiple testing. The key outputs of this approach are p-values indicating the probability that genes have fewer than expected insertions; it does not distinguish between essential genes and genes whose mutation result in a non-lethal growth defect.

To ensure that the various steps of clustering based approach did not result in the identification of an excessively large number of false positive essential genes, the outputs of these methods were compared. Of the 307 genes classified as essential in all four samples, 269 (88%) were considered to have fewer than expected insertions with the TSAS pipeline and a strict significance threshold (field wide error rate Bonferroni correction < 0.01). Most of the 38 genes not considered to have fewer than expected insertions were small genes with no insertions. However, inspection of their annotations revealed that most are expected to be essential, highlighted by the fact that this list includes 24 ribosomal proteins, and therefore their annotation as essential by the clustering pipeline is probably accurate. Additionally, if a somewhat less strict significance threshold is used (Benjamini-Hochberg adjusted p-value < 0.01), 300 (98%) of the 307 core essential genes are classified as having fewer than expected insertions. Given these results, we conclude that the clustering based approach did not result in an artificially high number of false positives, and that the global systems-level analyses reported in this study are unlikely to be significantly impacted by the choice of statistical analysis.

Results from both the clustering approach and the TSAS pipeline are provided in Data Set S1.

FILE S1 REFERENCES

1. Fraley C, Raftery AE, Murphy TB, Scrucca L. mclust Version 4 for R: Normal mixture modeling for model-based clustering, classification, and density estimation. Washington, USA: Department of Statistics, University of Washington; 2012.
2. Bodenhofer U, Kothmeier A, Hochreiter S. APCluster: an R package for affinity propagation clustering. Bioinformatics. 2011;27: 2463–2464. doi:10.1093/bioinformatics/btr406
3. diCenzo GC, Checcucci A, Bazzicalupo M, Mengoni A, Viti C, Dziewit L, et al. Metabolic modelling reveals the specialization of secondary replicons for niche adaptation in *Sinorhizobium meliloti*. Nat Commun. 2016;7: 12219. doi:10.1038/ncomms12219
4. Young JPW, Crossman LC, Johnston AW, Thomson NR, Ghazoui ZF, Hull KH, et al. The genome of *Rhizobium leguminosarum* has recognizable core and accessory components. Genome Biol. 2006;7: R34. doi:10.1186/gb-2006-7-4-r34
5. Galibert F, Finan TM, Long SR, Pühler A, Abola AP, Ampe F, et al. The composite genome of the legume symbiont *Sinorhizobium meliloti*. Science. 2001;293: 668–672. doi:10.1126/science.1060966
6. Kanehisa M, Sato Y, Kawashima M, Furumichi M, Tanabe M. KEGG as a reference resource for gene and protein annotation. Nucleic Acids Res. 2016;44: D457–D462. doi:10.1093/nar/gkv1070
7. Basconcillo LS, Zaheer R, Finan TM, McCarry BE. A shotgun lipidomics study of a putative lysophosphatidic acid acyl transferase (PlsC) in *Sinorhizobium meliloti*. J Chromatogr B. 2009;877: 2873–2882. doi:10.1016/j.jchromb.2009.05.014

8. Griffitts JS, Carlyon RE, Erickson JH, Moulton JL, Barnett MJ, Toman CJ, et al. A *Sinorhizobium meliloti* osmosensory two-component system required for cyclic glucan export and symbiosis. Mol Microbiol. 2008;69: 479–490. doi:10.1111/j.1365-2958.2008.06304.x

9. Carlyon RE, Ryther JL, VanYperen RD, Griffitts JS. FeuN, a novel modulator of two-component signalling identified in *Sinorhizobium meliloti*. Mol Microbiol. 2010;77: 170–182. doi:10.1111/j.1365-2958.2010.07198.x

10. Burger BT, Imam S, Scarborough MJ, Noguera DR, Donohue TJ. Combining genome-scale experimental and computational methods to identify essential genes in *Rhodobacter sphaeroides*. mSystems. 2017;2: e00015–17. doi:10.1128/mSystems.00015-17

DATA SET S5 REFERENCES

Abbas BA, Vineetha KE, Prasad CK, Vij N, Hassani R & Randhawa GS (2002) Symbiotic characteristics of cysteine and methionine auxotrophs of *Sinorhizobium meliloti*. *Indian J. Exp. Biol.* **40:** 1121–1130

Abola AP, Willits MG, Wang RC & Long SR (1999) Reduction of adenosine-5'-phosphosulfate instead of 3"-phosphoadenosine-5"-phosphosulfate in cysteine biosynthesis by *Rhizobium meliloti* and other members of the family *Rhizobiaceae*. *J. Bacteriol.* **181:** 5280–5287

Aneja P & Charles TC (1999) Poly-3-hydroxybutyrate degradation in *Rhizobium* (*Sinorhizobium*) *meliloti*: isolation and characterization of a gene encoding 3-hydroxybutyrate dehydrogenase. *J. Bacteriol.* **181:** 849–857

Aneja P, Dai M, Lacorre DA, Pillon B & Charles TC (2004) Heterologous complementation of the exopolysaccharide synthesis and carbon utilization phenotypes of *Sinorhizobium meliloti* Rm1021 polyhydroxyalkanoate synthesis mutants. *FEMS Microbio. Lett.* **239:** 277–283

Aneja P, Dziak R, Cai G-Q & Charles TC (2002) Identification of an acetoacetyl coenzyme A synthetase-dependent pathway for utilization of L-(+)-3-hydroxybutyrate in *Sinorhizobium meliloti*. *J. Bacteriol.* **184:** 1571–1577

Arias A, Cerveñansky C, Gardiol A & Martínez-Drets G (1979) Phosphoglucose isomerase mutant of *Rhizobium meliloti*. *J. Bacteriol.* **137:** 409–414

Baev N, Endre G, Petrovics G, Banfalvi Z & Kondorosi A (1991) Six nodulation genes of nod box locus 4 in *Rhizobium meliloti* are involved in nodulation signal production: *nodM* codes for D-glucosamine synthetase. *Mol Gen Genet* **228:** 113–124

Bardin S, Dan S, Østerås M & Finan TM (1996) A phosphate transport system is required for symbiotic nitrogen fixation by *Rhizobium meliloti*. *J. Bacteriol.* **178:** 4540–4547

Bardin SD, Voegele RT & Finan TM (1998) Phosphate assimilation in *Rhizobium* (*Sinorhizobium*) *meliloti*: identification of a *pit*-like gene. *J. Bacteriol.* **180:** 4219–4226

Barr M, East AK, Leonard M, Mauchline TH & Poole PS (2008) *In vivo* expression technology (IVET) selection of genes of *Rhizobium leguminosarum* biovar *viciae* A34 expressed in the rhizosphere. *FEMS Microbio. Lett.* **282:** 219–227

Barra L, Pica N, Gouffi K, Walker GC, Blanco C & Trautwetter A (2003) Glucose 6-phosphate dehydrogenase is required for sucrose and trehalose to be efficient osmoprotectants in *Sinorhizobium meliloti*. *FEMS Microbio. Lett.* **229:** 183–188

Barsomian GD, Urzainqui A, Lohman K & Walker GC (1992) Rhizobium meliloti mutants unable to synthesize anthranilate display a novel symbiotic phenotype. *J. Bacteriol.* **174:** 4416–4426

Basconcillo LS, Zaheer R, Finan TM & McCarry BE (2009a) Cyclopropane fatty acyl synthase in *Sinorhizobium meliloti*. *Microbiology* **155:** 373–385

Basconcillo LS, Zaheer R, Finan TM & McCarry BE (2009b) A shotgun lipidomics study of a putative lysophosphatidic acid acyl transferase (PlsC) in *Sinorhizobium meliloti*. *J. Chromatogr. B* **877:** 2873–2882

Becker A, Fraysse N & Sharypova L (2007) Recent advances in studies on structure and symbiosis-related function of rhizobial K-antigens and lipopolysaccharides. *Mol. Plant Microbe Interact.* **18:** 899–905

Becker A, Kleickmann A, Arnold W & Pühler A (1993a) Analysis of the *Rhizobium meliloti exoH/exoK/exoL* fragment: ExoK shows homology to excreted endo-β-1,3-1,4-glucanases and ExoH resembles membrane proteins. *Mol Gen Genet* **238:** 145–154

Becker A, Kleickmann A, Keller M, Arnold W & Puhler A (1993b) Identification and analysis of the *Rhizobium meliloti exoAMONP* genes involved in exopolysaccharide biosynthesis and mapping of promoters located on the *exoHKLAMONP* fragment. *Mol Gen Genet* **241:** 367–379

Becker A, Kleickmann A, Küster H, Keller M, Arnold W & Puhler A (1993c) Analysis of the *Rhizobium meliloti* genes *exoU*, *exoV*, *exoW*, *exoT*, and *exoI* involved in exopolysaccharide biosynthesis and nodule invasion: *exoU* and *exoW* probably encode glucosyltransferases. *Mol. Plant Microbe Interact.* **6:** 735–744

Becker A, Küster H, Niehaus K & Puhler A (1995) Extension of the Rhizobium meliloti succinoglycan biosynthesis gene cluster: identification of the exsA gene encoding an ABC transporter protein, and the exsB gene which probably codes for a regulator of succinoglycan biosynthesis. *Mol Gen Genet* **249:** 487–497

Becker A, Ruberg S, Küster H, Roxlau AA, Keller M, Ivashina T, Cheng HP, Walker GC & Puhler A (1997) The 32-kilobase *exp* gene cluster of *Rhizobium meliloti* directing the biosynthesis of galactoglucan: genetic organization and properties of the encoded gene products. *J. Bacteriol.* **179:** 1375–1384

Bielen MS (2015) Functional Characterization of the IpdA Genes in *Sinorhizoblum Meliloti*. **PhD Thesis.**

Billen D & Lichstein HC (1949) Aspartic acid decarboxylation by *Rhizobium trifolii*. *J. Bacteriol.* **57:** 267–268

Buendia AM, Enenkel B, Köplin R, Niehaus K, Arnold W & Puhler A (1991) The *Rhizobium meliloti exoZl exoB* fragment of megaplasmid 2: ExoB functions as a UDP-glucose 4-epimerase and ExoZ shows homology to NodX of *Rhizobium leguminosarum* biovar *viciae* strain TOM. *Mol. Microbiol.* **5:** 1519–1530

Cabanes D, Boistard P & Batut J (2000) Symbiotic induction of pyruvate dehydrogenase genes from *Sinorhizobium meliloti*. *Mol. Plant Microbe Interact.* **13:** 483–493

Campbell GRO, Reuhs BL & Walker GC (2002) Chronic intracellular infection of alfalfa nodules by *Sinorhizobium meliloti* requires correct lipopolysaccharide core. *Proc. Natl. Acad. Sci. U.S.A.* **99:** 3938–3943

Campbell GRO, Sharypova LA, Scheidle H, Jones KM, Niehaus K, Becker A & Walker GC (2003) Striking complexity of lipopolysaccharide defects in a collection of *Sinorhizobium meliloti* mutants. *J. Bacteriol.* **185:** 3853–3862

Campbell GRO, Taga ME, Mistry K, Lloret J, Anderson PJ, Roth JR & Walker GC (2006) *Sinorhizobium meliloti bluB* is necessary for production of 5,6-dimethylbenzimidazole, the lower ligand of B12. *Proc. Natl. Acad. Sci. U.S.A.* **103:** 4634–4639

Capela D, Filipe C, Bobik C, Batut J & Bruand C (2006) *Sinorhizobium meliloti* differentiation during symbiosis with alfalfa: a transcriptomic dissection. *Mol. Plant Microbe Interact.* **19:** 363–372

Carlyon RE, Ryther JL, VanYperen RD & Griffitts JS (2010) FeuN, a novel modulator of two-component signalling identified in *Sinorhizobium meliloti*. *Mol. Microbiol.* **77:** 170–182

Cerveñansky C & Arias A (1984) Glucose-6-phosphate dehydrogenase deficiency in pleiotropic carbohydrate-negative mutant strains of *Rhizobium meliloti*. *J. Bacteriol.* **160:** 1027–1030

Cheng J, Poduska B, Morton RA & Finan TM (2011) An ABC-type cobalt transport system is essential for growth of *Sinorhizobium meliloti* at trace metal concentrations. *J. Bacteriol.* **193:** 4405–4416

Cinege G, Kereszt A, Kertész S, Balogh G & Dusha I (2004) The roles of different regions of the CycH protein in c-type cytochrome biogenesis in *Sinorhizobium meliloti*. *Mol. Genet. Genomics* **271:** 171–179

Clover RH, Kieber J & Signer ER (1989) Lipopolysaccharide mutants of *Rhizobium meliloti* are not defective in symbiosis. *J. Bacteriol.* **171:** 3961–3967

Collavino M, Riccillo PM, Grasso DH, Crespi M & Aguilar M (2005) GuaB activity is required in Rhizobium tropici during the early stages of nodulation of determinate nodules but is dispensable for the Sinorhizobium meliloti-alfalfa symbiotic interaction. *Mol. Plant Microbe Interact.* **18:** 742–750

Cowie A, Cheng J, Sibley CD, Fong Y, Zaheer R, Patten CL, Morton RM, Golding GB & Finan TM (2006) An integrated approach to functional genomics: construction of a novel reporter gene fusion library for *Sinorhizobium meliloti*. *Appl. Environ. Microbiol.* **72:** 7156–7167

Crofts TS, Seth EC, Hazra AB & Taga ME (2013) Cobamide structure depends on both lower ligand availability and CobT substrate specificity. *Chem. Biol.* **20:** 1265–1274

Cuív PÓ, Keogh D, Clarke P & O'Connell M (2008) The *hmuUV* genes of *Sinorhizobium meliloti* 2011 encode the permease and ATPase components of an ABC transport system for the utilization of both haem and the hydroxamate siderophores, ferrichrome and ferrioxamine B. *Mol. Microbiol.* **70:** 1261–1273

Davies BW & Walker GC (2007a) Identification of novel *Sinorhizobium meliloti* mutants compromised for oxidative stress protection and symbiosis. *J. Bacteriol.* **189:** 2110–2113

Davies BW & Walker GC (2007b) Disruption of *sitA* compromises *Sinorhizobium meliloti* for manganese uptake required for protection against oxidative stress. *J. Bacteriol.* **189:** 2101–2109

De Bruijn FJ, Rossbach S, Schneider M, Ratet P, Messmer S, Szeto WW, Ausubel FM & Schell J (1989) *Rhizobium meliloti* 1021 has three differentially regulated loci involved in glutamine biosynthesis, none of which is essential for symbiotic nitrogen fixation. *J. Bacteriol.* **171:** 1673–1682

de Rudder KE, Lopéz-Lara IM & Geiger O (2000) Inactivation of the gene for phospholipid N-methyltransferase in *Sinorhizobium meliloti*: phosphatidylcholine is required for normal growth. *Mol. Microbiol.* **37:** 763–772

diCenzo G, Milunovic B, Cheng J & Finan TM (2013) The $tRNA^{arg}$ gene and *engA* are essential genes on the 1.7-mb pSymB megaplasmid of *Sinorhizobium meliloti* and were translocated together from the chromosome in an ancestral strain. *J. Bacteriol.* **195:** 202–212

diCenzo GC & Finan TM (2015) Genetic redundancy is prevalent within the 6.7 Mb *Sinorhizobium meliloti* genome. *Mol. Genet. Genomics* **290:** 1345–1356

diCenzo GC, Checcucci A, Bazzicalupo M, Mengoni A, Viti C, Dziewit L, Finan TM, Galardini M & Fondi M (2016) Metabolic modelling reveals the specialization of secondary replicons for niche adaptation in *Sinorhizobium meliloti*. *Nat. Commun.* **7:** 12219

diCenzo GC, MacLean AM, Milunovic B, Golding GB & Finan TM (2014) Examination of prokaryotic multipartite genome evolution through experimental genome reduction. *PLOS Genet.* **10:** e1004742

diCenzo GC, Sharthiya H, Nanda A, Zamani M & Finan TM (2017) PhoU allows rapid adaptation to high phosphate concentrations by modulating PstSCAB transport rate in *Sinorhizobium meliloti*. *J. Bacteriol.***:** JB.00143–17

diCenzo GC, Zamani M, Cowie A & Finan TM (2015) Proline auxotrophy in *Sinorhizobium meliloti* results in a plant-specific symbiotic phenotype. *Microbiology* **161:** 2341–2351

Díaz R, Vargas-Lagunas C, Villalobos MA, Peralta H, Mora Y, Encarnación S, Girard L & Mora J (2011) *argC* orthologs from *Rhizobiales* show diverse profiles of transcriptional efficiency and functionality in *Sinorhizobium meliloti*. *J. Bacteriol.* **193:** 460–472

Domínguez-Ferreras A, Muñoz S, Olivares J, Soto MJ & Sanjuán J (2009) Role of potassium uptake systems in *Sinorhizobium meliloti* osmoadaptation and symbiotic performance. *J. Bacteriol.* **191:** 2133–2143

Driscoll B & Finan TM (1993) NAD^+-dependent malic enzyme of *Rhizobium meliloti* is required for symbiotic nitrogen fixation. *Mol. Microbiol.* **7:** 865–873

Driscoll B & Finan TM (1996) $NADP^+$-dependent malic enzyme of *Rhizobium meliloti*. *J. Bacteriol.* **178:** 2224–2231

Dunn MF (2015) Key roles of microsymbiont amino acid metabolism in rhizobia-legume interactions. *Critical Reviews in Microbiology* **41:** 411–451

Dunn MF, Araíza G & Finan TM (2001) Cloning and characterization of the pyruvate carboxylase from *Sinorhizobium meliloti* Rm1021. *Arch Microbiol* **176:** 355–363

Dymov SL, Meek DJJ, Steven B & Driscoll B (2004) Insertion of transposon Tn*5*tac1 in the *Sinorhizobium meliloti* malate dehydrogenase (*mdh*) gene results in conditional polar effects on downstream TCA cycle genes. *Mol. Plant Microbe Interact.* **17:** 1318–1327

Engelke T, Jording D, Kapp D & Puhler A (1989) Identification and sequence analysis of the *Rhizobium meliloti dctA* gene encoding the C_4-dicarboxylate carrier. *J. Bacteriol.* **171:** 5551–5560

Entcheva P, Phillips DA & Streit WR (2002) Functional analysis of *Sinorhizobium meliloti* genes involved in biotin synthesis and transport. *Appl. Environ. Microbiol.* **68:** 2843–2848

Feng Y & Cronan JE (2009) *Escherichia coli* unsaturated fatty acid synthesis: complex transcription of the *fabA* gene and *in vivo* identification of the essential reaction catalyzed by FabB. *J. Biol. Chem.* **284:** 29526–29535

Ferguson GP, Datta A, Carlson RW & Walker GC (2005) Importance of unusually modified lipid A in *Sinorhizobium* stress resistance and legume symbiosis. *Mol. Microbiol.* **56:** 68–80

Finan TM, Hirsch AM, Leigh JA, Johansen E, Kuldau GA, Deegan S, Walker GC & Signer ER (1985) Symbiotic mutants of *Rhizobium meliloti* that uncouple plant from bacterial differentiation. *Cell* **40:** 869–877

Finan TM, Kunkel B, De Vos GF & Signer ER (1986) Second symbiotic megaplasmid in *Rhizobium meliloti* carrying exopolysaccharide and thiamine synthesis genes. *J. Bacteriol.* **167:** 66–72

Finan TM, Oresnik IJ & Bottacin A (1988) Mutants of *Rhizobium meliloti* defective in succinate metabolism. *J. Bacteriol.* **170:** 3396–3403

Frage B, Döhlemann J, Robledo M, Lucena D, Sobetzko P, Graumann PL & Becker A (2016) Spatiotemporal choreography of chromosome and megaplasmids in the *Sinorhizobium meliloti* cell cycle. *Mol. Microbiol.* **100:** 808–823

Gagnon Y, Lacoste L, Champagne N & Lapointe J (1996) Widespread use of the glu-tRNAGln transamidation pathway among bacteria. A member of the alpha purple bacteria lacks glutaminyl-tRNA synthetase. *J. Biol. Chem.* **271:** 14856–14863

Gao J-L, Weissenmayer B, Taylor AM, Thomas-Oates J, López-Lara IM & Geiger O (2004) Identification of a gene required for the formation of lyso-ornithine lipid, an intermediate in the biosynthesis of ornithine-containing lipids. *Mol. Microbiol.* **53:** 1757–1770

Gardiol A, Arias A, Cerveñansky C & Martínez-Drets G (1982) Succinate dehydrogenase mutant of *Rhizobium meliloti*. *J. Bacteriol.* **151:** 1621–1623

Garwin JL, Klages AL & Cronan JE (1980) Beta-ketoacyl-acyl carrier protein synthase II of *Escherichia coli*. Evidence for function in the thermal regulation of fatty acid synthesis. *J. Biol. Chem.* **255:** 3263–3265

Gay NJ (1984) Construction and characterization of an *Escherichia coli* strain with a *uncI* mutation. *J. Bacteriol.* **158:** 820–825

Geddes BA & Oresnik IJ (2014) Physiology, genetics, and biochemistry of carbon metabolism in the alphaproteobacterium *Sinorhizobium meliloti*. *Can. J. Microbiol.* **60:** 491–507

Geiger O, López-Lara IM & Sohlenkamp C (2013) Phosphatidylcholine biosynthesis and function in bacteria. *Biochim. Biophys. Acta, Mol. Cell. Biol. Lipids* **1831:** 503–513

Glenn SA, Gurich N, Feeney MA & González JE (2007) The ExpR/Sin quorum-sensing system controls succinoglycan production in *Sinorhizobium meliloti*. *J. Bacteriol.* **189:** 7077–7088

Glucksmann MA, Reuber TL & Walker GC (1993) Genes needed for the modification, polymerization, export, and processing of succinoglycan by *Rhizobium meliloti*: a model for succinoglycan biosynthesis. *J. Bacteriol.* **175:** 7045–7055

Gonzalez JE, Semino CE, Wang LX, Castellano-Torres LE & Walker GC (1998) Biosynthetic control of molecular weight in the polymerization of the octasaccharide subunits of succinoglycan, a symbiotically important exopolysaccharide of *Rhizobium meliloti*. *Proc. Natl. Acad. Sci. U.S.A.* **95:** 13477–13482

Griffitts JS & Long SR (2008) A symbiotic mutant of *Sinorhizobium meliloti* reveals a novel genetic pathway involving succinoglycan biosynthetic functions. *Mol. Microbiol.* **67:** 1292–1306

Griffitts JS, Carlyon RE, Erickson JH, Moulton JL, Barnett MJ, Toman CJ & Long SR (2008) A *Sinorhizobium meliloti* osmosensory two-component system required for cyclic glucan export and symbiosis. *Mol. Microbiol.* **69:** 479–490

Gu X, Lee SG & Bar-Peled M (2010) Biosynthesis of UDP-xylose and UDP-arabinose in *Sinorhizobium meliloti* 1021: first characterization of a bacterial UDP-xylose synthase, and UDP-xylose 4-epimerase. *Microbiology* **157:** 260–269

Guillén-Navarro K, Araíza G, García-de-los-Santos A, Mora Y & Dunn MF (2005) The *Rhizobium etli bioMNY* operon is involved in biotin transport. *FEMS Microbio. Lett.* **250:** 209–219

Haag AF, Wehmeier S, Muszyński A, Kerscher B, Fletcher V, Berry SH, Hold GL, Carlson RW & Ferguson GP (2011) Biochemical characterization of *Sinorhizobium meliloti* mutants

reveals gene products involved in the biosynthesis of the unusual lipid A very long-chain fatty acid. *J. Biol. Chem.* **286:** 17455–17466

Harrison J, Jamet A, Muglia CI, Van de Sype G, Aguilar OM, Puppo A & Frendo P (2005) Glutathione plays a fundamental role in growth and symbiotic capacity of *Sinorhizobium meliloti*. *J. Bacteriol.* **187:** 168–174

Hawkins JP, Geddes BA & Oresnik IJ (2017a) Common Dyes Used to Determine Bacterial Polysaccharides on Agar Are Affected by Medium Acidification. *Can. J. Microbiol.* **63:** 559–562

Hawkins JP, Ordonez PA & Oresnik IJ (2017b) Characterization of mutants that affect the non-oxidative pentose phosphate pathway in *Sinorhizobium meliloti*. *J. Bacteriol.* **[epub ahead of print]:** JB.00436–17

Hazra AB, Han Y, Chatterjee A, Zhang Y, Lai R-Y, Ealick SE & Begley TP (2011) A missing enzyme in thiamin thiazole biosynthesis: identification of TenI as a thiazole tautomerase. *J. Am. Chem. Soc.* **133:** 9311–9319

Heath RJ & Rock CO (1996) Roles of the FabA and FabZ beta-hydroxyacyl-acyl carrier protein dehydratases in *Escherichia coli* fatty acid biosynthesis. *J. Biol. Chem.* **271:** 27795–27801

Hernández VM, Girard L, Hernández-Lucas I, Vázquez A, Ortíz-Ortíz C, Díaz R & Dunn MF (2015) Genetic and biochemical characterization of arginine biosynthesis in *Sinorhizobium meliloti* 1021. *Microbiology* **161:** 1671–1682

Hood G, Karunakaran R, Downie JA & Poole P (2015) MgtE From *Rhizobium leguminosarum* is a Mg^{2+} channel essential for growth at low pH and N_2 fixation on specific plants. *Mol. Plant Microbe Interact.* **28:** 1281–1287

Jensen JB, Peters NK & Bhuvaneswari TV (2002) Redundancy in periplasmic binding protein-dependent transport systems for trehalose, sucrose, and maltose in *Sinorhizobium meliloti*. *J. Bacteriol.* **184:** 2978–2986

Jones KM, Kobayashi H, Davies BW, Taga ME & Walker GC (2007) How rhizobial symbionts invade plants: the *Sinorhizobium–Medicago* model. *Nat Rev Microbiol* **5:** 619–633

Kanipes MI, Kalb SR, Cotter RJ, Hozbor DF, Lagares A & Raetz CRH (2003) Relaxed sugar donor selectivity of a *Sinorhizobium meliloti* ortholog of the *Rhizobium leguminosarum* mannosyl transferase LpcC. Role of the lipopolysaccharide core in symbiosis of *Rhizobiaceae* with plants. *J. Biol. Chem.* **278:** 16365–16371

Keating DH, Willits MG & Long SR (2002) A *Sinorhizobium meliloti* lipopolysaccharide mutant altered in cell surface sulfation. *J. Bacteriol.* **184:** 6681–6689

Kereszt A, Kiss E, Reuhs BL, Carlson RW, Kondorosi A & Putnoky P (1998) Novel *rkp* gene clusters of *Sinorhizobium meliloti* involved in capsular polysaccharide production and invasion of the symbiotic nodule: the *rkpK* gene encodes a UDP-glucose dehydrogenase. *J. Bacteriol.* **180:** 5426–5431

Kereszt A, Slaska-Kiss K, Putnoky P, Banfalvi Z & Kondorosi A (1995) The *cycHJKL* genes of *Rhizobium meliloti* involved in cytochrome c biogenesis are required for 'respiratory' nitrate reduction *ex planta* and for nitrogen fixation during symbiosis. *Mol Gen Genet* **247:** 39–47

Kerppola TK & Kahn ML (1988a) Genetic analysis of carbamoylphosphate synthesis in *Rhizobium meliloti* 104A14. *J. Gen. Microbiol.* **134:** 921–929

Kerppola TK & Kahn ML (1988b) Symbiotic phenotypes of auxotrophic mutants of *Rhizobium*

meliloti 104A14. *Microbiology* **134:** 913–919

Koziol U, Hannibal L, Rodríguez MC, Fabiano E, Kahn ML & Noya F (2009) Deletion of citrate synthase restores growth of *Sinorhizobium meliloti* 1021 aconitase mutants. *J. Bacteriol.* **191:** 7581–7586

Lagares A, Caetano-Anolles G, Niehaus K, Lorenzen J, Ljunggren HD, Puhler A & Favelukes G (1992) A *Rhizobium meliloti* lipopolysaccharide mutant altered in competitiveness for nodulation of alfalfa. *J. Bacteriol.* **174:** 5941–5952

Lagares A, Hozbor DF, Niehaus K, Otero AJ, Lorenzen J, Arnold W & Puhler A (2001) Genetic characterization of a *Sinorhizobium meliloti* chromosomal region in lipopolysaccharide biosynthesis. *J. Bacteriol.* **183:** 1248–1258

las Nieves Peltzer de M, Roques N, Poinsot V, Aguilar OM, Batut J & Capela D (2008) Auxotrophy accounts for nodulation defect of most *Sinorhizobium meliloti* mutants in the branched-chain amino acid biosynthesis pathway. *Mol. Plant Microbe Interact.* **21:** 1232–1241

Leigh JA, Signer ER & Walker GC (1985) Exopolysaccharide-deficient mutants of *Rhizobium meliloti* that form ineffective nodules. *Proc. Natl. Acad. Sci. U.S.A.* **82:** 6231–6235

Lewis TA, Gonzalez R & Botsford JL (1990) *Rhizobium meliloti* glutamate synthase: cloning and initial characterization of the *glt* locus. *J. Bacteriol.* **172:** 2413–2420

Liu K, Myers AR, Pisithkul T, Claas KR, Satyshur KA, Amador-Noguez D, Keck JL & Wang JD (2015) Molecular mechanism and evolution of guanylate kinase regulation by (p)ppGpp. *Mol. Cell* **57:** 735–749

Long S, Reed JW, Himawan J & Walker GC (1988) Genetic analysis of a cluster of genes required for synthesis of the calcofluor-binding exopolysaccharide of *Rhizobium meliloti*. *J. Bacteriol.* **170:** 4239–4248

Lopéz-Lara IM & Geiger O (2000) Expression and purification of four different rhizobial acyl carrier proteins. *Microbiology* **146:** 839–849

López-Lara IM, Sohlenkamp C & Geiger O (2003) Membrane lipids in plant-associated bacteria: their biosyntheses and possible functions. *Mol. Plant Microbe Interact.* **16:** 567–579

Mao Y-H, Li F, Ma J-C, Hu Z & Wang H-H (2016) *Sinorhizobium meliloti* Functionally Replaces 3-Oxoacyl-Acyl Carrier Protein Reductase (FabG) by Overexpressing NodG During Fatty Acid Synthesis. *Mol. Plant Microbe Interact.* **29:** 458–467

Margolin W, Bramhill D & Long SR (1995) The *dnaA* gene of *Rhizobium meliloti* lies within an unusual gene arrangement. *J. Bacteriol.* **177:** 2892–2900

Mauchline TH, Fowler JE, East AK, Sartor AL, Zaheer R, Hosie AHF, Poole PS & Finan TM (2006) Mapping the *Sinorhizobium meliloti* 1021 solute-binding protein-dependent transportome. *Proc. Natl. Acad. Sci. U.S.A.* **103:** 17933–17938

McDermott TR & Kahn ML (1992) Cloning and mutagenesis of the *Rhizobium meliloti* isocitrate dehydrogenase gene. *J. Bacteriol.* **174:** 4790–4797

Medina C, Crespo-Rivas JC, Moreno J, Espuny MR & Cubo MT (2009) Mutation in the *cobO* gene generates auxotrophy for cobalamin and methionine and impairs the symbiotic properties of *Sinorhizobium fredii* HH103 with soybean and other legumes. *Arch Microbiol* **191:** 11–21

Merkamm M, Chassagnole C, Lindley ND & Guyonvarch A (2003) Ketopantoate reductase

activity is only encoded by *ilvC* in *Corynebacterium glutamicum*. *J. Biotechnol.* **104:** 253–260

Metzger LE & Raetz CRH (2010) An alternative route for UDP-diacylglucosamine hydrolysis in bacterial lipid A biosynthesis. *Biochemistry* **49:** 6715–6726

Miller KJ, McKinstry MW, Hunt WP & Nixon BT (1992) Identification of the diacylglycerol kinase structural gene of *Rhizobium meliloti* 1021. *Mol. Plant Microbe Interact.* **5:** 363–371

Miller-Williams M, Loewen PC & Oresnik IJ (2006) Isolation of salt-sensitive mutants of *Sinorhizobium meliloti* strain Rm1021. *Microbiology* **152:** 2049–2059

Mortimer MW, McDermott TR, York GM, Walker GC & Kahn ML (1999) Citrate synthase mutants of *Sinorhizobium meliloti* are ineffective and have altered cell surface polysaccharides. *J. Bacteriol.* **181:** 7608–7613

Mulley G, Lopez-Gomez M, Zhang Y, Terpolilli J, Prell J, Finan T & Poole P (2010) Pyruvate is synthesized by two pathways in pea bacteroids with different efficiencies for nitrogen fixation. *J. Bacteriol.* **192:** 4944–4953

Müller P, Keller M, Weng WM, Quandt J, Arnold W & Puhler A (1993) Genetic analysis of the *Rhizobium meliloti exoYFQ* operon: ExoY is homologous to sugar transferases and ExoQ represents a transmembrane protein. *Mol. Plant Microbe Interact.* **6:** 55–65

Niehaus K, Lagares A & Puhler A (1998) A *Sinorhizobium meliloti* Lipopolysaccharide Mutant Induces Effective Nodules on the Host Plant *Medicago sativa* (Alfalfa) but Fails to Establish a Symbiosis with *Medicago truncatula*. *Mol. Plant Microbe Interact.* **11:** 906–914

Nielsen BL & Brown LR (1985) Purification and subunit characterization of *Rhizobium meliloti* RNA polymerase. *J. Bacteriol.* **162:** 645–650

Nisa-Martínez R, Molina-Sánchez MD & Toro N (2016) Host Factors Influencing the Retrohoming Pathway of Group II Intron RmInt1, Which Has an Intron-Encoded Protein Naturally Devoid of Endonuclease Activity. *PLOS One* **11:** e0162275

Nogales J, Domínguez-Ferreras A, Amaya-Gomez CV, van Dillewijn P, Cuellar V, Sanjuán J, Olivares J & Soto MJ (2010) Transcriptome profiling of a *Sinorhizobium meliloti fadD* mutant reveals the role of rhizobactin 1021 biosynthesis and regulation genes in the control of swarming. *BMC Genomics* **11:** 157

O'flaherty S, Moënne Loccoz Y, Boesten B, Higgins P, Dowling DN, Condon S & O'Gara F (1995) Greenhouse and Field Evaluations of an Autoselective System Based on an Essential Thymidylate Synthase Gene for Improved Maintenance of Plasmid Vectors in Modified *Rhizobium meliloti*. *Appl. Environ. Microbiol.* **61:** 4051–4056

Peck MC, Gaal T, Fisher RF, Gourse RL & Long SR (2002) The RNA polymerase alpha subunit from *Sinorhizobium meliloti* can assemble with RNA polymerase subunits from *Escherichia coli* and function in basal and activated transcription both *in vivo* and *in vitro*. *J. Bacteriol.* **184:** 3808–3814

Pelchat M, Gagnon Y, Laberge S & Lapointe J (1999) Co-transcription of *Rhizobium meliloti* lysyl-tRNA synthetase and glutamyl-tRNA synthetase genes. *FEBS Lett.* **449:** 23–27

Pickering BS & Oresnik IJ (2008) Formate-dependent autotrophic growth in *Sinorhizobium meliloti*. *J. Bacteriol.* **190:** 6409–6418

Platero RA, Jaureguy M, Battistoni FJ & Fabiano ER (2003) Mutations in *sit B* and *sit D* genes affect manganese-growth requirements in *Sinorhizobium meliloti*. *FEMS Microbio. Lett.*

218: 65–70

Platt MW, Miller KJ, Lane WS & Kennedy EP (1990) Isolation and characterization of the constitutive acyl carrier protein from *Rhizobium meliloti*. *J. Bacteriol.* **172:** 5440–5444

Pobigaylo N, Szymczak S, Nattkemper TW & Becker A (2008) Identification of genes relevant to symbiosis and competitiveness in *Sinorhizobium meliloti* using signature-tagged mutants. *Mol. Plant Microbe Interact.* **21:** 219–231

Pobigaylo N, Wetter D, Szymczak S, Schiller U, Kurtz S, Meyer F, Nattkemper TW & Becker A (2006) Construction of a large signature-tagged mini-Tn*5* transposon library and its application to mutagenesis of *Sinorhizobium meliloti*. *Appl. Environ. Microbiol.* **72:** 4329–4337

Poysti NJ & Oresnik IJ (2007) Characterization of *Sinorhizobium meliloti* triose phosphate isomerase genes. *J. Bacteriol.* **189:** 3445–3451

Ramírez-Trujillo JA, Dunn MF, Suárez-Rodríguez R & Hernández-Lucas I (2016) The *Sinorhizobium meliloti* glyoxylate cycle enzyme isocitrate lyase (AceA) is required for the utilization of poly-β-hydroxybutyrate during carbon starvation. *Ann Microbiol* **66:** 921–924

Ramírez-Trujillo JA, Encarnacion S, Salazar E, de los Santos AG, Dunn MF, Emerich DW, Calva E & Hernández-Lucas I (2007) Functional characterization of the *Sinorhizobium meliloti* acetate metabolism genes *aceA*, *SMc00767*, and *glcB*. *J. Bacteriol.* **189:** 5875–5884

Rastogi VK & Watson RJ (1991) Aspartate aminotransferase activity is required for aspartate catabolism and symbiotic nitrogen fixation in *Rhizobium meliloti*. *J. Bacteriol.* **173:** 2879–2887

Reuber TL & Walker GC (1993) The acetyl substituent of succinoglycan is not necessary for alfalfa nodule invasion by *Rhizobium meliloti* Rm1021. *J. Bacteriol.* **175:** 3653–3655

Ross P, O'Gara F & Condon S (1990) Thymidylate synthase gene from *Lactococcus lactis* as a genetic marker: an alternative to antibiotic resistance genes. *Appl. Environ. Microbiol.* **56:** 2164–2169

Rudnick PA, Arcondéguy T, Kennedy CK & Kahn D (2001) *glnD* and *mviN* are genes of an essential operon in *Sinorhizobium meliloti*. *J. Bacteriol.* **183:** 2682–2685

Rushing BG & Long SR (1995) Cloning and characterization of the *sigA* gene encoding the major sigma subunit of *Rhizobium meliloti*. *J. Bacteriol.* **177:** 6952–6957

Sandoval-Calderón M, Geiger O, Guan Z, Barona-Gómez F & Sohlenkamp C (2009) A eukaryote-like cardiolipin synthase is present in *Streptomyces coelicolor* and in most actinobacteria. *J. Biol. Chem.* **284:** 17383–17390

Sanjuán-Pinilla JM, Muñoz S, Nogales J, Olivares J & Sanjuán J (2002) Involvement of the *Sinorhizobium meliloti leuA* gene in activation of nodulation genes by NodD1 and luteolin. *Arch Microbiol* **178:** 36–44

Schmidt M, Arnold W, Niemann A, Kleickmann A & Puhler A (1992) The *Rhizobium meliloti pmi* gene encodes a new type of phosphomannose isomerase. *Gene* **122:** 35–43

Seo M-J, Im E-M, Hur J-H, Nam J-Y, Hyun C-G, Pyun Y-R & Kim S-O (2006) Production of coenzyme Q10 by recombinant *E. coli* harboring the decaprenyl diphosphate synthase gene from *Sinorhizobium meliloti*. *Journal of microbiology and biotechnology* **16:** 933–938

Sharypova LA, Niehaus K, Scheidle H, Holst O & Becker A (2003) *Sinorhizobium meliloti acpXL* mutant lacks the C28 hydroxylated fatty acid moiety of lipid A and does not express a

slow migrating form of lipopolysaccharide. *J. Biol. Chem.* **278:** 12946–12954

Silvia Ardissone, Pierre Frendo, Enzo Laurenti, Walter Jantschko, Christian Obinger, Alain Puppo ARosa Pia Ferrari (2004) Purification and physical-chemical characterization of the three hydroperoxidases from the symbiotic bacterium *Sinorhizobium meliloti*. *Biochemistry* **43:** 12692–12699

Skorupska A, Janczarek M, Marczak M, Mazur A & Król J (2006) Rhizobial exopolysaccharides: genetic control and symbiotic functions. *Microb Cell Fact* **5:** 7

Sohlenkamp C, de Rudder KEE & Geiger O (2004) Phosphatidylethanolamine is not essential for growth of *Sinorhizobium meliloti* on complex culture media. *J. Bacteriol.* **186:** 1667–1677

Soto MJ, Sanjuán J & Olivares J (2001) The disruption of a gene encoding a putative arylesterase impairs pyruvate dehydrogenase complex activity and nitrogen fixation in *Sinorhizobium meliloti*. *Mol. Plant Microbe Interact.* **14:** 811–815

Taga ME & Walker GC (2010) *Sinorhizobium meliloti* requires a cobalamin-dependent ribonucleotide reductase for symbiosis with its plant host. *Mol. Plant Microbe Interact.* **23:** 1643–1654

Taga ME, Larsen NA, Howard-Jones AR, Walsh CT & Walker GC (2007) BluB cannibalizes flavin to form the lower ligand of vitamin B12. *Nature* **446:** 449–453

Tazoe M, Ichikawa K & Hoshino T (2000) Biosynthesis of vitamin B(6) in *Rhizobium*. *J. Biol. Chem.* **275:** 11300–11305

Tazoe M, Ichikawa K & Hoshino T (2006) Flavin adenine dinucleotide-dependent 4-phospho-D-erythronate dehydrogenase is responsible for the 4-phosphohydroxy-L-threonine pathway in vitamin B6 biosynthesis in *Sinorhizobium meliloti*. *J. Bacteriol.* **188:** 4635–4645

Trainer MA (2009) Carbon Metabolism and Desiccation Tolerance in the Nitrogen-Fixing Rhizobia *Bradyrhizobium japonicum* and *Sinorhizobium meliloti*. **PhD Thesis.**

Vahling-Armstrong CM, Zhou H, Benyon L, Morgan JK & Duan Y (2012) Two plant bacteria, *S. meliloti* and *Ca*. Liberibacter asiaticus, share functional *znuABC* homologues that encode for a high affinity zinc uptake system. *PLOS One* **7:** e37340

Vences-Guzmán MA, Geiger O & Sohlenkamp C (2008) *Sinorhizobium meliloti* mutants deficient in phosphatidylserine decarboxylase accumulate phosphatidylserine and are strongly affected during symbiosis with alfalfa. *J. Bacteriol.* **190:** 6846–6856

Vineetha KE, Vij N, Prasad CK, Hassani R & Randhawa GS (2001) Ultrastructural studies on nodules induced by pyrimidine auxotrophs of *Sinorhizobium meliloti*. *Indian J. Exp. Biol.* **39:** 371–377

Voegele RT, Bardin S & Finan TM (1997) Characterization of the *Rhizobium* (*Sinorhizobium*) *meliloti* high- and low-affinity phosphate uptake systems. *J. Bacteriol.* **179:** 7226–7232

Wang C, Saldanha M, Sheng X, Shelswell KJ, Walsh KT, Sobral BWS & Charles TC (2007a) Roles of poly-3-hydroxybutyrate (PHB) and glycogen in symbiosis of *Sinorhizobium meliloti* with *Medicago* sp. *Microbiology* **153:** 388–398

Wang C, Sheng X, Equi RC, Trainer MA, Charles TC & Sobral BWS (2007b) Influence of the poly-3-hydroxybutyrate (PHB) granule-associated proteins (PhaP1 and PhaP2) on PHB accumulation and symbiotic nitrogen fixation in *Sinorhizobium meliloti* Rm1021. *J. Bacteriol.* **189:** 9050–9056

Watson RJ & Rastogi VK (1993) Cloning and nucleotide sequencing of *Rhizobium meliloti* aminotransferase genes: an aspartate aminotransferase required for symbiotic nitrogen fixation is atypical. *J. Bacteriol.* **175:** 1919–1928

Weissenmayer B, Gao J-L, López-Lara IM & Geiger O (2002) Identification of a gene required for the biosynthesis of ornithine-derived lipids. *Mol. Microbiol.* **45:** 721–733

Weissenmayer B, Geiger O & Benning C (2000) Disruption of a gene essential for sulfoquinovosyldiacylglycerol biosynthesis in *Sinorhizobium meliloti* has no detectable effect on root nodule symbiosis. *Mol. Plant Microbe Interact.* **13:** 666–672

Wells DH & Long SR (2003) Mutations in rpoBC suppress the defects of a *Sinorhizobium meliloti relA* mutant. *J. Bacteriol.* **185:** 5602–5610

Willis LB & Walker GC (1998) The *phbC* (poly-beta-hydroxybutyrate synthase) gene of *Rhizobium* (*Sinorhizobium*) *meliloti* and characterization of *phbC* mutants. *Can. J. Microbiol.* **44:** 554–564

Willis LB & Walker GC (1999) A novel *Sinorhizobium meliloti* operon encodes an α-glucosidase and a periplasmic-binding-protein-dependent transport system for α-glucosides. *J. Bacteriol.* **181:** 4176–4184

Yang G, Bhuvaneswari TV, Joseph CM, King MD & Phillips DA (2002) Roles for riboflavin in the *Sinorhizobium*-alfalfa association. *Mol. Plant Microbe Interact.* **15:** 456–462

York GM & Walker GC (1997) The *Rhizobium meliloti exoK* gene and *prsD*/*prsE*/*exsH* genes are components of independent degradative pathways which contribute to production of low-molecular-weight succinoglycan. *Mol. Microbiol.* **25:** 117–134

York GM & Walker GC (1998) The *Rhizobium meliloti* ExoK and ExsH glycanases specifically depolymerize nascent succinoglycan chains. *Proc. Natl. Acad. Sci. U.S.A.* **95:** 4912–4917

Yuan Z-C, Zaheer R & Finan TM (2006) Regulation and properties of PstSCAB, a high-affinity, high-velocity phosphate transport system of *Sinorhizobium meliloti*. *J. Bacteriol.* **188:** 1089–1102

Yurgel SN & Kahn ML (2005) *Sinorhizobium meliloti dctA* mutants with partial ability to transport dicarboxylic acids. *J. Bacteriol.* **187:** 1161–1172

Yurgel SN, Berrocal J & Wilson C (2007) Pleiotropic effects of mutations that alter the *Sinorhizobium meliloti* cytochrome c respiratory system. *Microbiology*

Yurgel SN, Rice J, Domreis E, Lynch J, Sa N, Qamar Z, Rajamani S, Gao M, Roje S & Bauer WD (2014) *Sinorhizobium meliloti* flavin secretion and bacteria-host interaction: role of the bifunctional RibBA protein. *Mol. Plant Microbe Interact.* **27:** 437–445

Østerås M, Driscoll BT & Finan TM (1995) Molecular and expression analysis of the *Rhizobium meliloti* phosphoenolpyruvate carboxykinase (*pckA*) gene. *J. Bacteriol.* **177:** 1452–1460
