# Supplementary material for: Robustness encoded across essential and accessory replicons of the ecologically versatile bacterium Sinorhizobium meliloti
Source: PLoS Genet. 2018 Apr 19;14(4):e1007357. doi: 10.1371/journal.pgen.1007357 (PMC5929573; doi:10.1371/journal.pgen.1007357)
Supplement: S1 Table — (PDF) [file pgen.1007357.s004.pdf]

Table S1. Tn-seq library coverage.

Replicate	Chromosomal read count *	Chromosomal unique insertion sites †	Median unique insertions per gene
Wild-type - rich - 1	8077681	186102	38
Wild-type - rich - 2	6606632	189284	39
Wild-type - defined - 1	4808590	177099	37
Wild-type - defined - 2	5048400	181586	38
ΔpSymAB - rich - 1	16868958	196280	41
ΔpSymAB - rich - 2	14008712	188947	40
ΔpSymAB - defined - 1	11241306	199391	40
ΔpSymAB - defined - 2	10193372	200918	41

* The lower read count for the wild-type samples compared to the ΔpSymAB samples is predominately due to a significant portion of the reads mapping to the pSymA and pSymB replicons.

† The similarities in the number of unique insertion sites across replicates suggests that the libraries are of equal quality, and that differences in the level of saturation are not likely to explain differences between treatments.
