# Supplementary material for: Robustness encoded across essential and accessory replicons of the ecologically versatile bacterium Sinorhizobium meliloti
Source: PLoS Genet. 2018 Apr 19;14(4):e1007357. doi: 10.1371/journal.pgen.1007357 (PMC5929573; doi:10.1371/journal.pgen.1007357)
Supplement: S2 Table — (PDF) [file pgen.1007357.s005.pdf]

Table S2. Generation times* (hours) of *S. meliloti* strains.

	Rich medium		Defined medium	
Genotype †	RmP3499	RmP3496	RmP3499	RmP3496
No mutation	2.00	4.19	2.47	3.80
rhaK ¥	2.01	4.22	2.78	3.52
feuQ	2.06	2.95	2.77	3.42
coaA	1.95	4.21	3.00	3.93
tig	2.01	4.47	3.09	4.04
smc00074	1.97	4.16	3.01	3.62
ppk	1.94	4.57	2.92	5.10
smc00712	1.91	3.93	2.91	3.56
cbrA	2.11	8.75	3.52	6.23

* Generation times are calculated from the average of triplicate samples, and are shown in hours.

† The gene disrupted in each strain is indicated in the left column. The background strain that the mutation was constructed in is shown along the top.

¥ The *rhaK* mutant strains are meant as control strains as mutation of this gene is expected to have no phenotype under the tested conditions.
