# Supplementary material for: Robustness encoded across essential and accessory replicons of the ecologically versatile bacterium Sinorhizobium meliloti
Source: PLoS Genet. 2018 Apr 19;14(4):e1007357. doi: 10.1371/journal.pgen.1007357 (PMC5929573; doi:10.1371/journal.pgen.1007357)
Supplement: S3 Table — (PDF) [file pgen.1007357.s006.pdf]

**Table S3.** Core model (iGD726) biomass composition.

| Component | Percent dry mass | Composition - % |
| --- | --- | --- |
| DNA [a] | 2.8 | Guanine - 31.05<br>Cytosine - 31.05<br>Adenine - 18.95<br>Thymine - 18.95 |
| RNA [b] | 7.1 | Guanine - 28.09<br>Cytosine - 28.09<br>Adenine - 21.91<br>Uracil - 21.91 |
| Protein [c] | 49.3 | Lysine - 3.20<br>Alanine - 12.01<br>Leucine 10.19<br>Phenylalanine - 3.94<br>Arginine 7.33<br>Glutamine - 2.90<br>Glycine - 8.46<br>Methionine - 2.44<br>Valine - 7.58<br>Proline - 5.03<br>Tyrosine - 2.29<br>Aspartate - 5.31<br>Glutamate - 5.84<br>Histidine - 2.11<br>Threonine - 5.15<br>Cysteine - 0.93<br>Isoleucine - 5.48<br>Tryptophan - 1.38<br>Asparagine - 2.64<br>Serine - 5.80 |
| Phosphatidylglycerol [d] | 1.001 | Phosphatidylglycerol-1-palmitoleoyl-2-palmitic - 0.4<br>Phosphatidylglycerol-1,2-palmitic-2-palmitic - 1.4<br>Phosphatidylglycerol-1-cis-vaccenoyl-2-palmitoleoyl - 1.8<br>Phosphatidylglycerol-1-cis-vaccenoyl-2-palmitic - 24.9<br>Phosphatidylglycerol-1-palmitic-2-cis-vaccenoyl - 1.7<br>Phosphatidylglycerol-1-cis-vaccenoyl-2-palmitoleoyl(cyclopropanated) - 1.1<br>Phosphatidylglycerol-1-cis-vaccenoyl(cyclopropanated)-2-palmitoleoyl - 1.1<br>Phosphatidylglycerol-1-cis-vaccenoyl(cyclopropanated)-2-palmitic - 2.6<br>Phosphatidylglycerol-1-cis-vaccenoyl-2-cis-vaccenoyl - 57.3<br>Phosphatidylglycerol-1-cis-vaccenoyl(cyclopropanated)-2-cis-vaccenoyl - 7.3<br>Phosphatidylglycerol-1-cis-vaccenoyl(cyclopropanated)-2-cis-vaccenoyl(cyclopropanated) - 0.3 |
| Cardiolipin [d] | 0.398 | Cardiolipin-1,2-(1-palmitoleoyl-2-palmitic) - 0.4<br>Cardiolipin-1,2-(1,2-palmitic) - 1.4)<br>Cardiolipin-1,2-(1-cis-vaccenoyl-2-palmitoleoyl) - 1.8<br>Cardiolipin-1,2-(1-cis-vaccenoyl-2-palmitic) - 24.9<br>Cardiolipin-1,2-(1-palmitoleoyl-2-palmitic) - 1.7<br>Cardiolipin-1,2-(1-cis-vaccenoyl-2-palmitoleoyl(cyclopropanated)) - 1.1<br>Cardiolipin-1,2-(1-cis-vaccenoyl(cyclopropanated)-2-palmitoleoyl) - 1.1<br>Cardiolipin-1,2-(1-cis-vaccenoyl(cyclopropanated)-2-palmitic) - 2.6<br>Cardiolipin-1,2-(1-cis-vaccenoyl-2-cis-vaccenoyl) - 57.3<br>Cardiolipin-1,2-(1-cis-vaccenoyl(cyclopropanated)-2-cis-vaccenoyl) - 7.3<br>Cardiolipin-1,2-(1-cis-vaccenoyl(cyclopropanated)-2-cis-vaccenoyl(cyclopropanated)) - 0.3 |
| Phosphatidylethanolamine [d] | 1.754 | Phosphatidylethanolamine-1-cis-vaccenoyl-2-palmitoleoyl - 0.9<br>Phosphatidylethanolamine-1-cis-vaccenoyl-2-palmitic - 7.0<br>Phosphatidylethanolamine-1-cis-vaccenoyl-2-palmitoloeyl(cyclopropanated) - 1.05<br>Phosphatidylethanolamine-1-cis-vaccenoyl(cyclopropanated)-2-palmitoloeyl - 1.05<br>Phosphatidylethanolamine-1-cis-vaccenoyl(cyclopropanated)-2-palmitic - 2.9<br>Phosphatidylethanolamine-1,2-cis-vaccenoyl - 47.2<br>Phosphatidylethanolamine-1-cis-vaccenoyl(cyclopropanated)-2-cis-vaccenoyl - 35.5<br>Phosphatidylethanolamine-1-cis-vaccenoyl(cyclopropanated)-2-cis-vaccenoyl(cyclopropanated) - 4.4 |
| Monomethylethanolamine [d] | 1.491 | Monomethyl-phosphatidylethanolamine-1-cis-vaccenoyl-2-palmitoleoyl - 0.9<br>Monomethyl-phosphatidylethanolamine-1-cis-vaccenoyl-2-palmitic - 7.0<br>Monomethyl-phosphatidylethanolamine-1-cis-vaccenoyl-2-palmitoleoyl(cyclopropanated) - 1.05<br>Monomethyl-phosphatidylethanolamine-1-cis-vaccenoyl(cyclopropanated)-2-palmitoleoyl - 1.05<br>Monomethyl-phosphatidylethanolamine-1-cis-vaccenoyl(cyclopropanated)-2-palmitic - 2.9<br>Monomethyl-phosphatidylethanolamine-1,2-cis-vaccenoyl - 47.2<br>Monomethyl-phosphatidylethanolamine-1-cis-vaccenoyl(cyclopropanated)-2-cis-vaccenoyl - 35.5<br>Monomethyl-phosphatidylethanolamine-1-cis-vaccenoyl(cyclopropanated)-2-cis- |

| | | |
|---|---|---|
| | | vaccenoyl(cyclopropanated) - 4.4 |
| Phosphatidylcholine [d] | 7.670 | Phosphatidylcholine-1-cis-vaccenoyl-2-palmitoleoyl - 0.9<br>Phosphatidylcholine-1-cis-vaccenoyl-2-palmitic - 7.0<br>Phosphatidylcholine-1-cis-vaccenoyl-2-palmitoleoyl(cyclopropanated) - 1.05<br>Phosphatidylcholine-1-cis-vaccenoyl(cyclopropanated)-2-palmitoleoyl - 1.05<br>Phosphatidylcholine-1-cis-vaccenoyl(cyclopropanated)-2-palmitic - 2.9<br>Phosphatidylcholine-1,2-cis-vaccenoyl - 47.2<br>Phosphatidylcholine-1-cis-vaccenoyl(cyclopropanated)-2-cis-vaccenoyl - 35.5<br>Phosphatidylcholine-1-cis-vaccenoyl(cyclopropanated)-2-cis-vaccenoyl(cyclopropanated) - 4.4 |
| Sulfoquinovosyldiacylglycerol [d] | 0.256 | Sulfoquinovosyl-1-palmitoleoyl-2-palmitic-sn-glycerol - 3.0<br>Sulfoquinovosyl-1,2-palmitic-sn-glycerol - 17.2<br>Sulfoquinovosyl-1-cis-vaccenoyl-2-palmitoleoyl - 1.4<br>Sulfoquinovosyl-1-cis-vaccenoyl-2-palmitic - 21.8<br>Sulfoquinovosyl-1-palmitic-2-cis-vaccenoyl - 10.1<br>Sulfoquinovosyl-1-cis-vaccenoyl-2-palmitoleoyl(cyclopropanated) - 0.75<br>Sulfoquinovosyl-1-cis-vaccenoyl(cyclopropanated)-2-palmitoleoyl - 0.75<br>Sulfoquinovosyl-1-cis-vaccenoyl(cyclopropanated)-2-palmitic - 3.1<br>Sulfoquinovosyl-1-palmitic-2-cis-vaccenoyl(cyclopropanated)-sn-glycerol - 2.3<br>Sulfoquinovosyl-1-cis-vaccenoyl-2-cis-vaccenoyl-sn-glycerol - 17.8<br>Sulfoquinovosyl-1-cis-vaccenoyl(cyclopropanated)-2-cis-vaccenoyl-sn-glycerol - 10.5<br>Sulfoquinovosyl-1-cis-vaccenoyl(cyclopropanated)-2-cis-vaccenoyl(cyclopropanated)-sn-glycerol - 0.5<br>Sulfoquinovosyl-1-cis-vaccenoyl-steric-sn-glycerol - 10.8 |
| Ornithine lipids [d] | 0.230 | Ornithine-1-palmitic-2-cis-vaccenoyl - 0.5<br>Ornithine-1-steric-2-palmitoleoyl - 0.7<br>Ornithine-1-palmitic-2-cis-vaccenoyl(cyclopropanated) - 1.0<br>Ornithine-1-steric-2-palmitoleoyl(cyclopropanated) - 2.1<br>Ornithine-1-cis-vaccenoyl-2-cis-vaccenoyl - 3.0<br>Ornithine-1-steric-2-cis-vaccenoyl - 37.1<br>Ornithine-1-cis-vaccenoyl-2-cis-vaccenoyl(cyclopropanated) - 5.3<br>Ornithine-1-steric-2-cis-vaccenoyl(cyclopropanated) - 49.8<br>Ornithine-1-cis-vaccenoyl(cyclopropanated)-2-cis-vaccenoyl(cyclopropanated) - 0.6 |
| Poly-3-hydroxybutyrate | 17.6 | N/A |
| Glycogen | 0.4 | N/A |
| Lipopolysaccharide | 3 | N/A |
| Peptidoglycan | 2 | N/A |
| Low molecular weight succinoglycan [e] | 4 | N/A |
| High molecular weight succinoglycan[e] | 1 | N/A |
| Vitamins, cofactors, coenzymes, ions, and other [f] | Trace | Polyphosphate<br>Pantothenate<br>Coenzyme A<br>$NAD^+$; NADH<br>$NADP^+$; NADPH<br>$FAD^+$, FADH2<br>Folate; Tetrahydrofolate; 5,10-Methylenetetrahydrofolate<br>Thiamine diphosphate<br>Riboflavin<br>Biotin<br>Heme A<br>Vitamin B12 coenzyme<br>Undecaprenyl diphosphate<br>Ubiquinone-8<br>Pyridoxal phosphate<br>Glutathionine reduced<br>Glutathionine oxidized<br>All-trans-Phytoene<br>Holo-carboxylase<br>$Co^{2+}$ (Cobalt)<br>$Mg^+$ (Magnesium)<br>$Ca^{2+}$ (Calcium)<br>$Mn^{2+}$ (Manganese)<br>$Fe^{3+}$ (Iron)<br>$Fe^{2+}$ (Iron)<br>$Zn^{2+}$ (Zinc)<br>$K^+$ (Potassium)<br>$Na^+$ (Sodium) |

[a] Composition based on the overall GC content of *S. meliloti* [1].

[b] The GC content for mRNA was estimated from the overall GC content of *S. meliloti* [1]. The GC content of tRNA was estimated based on the GC content of the 10 most common codons in *S. meliloti* [1,2]. The GC content of rRNA was determined based on the *rrn* loci of *S. meliloti* [1]. The overall composition of cellular RNA was determined assuming 80% rRNA, 15% tRNA, and 5% mRNA.

[c] The amino acid composition was estimated based on the codon usage of *S. meliloti* [2].

[d] The membrane lipid composition used was as previously determined for *S. meliloti* [3].

[e] A 4:1 ratio of low molecular weight (LMW) to high molecular weight (HMW) succinoglycan was set as previously determined [4].

[f] Each of these compounds were included at an equal, trace concentration in the biomass.

## REFERENCES


1. Galibert F, Finan TM, Long SR, Pühler A, Abola AP, Ampe F, et al. The composite genome of the legume symbiont *Sinorhizobium meliloti*. Science. 2001;293: 668–672. doi:10.1126/science.1060966

2. Nakamura Y, Gojobori T, Ikemura T. Codon usage tabulated from international DNA sequence databases: status for the year 2000. Nucleic Acids Res. 1999;28: 292.

3. Basconcillo LS, Zaheer R, Finan TM, McCarry BE. A shotgun lipidomics study of a putative lysophosphatidic acid acyl transferase (PlsC) in *Sinorhizobium meliloti*. J Chromatogr B. 2009;877: 2873–2882. doi:10.1016/j.jchromb.2009.05.014

4. Glenn SA, Gurich N, Feeney MA, González JE. The ExpR/Sin quorum-sensing system controls succinoglycan production in *Sinorhizobium meliloti*. J Bacteriol. 2007;189: 7077–7088. doi:10.1128/JB.00906-07
