# Supplementary material for: Robustness encoded across essential and accessory replicons of the ecologically versatile bacterium Sinorhizobium meliloti
Source: PLoS Genet. 2018 Apr 19;14(4):e1007357. doi: 10.1371/journal.pgen.1007357 (PMC5929573; doi:10.1371/journal.pgen.1007357)
Supplement: S4 Table — (PDF) [file pgen.1007357.s007.pdf]

Table S4. Newly predicted functions for eight genes previously hypothetical or broadly annotated genes.

Locus Tag	New Name	Original annotation	New Annotation	Essential in Tn-seq?	Notes
High confidence in predictions					
smc00617	*hda*	Conserved hypothetical protein	DnaA regulatory inactivator	Yes	The Smc00617 protein was essential in the Tn-seq data. A Blastp search on the NCBI webserver reveals similarity to DnaA domain (evalue < 1e-50). As *S. meliloti* contains an essential, annotated DnaA protein, we suggest that Smc00617 is not DnaA, but an ortholog of the *E. coli* Hda protein (24% identity between Had and Smc00617 of 51% of the length of both proteins), which is a DnaA-related protein involved in DNA replication control [1].
smc01361	*pyrX*	Dihydroorotase	Inactive dihydroorotase	Yes	In some organisms like *Pseudomonas putida*, the PyrB protein requires an inactive dihydroorotase protein to function [2,3]. The Smc01361 protein is encoded downstream of the PyrB protein, is annotated as a dihydroorotase, and appeared essential in this Tn-seq study. As an essential PyrC dihydroorotoase is encoded elsewhere in the genome, we postulate that Smc01361 is an inactive dihydroorotase required for PyrB function.
smc01362	*plsY*	Hypothetical transmembrane protein	SN-glycerol-3-phosphate acetyltransferase	Yes	The *smc01362* gene product shows similarity to PlsY proteins and it appeared essential in this Tn-seq data. Therefore, we have annotated this gene as *plsY*.
smc02090	*lpxI*	Conserved hypothetical protein	UDP-2,3-diacylglucosamine pyrophosphatase	Yes	The gene *smc02090* is located in a locus containing lipopolysaccharide biosynthetic genes. It also appeared essential in this Tn-seq data. Therefore we have reannotated this gene as *lpxI*, whose product replaces the function of the LpxH protein of *E. coli* based on previous work with *C. crescentus* [4].
smc04042	-	Inositol-1-monophosphatase family protein	L-Histidinol-phosphate phosphohydrolase	Yes	It was previously noted [5] that rhizobia lack the classical enzyme catalyzing this reaction and that the HisB enzymes of rhizobia are unlikely to be bifunctional and also perform this role. Instead, it was hypothesized that an inositol monophosphatase family protein could fulfill this function [5]. As Smc04042 is an inositol monophosphatase family protein that appears essential in Tn-seq work (Tn-seq), we postulate that Smc04042 might fulfill this role.
Weak confidence in predictions					
smc04014	*pabC*	Hypothetical protein	4-amino-4-deoxychorismate lyase	No	Given that *smc04014* is adjacent to *pabB* and its product has (weak) similarity to PabC enzymes, we predict this gene encodes a PabC enzyme and have annotated it as such. However, *smc04014* did not appear essential in this Tn-seq data, suggesting that if PabC catalyzes this reaction, there must be a bypass or another enzyme capable of complementing for its loss. One possibility would be TrpG.
smc03995	-	Conserved hypothetical protein, signal peptide	Thiamine-phosphate kinase	Yes	The *smc03995* gene appeared essential in this Tn-seq data. As this gene appears to be part of an operon with *thiE2* (involved in the previous reaction in thiamine biosynthesis), and as the protein functioning as the thiamine kinase in *S. meliloti* has not been identified, we predict that Smc03995 may fulfill this function. However, this may be incorrect, particularly as thiamine was included in the medium, which we expect should have rendered this reaction non-essential (and thus *smc03995* still being essential may suggest it plays a different function).
smc01026	-	Hypothetical/unknown protein	3-deoxy-manno-octulosonate-8-phosphatase	No	An enzyme performing this activity has not been identified in *S. meliloti*. We have annotated Smc01026 as this enzyme as a prediction based on co-occurrence with the *kdsA* gene in *S. meliloti* (likely an operon) and in other organisms (see the STRING database). However, the gene did not appear to be essential in this Tn-seq data, and thus if this protein does have this function, there must be another protein able to complement its loss.

REFERENCES

1. Kato J, Katayama T. Hda, a novel DnaA-related protein, regulates the replication cycle in *Escherichia coli*. EMBO J. 2001;20: 4253–4262. doi:10.1093/emboj/20.15.4253
2. Schurr MJ, Vickrey JF, Kumar AP, Campbell AL, Cunin R, Benjamin RC, et al. Aspartate transcarbamoylase genes of *Pseudomonas putida*: requirement for an inactive dihydroorotase for assembly into the dodecameric holoenzyme. J Bacteriol. 1995;177: 1751–1759.
3. Labedan B, Xu Y, Naumoff DG, Glansdorff N. Using quaternary structures to assess the evolutionary history of proteins: the case of the aspartate carbamoyltransferase. Mol Biol Evol. 2004;21: 364–373. doi:10.1093/molbev/msh024
4. Metzger LE, Raetz CRH. An alternative route for UDP-diacylglucosamine hydrolysis in bacterial lipid A biosynthesis. Biochemistry. 2010;49: 6715–6726. doi:10.1021/bi1008744
5. Dunn MF. Key roles of microsymbiont amino acid metabolism in rhizobia-legume interactions. Crit Rev in Microbiol. 2015;41: 411–451. doi:10.3109/1040841X.2013.856854
