# Supplementary material for: Robustness encoded across essential and accessory replicons of the ecologically versatile bacterium Sinorhizobium meliloti
Source: PLoS Genet. 2018 Apr 19;14(4):e1007357. doi: 10.1371/journal.pgen.1007357 (PMC5929573; doi:10.1371/journal.pgen.1007357)
Supplement: S5 Table — (PDF) [file pgen.1007357.s008.pdf]

Table S5. Bacterial strains and plasmids.

Strains or plasmids	Relevant characteristics/description	Source or reference
Sinorhizobium meliloti		
RmP3496	*S. meliloti* ΔpSymAB	[1]
RmP3499	RmP3496 with pSymA and pSymB re-introduced	[1]
AB001	Δ*proC in* RmP3496	This study
AB002	Δ*proC* in RmP3499	This study
AB003	Δ*cbrA in* RmP3496	This study
AB004	Δ*cbrA* in RmP3499	This study
AB005	Δ*tig* in RmP3496	This study
AB006	Δ*tig* in RmP3499	This study
AB007	Δ*feuQ* in RmP3496	This study
AB008	Δ*feuQ* in RmP3499	This study
AB009	Δ*ppk* in RmP3496	This study
AB010	Δ*ppk* in RmP3499	This study
AB011	Δ*smc00712* in RmP3496	This study
AB012	Δ*smc00712* in RmP3499	This study
AB013	Δ*rhaK* in RmP3496	This study
AB014	Δ*rhaK* in RmP3499	This study
AB015	Δ*amiC* in RmP3499	This study
AB016	Δ*fbpB* in RmP3499	This study
AB017	Δ*aidB* in RmP3499	This study
AB018	Δ*smc00575* in RmP3499	This study
AB019	Δ*tal* in RmP3499	This study
AB020	Δ*kpsF3* in RmP3499	This study
AB026	Δ*carA* in RmP3499	This study
AB021	Δ*carA* in RmP3496	This study
AB022	Δ*exoN2* in RmP3499	This study
AB024	Δ*coaA* in RmP3499	This study
AB033	Δ*coaA* in RmP3496	This study
AB025	Δ*smc00074* in RmP3499	This study
AB034	Δ*smc00074* in RmP3496	This study
AB028	Δ*smc01361* in RmP3499	This study
AB029	Δ*argD* in RmP3499	This study
AB037	Δ*argD* in RmP3496	This study
AB030	Δ*ilvC* in RmP3499	This study
AB031	Δ*serC* in RmP3499	This study
AB032	Δ*smc04042* in RmP3499	This study
Escherichia coli		
DH5α	*E. coli* cloning and expression strain	[2]
B001	*DH5α* harboring helper plasmid pRK600 (Cm^R)	[3]
Plasmids		
pJG194	2.2 kb mobilizable suicide plasmid (Km^R/Nm^R)	[4]
pRK600	Self-transmissible helper plasmid (Cm^R)	[3]
pJG714	Transposon delivery vector for Tn-seq (Km^R/Nm^R)	This study
pAB001	*ftsK* fragment cloned into pJG194	This study
pAB004	*proC* fragment cloned into pJG194	This study
pAB006	*smc00074* fragment cloned into pJG194	This study
pAB008	*amiC* fragment cloned into pJG194	This study
pAB010	*fbpB* fragment cloned into pJG194	This study
pAB012	*cbrA* fragment cloned into pJG194	This study
pAB014	*tig* fragment cloned into pJG194	This study
pAB016	*feuQ* fragment cloned into pJG194	This study
pAB018	*aidB* fragment cloned into pJG194	This study
pAB019	*ppk* fragment cloned into pJG194	This study

pAB021	*smc00575* fragment cloned into pJG194	This study
pAB023	*tal* fragment cloned into pJG194	This study
pAB024	*kspF3* fragment cloned into pJG194	This study
pAB025	*exoN2* fragment cloned into pJG194	This study
pAB026	*smc00712* fragment cloned into pJG194	This study
pAB027	*carA* fragment cloned into pJG194	This study
pAB028	*coaA* fragment cloned into pJG194	This study
pAB031	*smc02836* fragment cloned into pJG194	This study
pAB032	*rhaK* fragment cloned into pJG194	This study
pAB033	*argD* fragment cloned into pJG194	This study
pAB034	*smc04042* fragment cloned into pJG194	This study
pAB035	*smc01361* fragment cloned into pJG194	This study
pAB036	*ilvC* fragment cloned into pJG194	This study
pAB037	*serC* fragment cloned into pJG194	This study
pAB038	*smc01011* knock out 1 fragment cloned into pJG194	This study
pAB039	*smc01011* knock out 2 fragment cloned into pJG194	This study
pAB040	*smc01011* Non-disruption fragment cloned into pJG194	This study
pAB041	*smc03995* knock out 1 fragment cloned into pJG194	This study
pAB042	*smc03995* knock out 2 fragment cloned into pJG194	This study
pAB043	*smc03995* Non-disruption fragment cloned into pJG194	This study
pAB047	*fumC* Non-disruption fragment cloned into pJG194	This study
pAB048	*fumC* knock out 1 fragment cloned into pJG194	This study
pAB049	*fumC* knock out 2 fragment cloned into pJG194	This study
pAB050	*pdxJ* Non-disruption fragment cloned into pJG194	This study
pAB051	*pdxJ* knock out 1 fragment cloned into pJG194	This study
pAB052	*pdxJ* knock out 2 fragment cloned into pJG194	This study

REFERENCES

1. diCenzo GC, Zamani M, Milunovic B, Finan TM. Genomic resources for identification of the minimal N_2-fixing symbiotic genome. Environ Microbiol. 2016;18: 2534–2547. doi:10.1111/1462-2920.13221

2. Grant SG, Jessee J, Bloom FR, Hanahan D. Differential plasmid rescue from transgenic mouse DNAs into *Escherichia coli* methylation-restriction mutants. Proc Natl Acad Sci USA. 1990;87: 4645–4649.

3. Finan TM, Kunkel B, De Vos GF, Signer ER. Second symbiotic megaplasmid in *Rhizobium meliloti* carrying exopolysaccharide and thiamine synthesis genes. J Bacteriol. 1986;167: 66–72.

4. Griffitts JS, Long SR. A symbiotic mutant of *Sinorhizobium meliloti* reveals a novel genetic pathway involving succinoglycan biosynthetic functions. Mol Microbiol. 2008;67: 1292–1306. doi:10.1111/j.1365-2958.2008.06123.x
