# Supplementary material for: Robustness encoded across essential and accessory replicons of the ecologically versatile bacterium Sinorhizobium meliloti
Source: PLoS Genet. 2018 Apr 19;14(4):e1007357. doi: 10.1371/journal.pgen.1007357 (PMC5929573; doi:10.1371/journal.pgen.1007357)
Supplement: S6 Table — (PDF) [file pgen.1007357.s009.pdf]

**Table S6.** Oligonucleotides used in this study.

| Name[a] | Oligonucleotide sequence[b] |
|---|---|
| **Tn-seq primers** | |
| 1TN | CTGACCCGGTCGAC |
| 1GG | CAGACGTGTGCTCTTCCGATCgggggggggg |
| 2TNA | AATGATACGGCGACCACCGAGATCTACACTCTTTCCCTACACGACGCTCTTCCGATCTTCGAGATGTGTATAAGAGACAG |
| 2TNB | AATGATACGGCGACCACCGAGATCTACACTCTTTCCCTACACGACGCTCTTCCGATCTATCGAGATGTGTATAAGAGACAG |
| 2TNC | AATGATACGGCGACCACCGAGATCTACACTCTTTCCCTACACGACGCTCTTCCGATCTGATCGAGATGTGTATAAGAGACAG |
| 2BAR1 | CAAGCAGAAGACGGCATACGAGATCGTGATGTGACTGGAGTTCAGACGTGTGCTCTTCCGATC |
| 2BAR2 | CAAGCAGAAGACGGCATACGAGATACATCGGTGACTGGAGTTCAGACGTGTGCTCTTCCGATC |
| 2BAR3 | CAAGCAGAAGACGGCATACGAGATGCCTAAGTGACTGGAGTTCAGACGTGTGCTCTTCCGATC |
| 2BAR4 | CAAGCAGAAGACGGCATACGAGATATTGGCGTGACTGGAGTTCAGACGTGTGCTCTTCCGATC |
| 2BAR5 | CAAGCAGAAGACGGCATACGAGATCACTGTGTGACTGGAGTTCAGACGTGTGCTCTTCCGATC |
| 2BAR6 | CAAGCAGAAGACGGCATACGAGATGATCTGGTGACTGGAGTTCAGACGTGTGCTCTTCCGATC |
| 2BAR7 | CAAGCAGAAGACGGCATACGAGATTGGTCAGTGACTGGAGTTCAGACGTGTGCTCTTCCGATC |
| 2BAR8 | CAAGCAGAAGACGGCATACGAGATCTGATCGTGACTGGAGTTCAGACGTGTGCTCTTCCGATC |
| ***smc01335 -amiC* Disruption** | |
| 01335F | 5'- cccgaattcAAGTGAAGCCGGTGGAGG |
| 01335R | 5'- gggaagcttAACGTGTCGGCGTGAACCG |
| 01335C | 5'- ATCGTGTAGACCGTGGCGCC |
| ***smc00074* Disruption** | |
| 00074F | 5'- cccgaattcTCGCAGCCGGTCTCGTGAT |
| 00074R | 5'- gggaagcttGGTATCGCCTGATCCCGT |
| 00074C | 5'- CGTTCATCGTTCCGAGCG |
| ***smc02050 -tig* Disruption** | |
| 02050F | 5'- cccgaattcTCGACGGCGAAGCCTTCG |
| 02050R | 5'- gggaagcttAGGCGCTCGGGTGTATCG |
| 02050C | 5'- CGGTGTTGATCTGGCGCC |
| ***smc00129 -feuQ* Disruption** | |
| 00129F | 5'- cccgaattcTATCGACCGCTTCACGCG |
| 00129R | 5'- gggaagcttTGGCGCGGCTGAGATAGG |
| 00129C | 5'- GAACCGACCCGCGCTGGG |
| ***smc00776 -cbrA* Disruption** | |
| 00776F | 5'- cccgaattcGGCAGTGGGAAAGATGCG |
| 00776R | 5'- gggaagcttTCCATTGCCGCAGCAGGG |
| 00776C | 5'- GCTGCTCATGGCTGCACC |
| ***smc03808 -ftsK* Disruption:** | |
| 03808F | 5'- gacgaattcGGCACATTGAACGAGCCGG |
| 03808R | 5'- gggaagcttACATGGATGATCTCGCCC |
| 03808C | 5'- GCCGGTTCCAGCTCATAG |
| ***smc01359 -aidB* Disruption** | |
| 01359F | 5'- cccgaattcTACCTCCACGGCCGAACG |
| 01359R | 5'- gggaagcttTATCGAAGGCTTCGGCCAGG |
| 01359C | 5'- TCGCGACCGGCGTCATGAT |
| ***smc00575* Disruption** | |
| 00575F | 5'- cccgaattcGCCGCTGGAGGCGAACAAGAA |
| 00575R | 5'- gggaagcttGCAGCGGTCATCTGCTTG |
| 00575C | 5'- CTCCCAGATCTGCTGCAC |
| ***smc00618* ppk Disruption** | |
| 00618F | 5'- cccgaattcAGGAACTCGGCGTTCCCG |
| 00618R | 5'- gggaagcttCGGATATTTGCCTCCTCG |
| 00618C | 5'- CCGAAAACCACCTGCACG |
| ***smc00775 -fbpB* Disruption** | |
| 00775F | 5'- cccgaattcTCACGCTCGTTGCCATGG |
| 00775R | 5'- gggaagcttCAGGAACTGGTCGAGGCG |
| 00775C | 5'- GACACCAGGATGCTGTGC |
| ***smc02677 -proC* Disruption** | |
| 02677F | 5'- cccgaattcTCTTCCTCGCGGTCAAGC |
| 02677R | 5'- gggaagcttTAGGTCCGCCTCGAGTCC |
| 02677C | 5'- GGTTTCCCGAGCAAGACG |
| ***smc02495 -tal* Disruption** | |
| 02495F | 5'- cccgaattcCGACATCGTCGAGGTTACCA |
| 02495R | 5'- gggaagcttGGCTTCCTTCACGTGGTTGA |
| 02495C | 5'- CCTTCCACTCCATGCCATT |
| ***smc02268 -kpsF3* Disruption** | |
| 02268F | 5'- cccgaattcATGATCACCGCTCAGGATGC |
| 02268R | 5'- gggaagcttGTGATGACGCCGATCAGCTT |
| 02268C | 5'- CGGTGAGACTCAGCGAAGA |
| ***smc04023 -exoN2*** | |

04023F 5'- cccgaattcGGACAAGCCGGTCATCCAAT
04023R 5'- gggaagcttTCGACCATCCTGGTGATCCT
04023C 5'- GCAGGATGTAGCGGCCATT
***smc00712* -hypothetical**
00712F 5'- cccgaattcAGATCCAGAAGCGGCTGACT
00712R 5'- gggaagcttATGCTGAGTGCCGCGTCTA
00712C 5'- ACTCTGCCTTGTCCGCAA
***smc03826 -argG***
03826F 5'- cccgaattcGCTCGGCATCAAGGAGATCT
03826R 5'- gggaagcttTGCGCATGTGCACGTATTC
03826C 5'- CTTGTCAGGTGCCGCTTC
***smc01569 -carA***
01569F 5'- cccgaattcAGGACATCGAGGATCTGACG
01569R 5'- gggaagcttTCGAGAGCCACGACGTGATA
01569C 5'- ATGACCGGCACGGCATATT
***smc02567 -coaA***
02567F 5'- cccgaattcAACGATCCGGTCGATCTCGA
02567R 5'- gggaagcttATAGGTCGGAGCCTTGACGT
02567C 5'- AGACCATCGGCACGATCTT
***smc01011* -KO1**
01011KO1F 5'- cccgaattcTCAGCCCGCTCATCACCGAT
01011KO1R 5'- gggaagcttCCATCCTCCTTCACGTCGGA
***smc01011* - KO2**
01011KO2F 5'- cccgaattcAAGATTCGCGTGCCCGAAGA
01011KO2R 5'- gggaagcttGAGGATCGACCAGGCGGTAT
***smc01011* - ND**
01011NDF 5'- cccgaattcCCGCAACAAGGACAAGATCT
01011NDR 5'- gggaagcttACGGCAATGTCGCTGGACGA
01011NDC 5'- GAGGAGCGCTCGGTCAACAT
***smc03995* - KO1**
03995KO1F 5'- cccgaattcATCACGCCTTTCAACGGCGC
03995KO1R 5'- gggaagcttTTCATCAGTTCGTCGGCCCT
***smc03995* - KO2**
03995KO2F 5'- cccgaattcATGCGCTGATCCTGATGGAG
03995KO2R 5'- gggaagcttGAGATGGGAGAGCCGGTTCT
***smc03995* - ND**
03995NDF 5'- cccgaattcAAGTGGTACGTGCTCTCGCG
03995NDR 5'- gggaagcttTTGAAGACGGCCTGGACCAT
03995NDC 5'- GTACTTCGCCGAAATCGCGT
***smc00149 -fumC* KO1**
00149KO1F 5'- cccgaattcCGGAACCCAGTCGAACATGA
00149KO1R 5'- gggaagcttTTGATCGAGGAGGCGACCTG
***smc00149 -fumC* KO2**
00149KO2F 5'- cccgaattcACATCATCAAGATCGGCCGC
00149KO2R 5'- gggaagcttTTGACCTTGCCCGGCATGAT
***smc00149 -fumC* ND**
00149NDF 5'- cccgaattcATGCTGGTGACTGCGCTTGC
00149NDR 5'- gggaagcttTCATCGTGGTCACGATCGGC
00149NDC 5'- CTGCCTTGACCGGTTCCTTC
***smc01407 -pdxJ* KO1**
01407KO1F 5'- cccGTCGACTTCCGATCTCCAGCCGATCC
01407KO1R 5'- gggCTCGAGTAGAGTTCTATGCGGTCGGC
***smc01407 -pdxJ* KO2**
01407KO2F 5'- cccGTCGACTCGCAAGAGCCATAACCTGC
01407KO2R 5'- gggCTCGAGGGTCTCGGCCATTCCGTATT
***smc01407 -pdxJ* ND**
01407NDF 5'- cccgaattcGAAGTCTCGATCGGACACGG
01407NDR 5'- gggaagcttGCAATTGCGAGATACAGCGG
01407NDC 5'- TAAAGTCGATGCCGAGCGCG
***smc02138 -argD***
02138F 5'- cccgaattcTCTTCACCAATTCGGGTGCG
02138R 5'- gggaagcttTTCATGGGCGAAGAGCTTGC
02138C 5'- TTCGGCGAGAACGACGTCGA
***smc04042***
04042F 5'- cccgaattcTGCATTCATCTCCGGCCTGC
04042R 5'- gggaagcttTGCTCGATCAGCGGAATGAG
04042C 5'- CCTGCCAGTCGGTGATGATT
***smc01361***
01361F 5'- cccgaattcTGCACGATACGCTCGTGCTT
01361R 5'- gggaagcttTCGACGTCCTGCGGATCATG

| | |
|---|---|
| 01361C | 5'- TCAAGGTCGATGACGGCGAT |
| ***smc04346 -ilvC*** | |
| 04346F | 5'- cccgaattcACGGCCTCAACGTCCACTTC |
| 04346R | 5'- gggaagcttCGACGATCAGCTTCACTTCG |
| 04346C | 5'- TGTCTTCGGTGATGATGCGC |
| ***smc00640 -serC*** | |
| 00640F | 5'- cccgaattcGACGTGGTCTTCACCTGGAA |
| 00640R | 5'- gggaagcttCCAGAGAAGCGCGTCGATAT |
| 00640C | 5'- AGGTTGGCGATCCAGTCGTT |
| ***smb20960 -exoN*** | |
| 20960F | 5'- cgcggatccTTCCGAACTCGAGGCCATGT |
| 20960R | 5'- gcgtctagaGCCATCGGTCAACTGGATCT |
| 20960C | 5'- GAAGGCGACATTGGCTTCGA |
| 20960C | 5'- ACGACGAATGCGGCCACAAG |
| **pJG520 vector check primer** | |
| VCpJG520 | 5'- CCGCAGTGGCTCTCTATACA |
| ***smc03003 -rhaK* non-disruption control** | |
| 03003F | 5'- cccgaattcCTGAACAGCACGGGAACGAG |
| 03003R | 5'- gggaagcttCATACAGCGCCATGCGTCTC |
| 03003C | 5'- AACGCAGCCACAGGTTTTC |

[a]Primer names indicate whether the primer was used for insert amplification (F, R) or for confirming plasmid integration in *S. meliloti* (C).

[b]Nucleotides annealing to the template are in uppercase letters. Five prime extensions to facilitate cloning are in lowercase letters. Underlined nucleotides in the Tn-seq primers indicate locations of sequence variability in each series of primers.
