# Supplementary material for: Robustness encoded across essential and accessory replicons of the ecologically versatile bacterium Sinorhizobium meliloti
Source: PLoS Genet. 2018 Apr 19;14(4):e1007357. doi: 10.1371/journal.pgen.1007357 (PMC5929573; doi:10.1371/journal.pgen.1007357)
Supplement: S7 Table — (PDF) [file pgen.1007357.s010.pdf]

Table S7. Boundary conditions for *in silico* metabolic modeling analyses.

Exchange reaction	Compound	Lower boundary	Upper Boundary
EX_cpd00013_e0	Ammonia (NH_3)	-10	1000
EX_cpd00001_e0	Water (H_2O)	-10	1000
EX_cpd00007_e0	Oxygen (O_2)	-10	1000
EX_cpd00009_e0	Phosphate (PO_4^{3-})	-10	1000
EX_cpd00011_e0	Carbon dioxide (CO_2)	-10	1000
EX_cpd00030_e0	Manganese (Mn^{2+})	-10	1000
EX_cpd00034_e0	Zinc (Zn^{2+})	-10	1000
EX_cpd00048_e0	Sulfate (SO_4^{2-})	-10	1000
EX_cpd00058_e0	Copper (Cu^{2+})	-10	1000
EX_cpd00063_e0	Calcium (Ca^{2+})	-10	1000
EX_cpd00067_e0	Proton (H^+)	-10	1000
EX_cpd00076_e0	Sucrose	-1	1000
EX_cpd00099_e0	Chloride (Cl^-)	-10	1000
EX_cpd00104_e0	Biotin	-10	1000
EX_cpd00149_e0	Cobalt (Co^{2+})	-10	1000
EX_cpd00205_e0	Potassium (K^+)	-10	1000
EX_cpd00254_e0	Magnesium (Mg^+)	-10	1000
EX_cpd00971_e0	Sodium (Na^+)	-10	1000
EX_cpd10515_e0	Iron (Fe^{2+})	-10	1000
EX_cpd10516_e0	Iron (Fe^{3+})	-10	1000
EX_cpd00305_e0	Thiamine	-10	1000

All values are given in units of: mmol (g cell dry weight)$^{-1}$ hr^{-1}. All exchange reactions not listed has a lower bound of ‘0’ and an upper bound of ‘1000’.
