# Supplementary material for: Robustness encoded across essential and accessory replicons of the ecologically versatile bacterium Sinorhizobium meliloti
Source: PLoS Genet. 2018 Apr 19;14(4):e1007357. doi: 10.1371/journal.pgen.1007357 (PMC5929573; doi:10.1371/journal.pgen.1007357)
Supplement: S8 Table — (PDF) [file pgen.1007357.s011.pdf]

**Table S8.** Biomass composition of iGD1575b and iGD1575c.[a]

| Component | Percent dry mass | Composition - % |
|---|---|---|
| DNA | 2.8 | Guanine - 31.05<br>Cytosine - 31.05<br>Adenine - 18.95<br>Thymine - 18.95 |
| RNA | 7.1 | Guanine - 28.09<br>Cytosine - 28.09<br>Adenine - 21.91<br>Uracil - 21.91 |
| Protein | 49.3 | Lysine - 3.20<br>Alanine - 12.01<br>Leucine 10.19<br>Phenylalanine - 3.94<br>Arginine 7.33<br>Glutamine - 2.90<br>Glycine - 8.46<br>Methionine - 2.44<br>Valine - 7.58<br>Proline - 5.03<br>Tyrosine - 2.29<br>Aspartate - 5.31<br>Glutamate - 5.84<br>Histidine - 2.11<br>Threonine - 5.15<br>Cysteine - 0.93<br>Isoleucine - 5.48<br>Tryptophan - 1.38<br>Asparagine - 2.64<br>Serine - 5.80 |
| Lipid | 12.8 | PG(36:2) - 7.82<br>CL(36:2) - 3.11<br>PE(36:2) - 25.35<br>PC(36:2) - 59.92<br>SL(36:2) - 2.00<br>OL(36:1) - 1.80 |
| PHB | 17.6 | N/A |
| Glycogen | 0.4 | N/A |
| LPS | 3 | N/A |
| Cell wall | 2 | N/A |
| LMW Succinoglycan [b] | 4 | N/A |
| HMW Succinoglycan [b] | 1 | N/A |
| Putrescine | Trace | N/A |

| | | |
|---|---|---|
| Spermidine | Trace | N/A |
| Vitamins, cofactors, coenzymes, ions, and other | Trace | Polyphosphate<br>Pantothenate<br>Coenzyme A<br>$NAD^+$; NADH<br>$NADP^+$; NADPH<br>$FAD^+$, FADH2<br>Folate; Tetrahydrofolate; 5,10-Methylenetetrahydrofolate<br>Thiamine diphosphate<br>Riboflavin<br>Biotin<br>Heme A<br>Vitamin B12 coenzyme<br>Undecaprenyl diphosphate<br>Ubiquinone-8<br>Pyridoxal phosphate<br>Glutathionine reduced<br>Glutathionine oxidized<br>Holo-carboxylase<br>$Co^{2+}$ (Cobalt)<br>$Mg^+$ (Magnesium)<br>$Ca^{2+}$ (Calcium)<br>$Mn^{2+}$ (Manganese)<br>$Fe^{3+}$ (Iron)<br>$Fe^{2+}$ (Iron)<br>$Zn^{2+}$ (Zinc)<br>$K^+$ (Potassium)<br>$Na^+$ (Sodium) |

[a] The composition of all components was set as described previously for iGD1575 [1].The exception are the vitamins, cofactors, coenzymes, and ions that are newly added, and were included at an equal, trace concentration in the biomass.

[b] Succinoglycan was removed from the biomass of iGD1575c.

## REFERENCES

1. diCenzo GC, Checcucci A, Bazzicalupo M, Mengoni A, Viti C, Dziewit L, et al. Metabolic modelling reveals the specialization of secondary replicons for niche adaptation in *Sinorhizobium meliloti*. Nat Commun. 2016;7: 12219. doi:10.1038/ncomms12219
