# Supplementary material for: Robustness encoded across essential and accessory replicons of the ecologically versatile bacterium Sinorhizobium meliloti
Source: PLoS Genet. 2018 Apr 19;14(4):e1007357. doi: 10.1371/journal.pgen.1007357 (PMC5929573; doi:10.1371/journal.pgen.1007357)
Supplement: S9 Table — (PDF) [file pgen.1007357.s012.pdf]

Table S9. Gene essentiality data represented in Figure 6.

Gene	Wild-type GEI	ΔpSymAB GEI	log10(GEI ratio [ΔpSymAB / wild-type])
Cytochrome C oxidase related proteins, defined medium			
ctaC	0.812	0.040	-1.313
ctaD	0.107	0.011	-0.979
ctaB	0.081	0.061	-0.123
ctaG	1.454	0.146	-0.999
ctaE	0.168	0.014	-1.091
ccsA	0.401	0.007	-1.775
cycH	2.037	1.559	-0.116
cycJ	1.344	0.658	-0.310
cycK	1.454	0.140	-1.016
cycL	1.077	0.252	-0.630
ccmA	0.990	0.131	-0.877
ccmB	1.664	0.130	-1.106
ccmC	1.265	0.272	-0.667
ccmD	1.367	0.011	-2.083
ccmG	2.368	0.264	-0.952
Proline biosynthesis, rich medium			
proB1	7.335	0.267	-1.438
proA	4.505	0.002	-3.285
proC	2.250	0.002	-2.964
Histidine biosynthesis, rich medium *			
hisB	2.530	0.010	-2.410
smc04042	0.600	0.062	-0.989
hisD	0.103	0.008	-1.089
Glycolysis and related proteins, rich medium			
glk	2.613	0.342	-0.883
frk	0.673	0.008	-1.950
pgi	0.001	0.001	0.000
zwf	0.235	0.001	-2.239
pgl	0.658	0.003	-2.362
edd	3.284	0.063	-1.720
eda2	1.903	0.698	-0.436
gap	1.300	0.002	-2.818
pgk	1.928	0.024	-1.902
gpmA	2.637	1.538	-0.234
eno	1.110	1.129	0.007
pykA	2.017	0.424	-0.678
pyc	4.409	0.223	-1.296
Periplasmic cyclic β-glucans biosynthesis, defined medium			
feuN	1.095	0.282	-0.590
feuP	0.938	0.003	-2.498
feuQ	10.855	0.012	-2.947
ndvA	1.434	0.001	-3.123
ndvB	3.218	0.129	-1.396

Arginine biosynthesis, rich medium			
argB	0.802	2.125	0.423
argC	0.636	3.618	0.755
argD	1.499	1.467	-0.010
argJ	3.118	4.134	0.122
argF1	0.012	1.776	2.168
argG	0.002	1.281	2.892
argH1	0.198	1.011	0.708
AICAR biosynthesis, rich medium			
purF	0.506	2.273	0.652
purD	0.716	2.770	0.588
purN	1.962	3.974	0.307
smc00494	0.469	2.774	0.772
purL	0.996	5.689	0.757
purQ	0.908	2.351	0.413
purM	1.060	3.070	0.462
purE	1.387	2.399	0.238
purK	2.029	3.562	0.244
purC	0.302	0.175	-0.237
purB	0.002	0.799	2.542
purH	0.624	0.705	0.053
UMP biosynthesis, rich medium			
carA	0.007	3.928	2.721
carB	0.001	1.930	3.130
pyrB	0.062	2.364	1.584
smc01361	0.417	2.793	0.826
pyrC	0.134	0.689	0.711
pyrD	0.193	2.700	1.146
pyrE	0.300	2.266	0.878
pyrF	0.323	4.956	1.185
LPS core oligosaccharide, defined medium			
lpsC	0.101	1.555	1.188
lpsD	1.154	2.969	0.410
lpsE	0.832	2.609	0.496

* Represents just the final steps of this process, from the conversion of imidazole-acetol-phosphate to L-histidine.
