# Supplementary figures and images for: Robustness encoded across essential and accessory replicons of the ecologically versatile bacterium Sinorhizobium meliloti
Source: PLoS Genet. 2018 Apr 19;14(4):e1007357. doi: 10.1371/journal.pgen.1007357 (PMC5929573; doi:10.1371/journal.pgen.1007357)

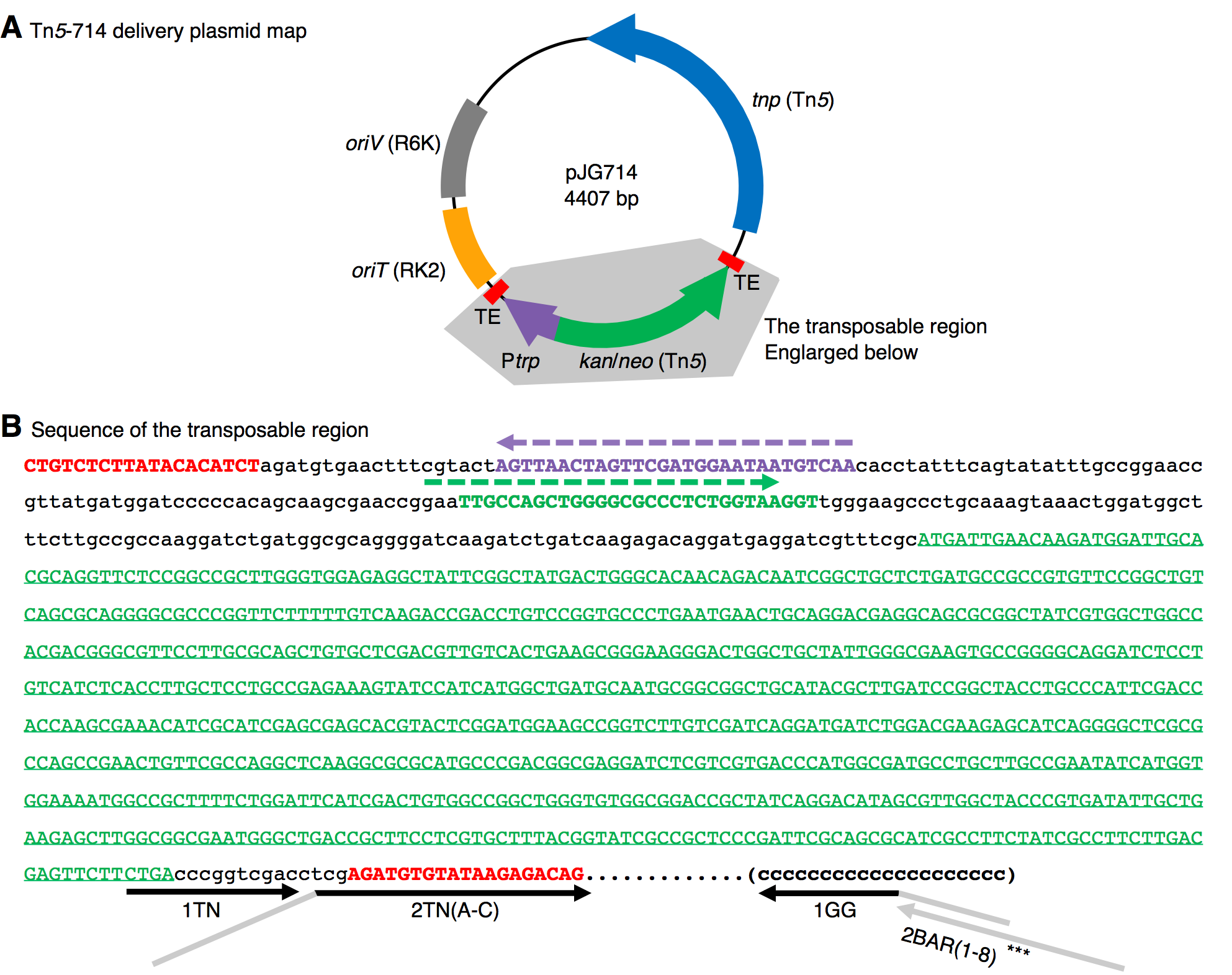

Supplement: S1 Fig — (A) Genetic map of the delivery vector pJG714, indicating the locations of the transposase-encoding gene (tnp), transposon ends (TE), the kanamycin/neomycin resistance determinant (kan/neo), the Salmonella trp promoter (Ptrp), the cis-acting mobilization determinant from plasmid RK2 (oriT), and the pir-dependent replication origin from plasmid R6K (oriV). The transposable region is highlighted in grey. No transcriptional stop sequences are present between the trp or kan/neo promoters and the downstream TEs. (B) Sequence of Tn5-714 (highlighted in grey in A), indicating the transposon ends (red), Ptrp and kan/neo promoters (dotted arrows), the kan/neo coding region (green, underlined), and the oligo-C region synthesized in the process of Illumina library preparation. Primers used for amplification of transposon-genome junctions, and for appending the appropriate Illumina adapter sequences, are shown, with first-round primers named with the “1” prefix and second-round primers named with the “2” prefix. Asterisks indicate the location of 6-nt barcodes used to identify each experimental sample after multiplexed sequencing. Actual primer sequences are given in S6 Table. (TIF) [file pgen.1007357.s013.tif]

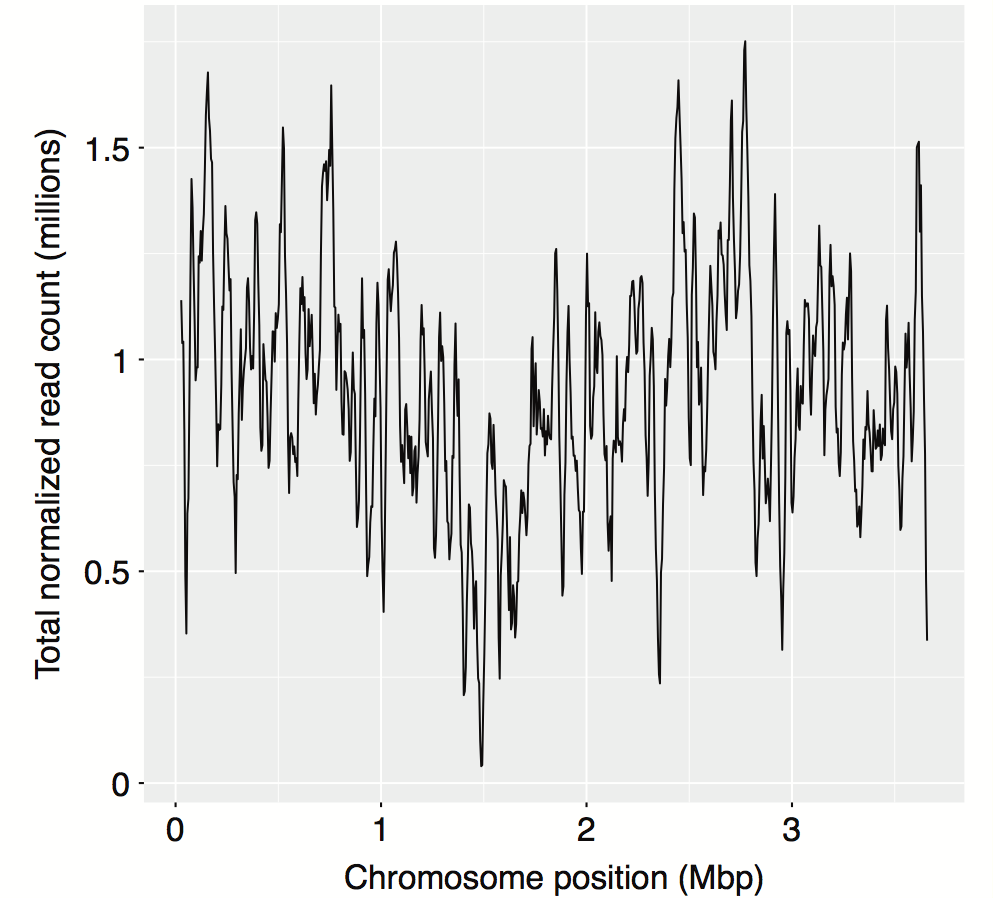

Supplement: S2 Fig — Read counts for each Tn-seq replicate were normalized by applying a multiplicative factor such that each replicate had an equal number of total reads. The reads for each replicate were the grouped into 25,000 base pair bins, with each bin shifted by 5,000 bp. The value of each bin across all eight replicates were summed, giving the final total normalized read count in each 25,000 base pair bin. The results of this analysis are provided in this figure. The X-axis represents the S. meliloti chromosome, starting at nucleotide position one in the RefSeq genome annotation. The Y-axis indicates the total number of normalized read counts in each 25,000 base pair bin. (TIF) [file pgen.1007357.s014.tif]

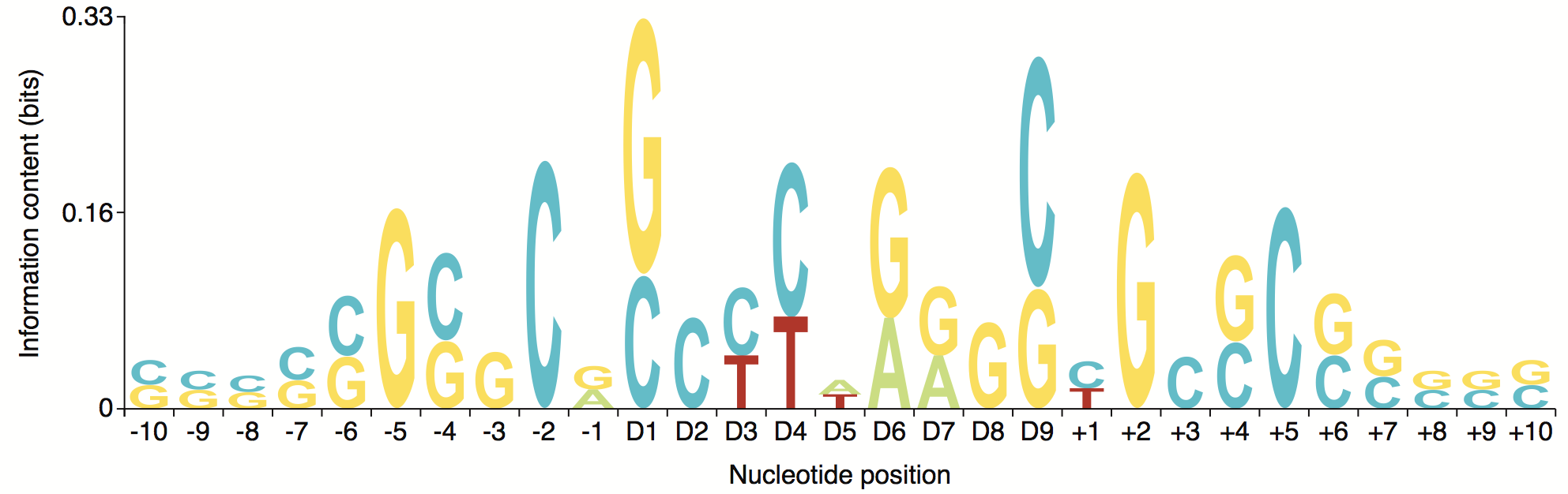

Supplement: S3 Fig — The location of each chromosomal transposon insertions in one replicate of the wild-type strain grown in rich medium was determined, and 186,102 unique positions were identified. Shown are the 10 nucleotides before the insertion site (-10 to -1), the 9 nucleotides that are part of the duplicated region (D1 to D9), and the 10 nucleotides following the insertion site (+1 to +10). The logo was generated from the hidden Markov model of the 186,102 unique insertion positions. The enrichment of G and C in the left and right ends of the logo are simply a consequence of the high GC content of the genome. (TIF) [file pgen.1007357.s015.tif]

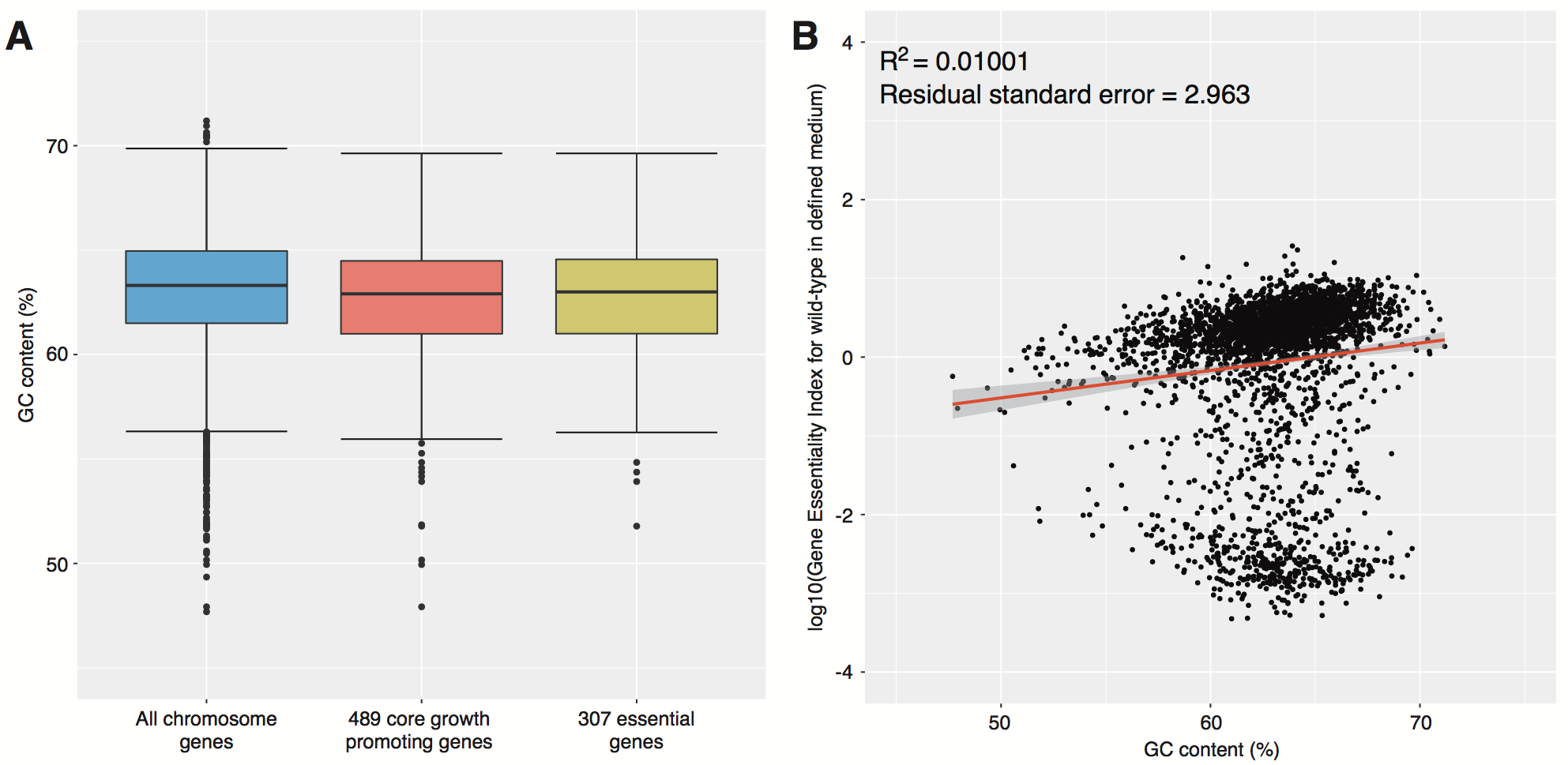

Supplement: S4 Fig — (A) These box and whisker plots summarize the distribution of the GC content of all chromosomal genes (left), the 489 core growth promoting genes (middle), and the 307 core essential genes (right). (B) A scatter plot showing the Gene Essentiality Index (GEI) scores for each gene against the GC content of the genes. The R2 value and the residual standard errors are given. (TIF) [file pgen.1007357.s016.tif]

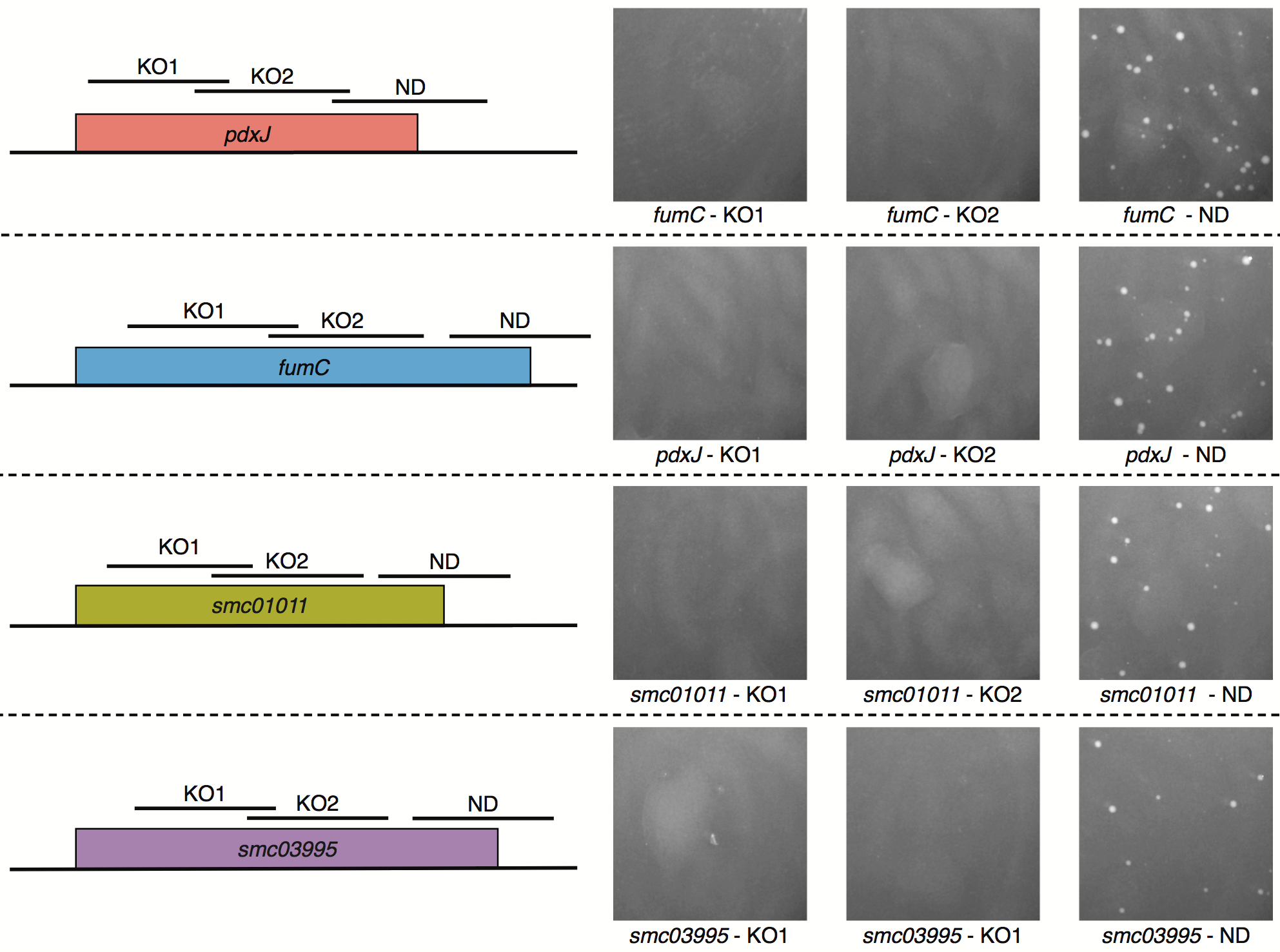

Supplement: S5 Fig — Four genes identified as essential based on the Tn-seq output were independently validated by attempting to disrupt the coding sequences through single cross-over plasmid integration. Three plasmids were designed for each gene, two that would disrupt the coding sequence (KO1 and KO2), and one that would not disrupt the coding sequence (ND) as a control. The locations of each construct are shown on the left. Each plasmid was transferred to wild-type S. meliloti via conjugation, and transconjugants selected on rich medium. Pictures of the selective plates are shown on the right. In all cases, colonies were only observed for conjugations involving the ND plasmids, validating what was observed in the Tn-seq data. (TIF) [file pgen.1007357.s017.tif]

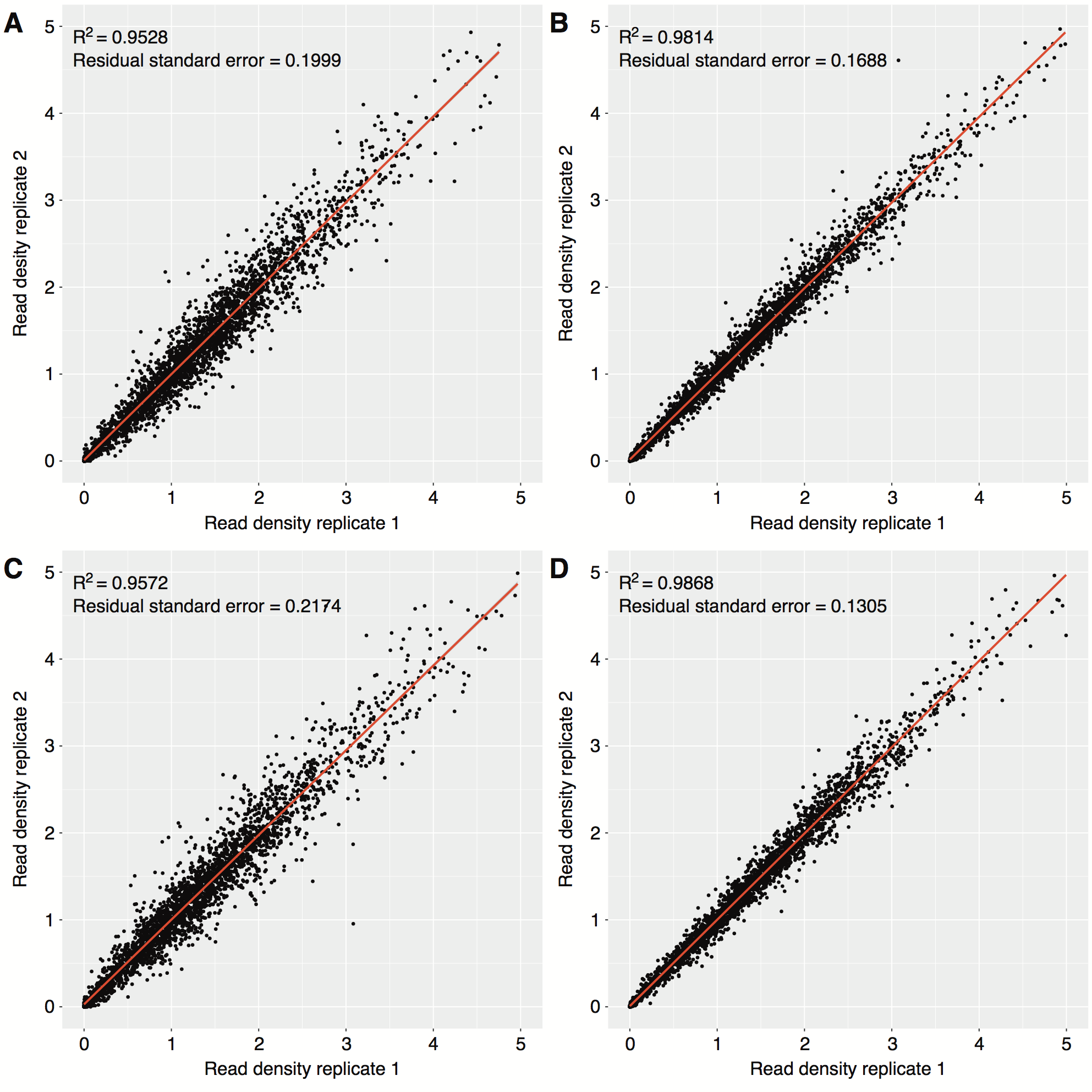

Supplement: S6 Fig — Duplicate experiments were performed for each strain in each medium. The insertion density of each gene in each experiment was calculated as the number of insertions per nucleotide. These graphs plot the insertion density of the two duplicates of each experiment. The linear regression line of the data is shown in red, and the adjusted R2 values and the residual standard errors are shown in the top left of each graph. Depending on the condition, between 9 and 34 genes had an average of more than 5 reads per nucleotide, and these genes were excluded from these graphs. (A) Wild-type grown in the rich medium, (B) ΔpSymAB grown in the rich medium, (C) wild-type grown in the defined medium, and (D) ΔpSymAB grown in the defined medium. (TIF) [file pgen.1007357.s018.tif]

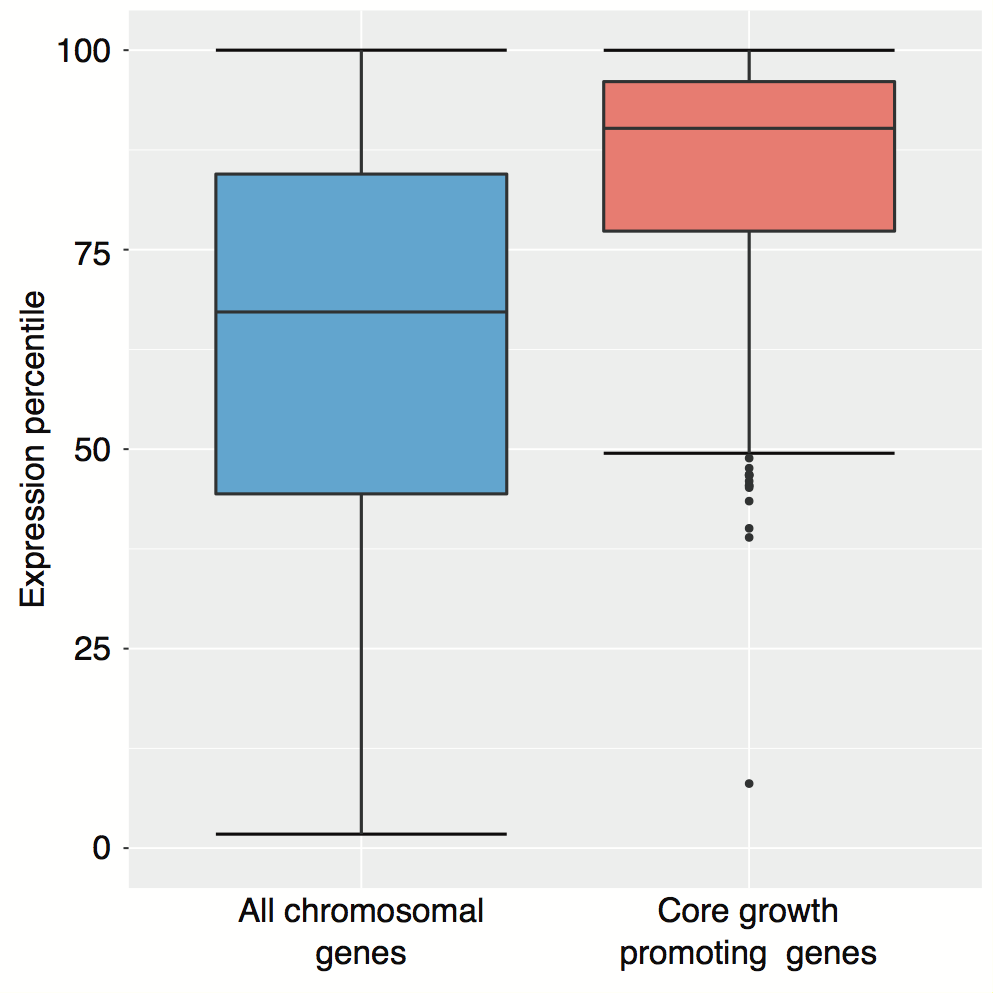

Supplement: S7 Fig — The RPKM values for all S. meliloti genes (including the chromosome, pSymB, and pSymA) when grown in a glucose minimal medium were extracted from the dataset of diCenzo et al. [94]. Genes were assigned to a percentile value, with higher expressed genes belonging to higher percentiles. These box and whisker plots summarize the distribution of expression values for all chromosomal genes (left), and for the 489 core growth promoting genes (right) as identified in this study. (TIF) [file pgen.1007357.s019.tif]

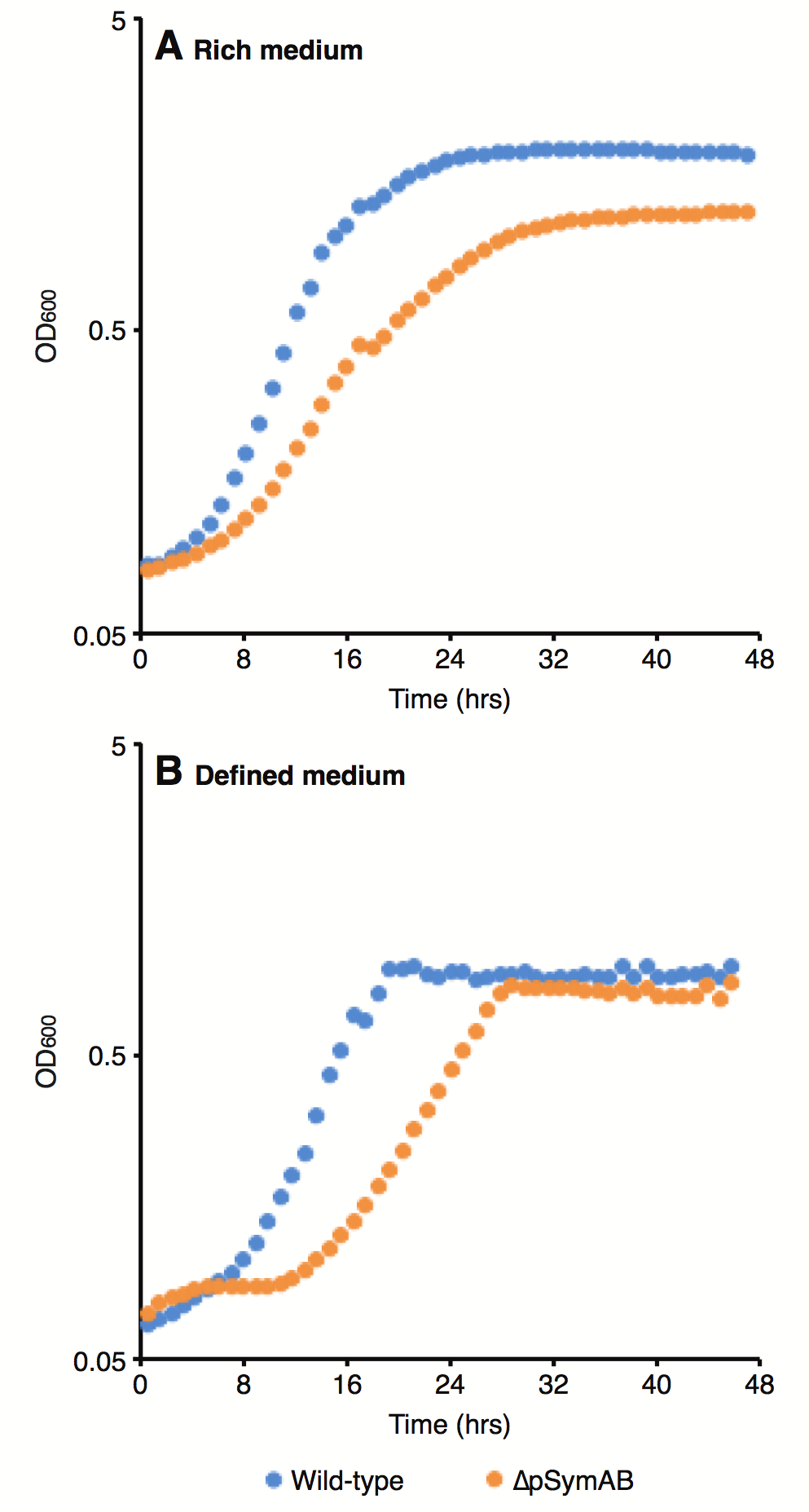

Supplement: S8 Fig — Growth profiles are shown for the wild-type (RmP3499) and ΔpSymAB (RmP3496) S. meliloti strains grown in (A) rich medium or (B) defined medium. Data points represent the average of triplicate samples, and do not have the blank value subtracted. (TIF) [file pgen.1007357.s020.tif]

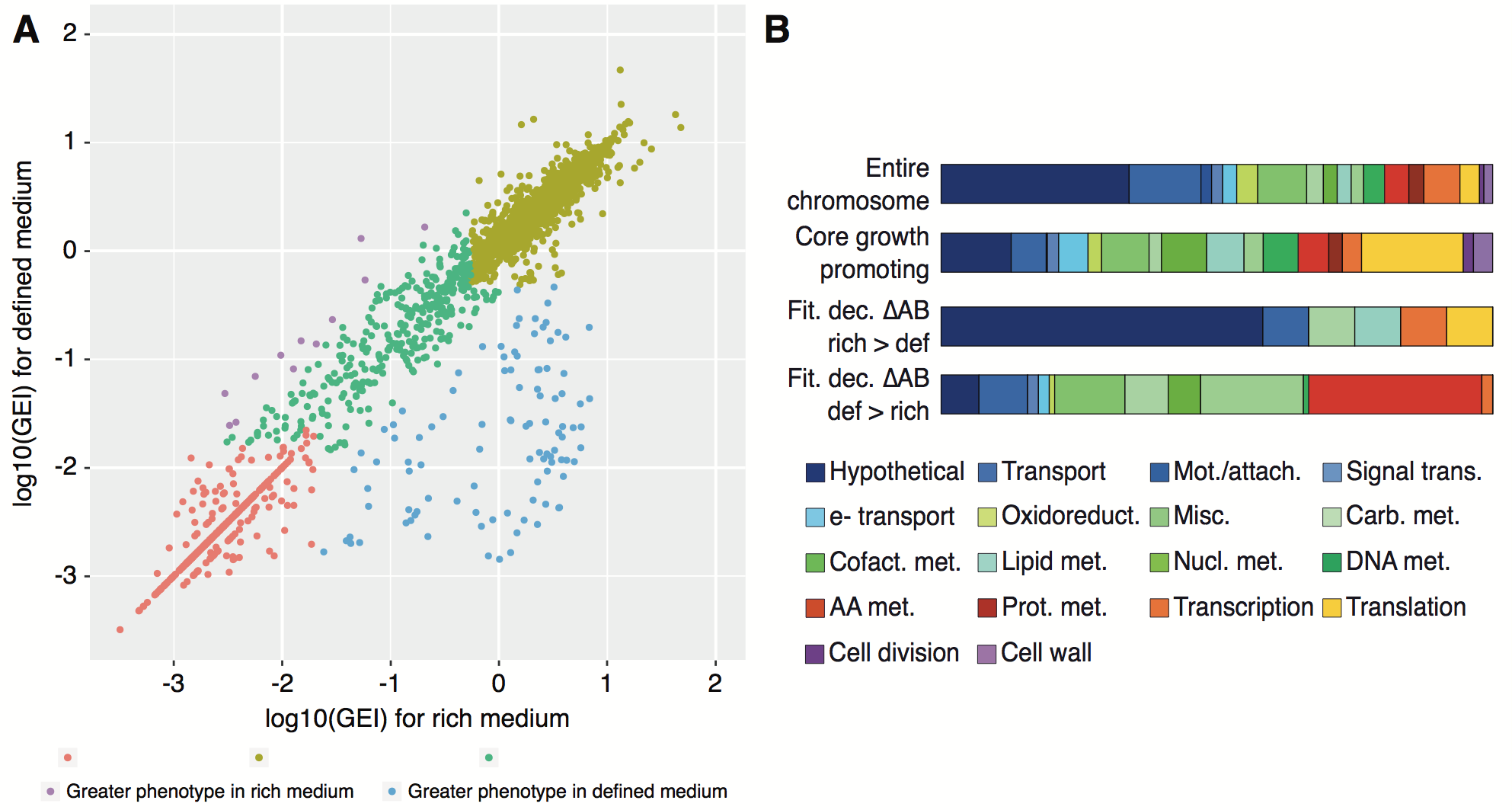

Supplement: S9 Fig — (A) A scatter plot comparing the fitness phenotypes of ΔpSymAB grown in rich medium versus ΔpSymAB grown in defined medium. (B) Functional enrichment plots for the indicated gene sets. Name abbreviations: Fit–fitness; Dec–decrease; ΔAB—ΔpSymAB; Def–defined medium; Rich–rich medium. For example, ‘Fit. dec. ΔAB def > rich' means the genes with a greater fitness decrease in ΔpSymAB grown in defined medium compared to rich medium. Legend abbreviations: AA–amino acid; Attach–attachment; Carb–carbohydrate; Cofact–cofactor; e-–electron; Met–metabolism; Misc–miscellaneous; Mot–motility; Nucl–nucleotide; Oxidoreduct–oxidoreductase activity; Prot–protein; Trans–transduction. (TIF) [file pgen.1007357.s021.tif]

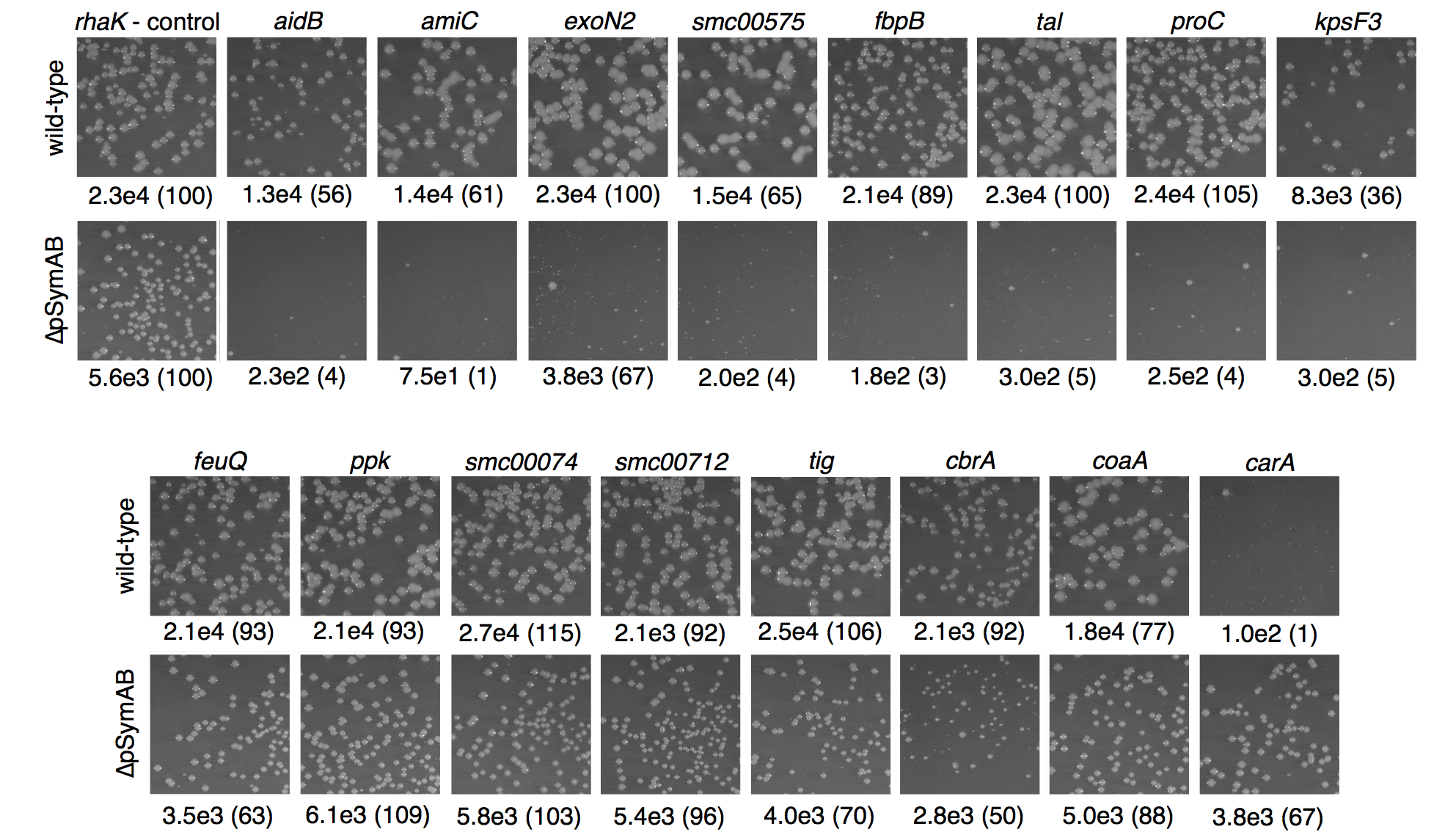

Supplement: S10 Fig — Knockout mutations, generated through single cross-over plasmid integration, of 17 genes were prepared. Numbers shown are the CFU/mL of transductions (based on both large and small colonies), and the % CFU/mL of the rhaK control is indicated. All transductions were performed at least two independent times, and numbers from a representative experiment are shown. These included one control gene (rhaK) whose mutation was expected to have no phenotype in these conditions, 14 genes identified as specifically required in the ΔpSymAB based on the Tn-seq data, and two genes (carA, coaA) identified as specifically required in the wild-type for growth on rich media based on the Tn-seq data. All mutations were originally made in the wild-type background, except for carA that was made in the ΔpSymAB background. Mutations were then moved by transduction into both the wild-type and ΔpSymAB strains, and transductants recovered on selective media. Rich medium selection plates from the transductions are shown. Plates involving the ΔpSymAB strain were incubated for two days longer than those involving the wild-type. (TIF) [file pgen.1007357.s022.tif]

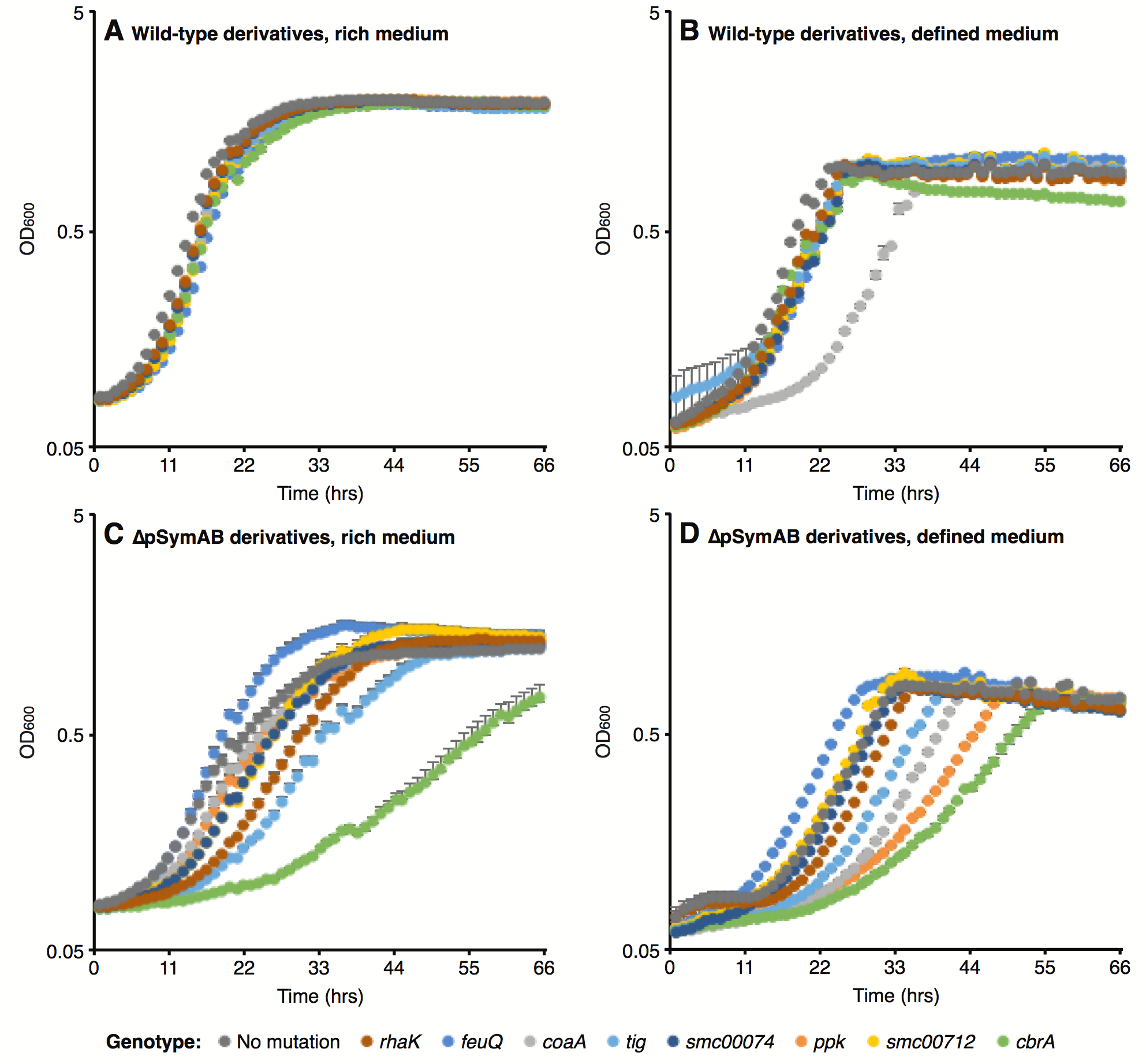

Supplement: S11 Fig — Growth curves are shown for S. meliloti strains with mutations of the seven genes determined to be specifically required in the ΔpSymAB strain based on the Tn-seq data, but that were non-essential when independently mutated based on the transduction experiment (S10 Fig). The ‘no mutation’ genotype refers to the wild-type or ΔpSymAB strains without additional mutations, and the rhaK strain is a control mutation expected to have no effect on growth. Data points represent the average of triplicate samples and do not have the blank value subtracted, while the error bars show the standard error. Growth is shown for (A) wild-type derivatives grown in rich medium, (B) wild-type derivatives grown in defined medium, (C) ΔpSymAB derivatives grown in rich medium, and (D) ΔpSymAB derivatives grown in defined medium. (TIF) [file pgen.1007357.s023.tif]

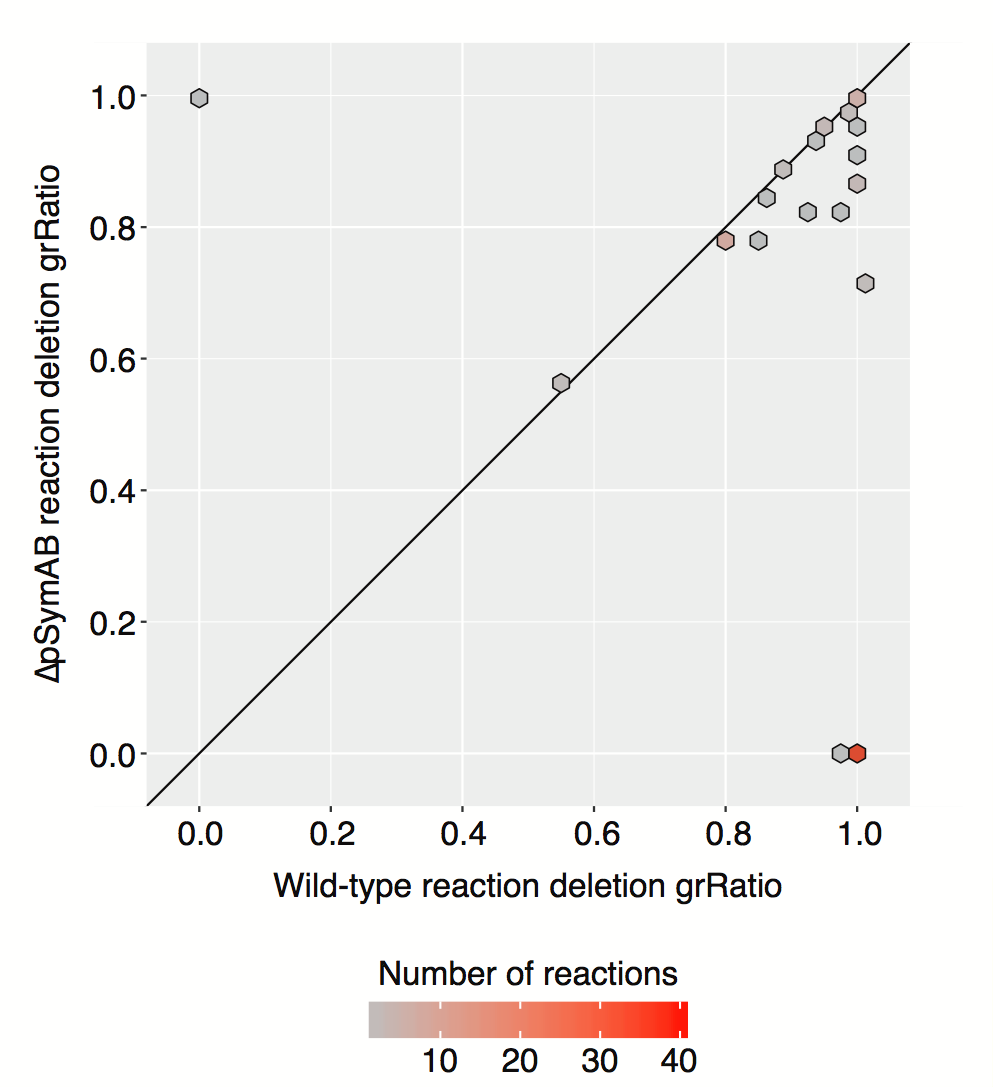

Supplement: S12 Fig — Each reaction dependent on a chromosomal gene in the S. meliloti genome-scale metabolic model was deleted in the presence and absence of pSymA/pSymB dependent reactions, and the effect on growth recorded as a grRatio value (growth rate of mutant / growth rate of non-mutant). The grRatio of the reaction deletions in the presence (X-axis) compared to the absence (Y-axis) of the pSymA/pSymB reactions was plotted, and the points binned using hexagonal binning. Reactions whose deletion had no effect in both models or whose deletion was lethal in both models are not included in this figure. The color of each hexagon is representative of the number of reactions plotted at that location, as illustrated by the density bar at the bottom of the figure. The diagonal line serves as a reference line for reactions having equal effect in both conditions. (TIF) [file pgen.1007357.s024.tif]

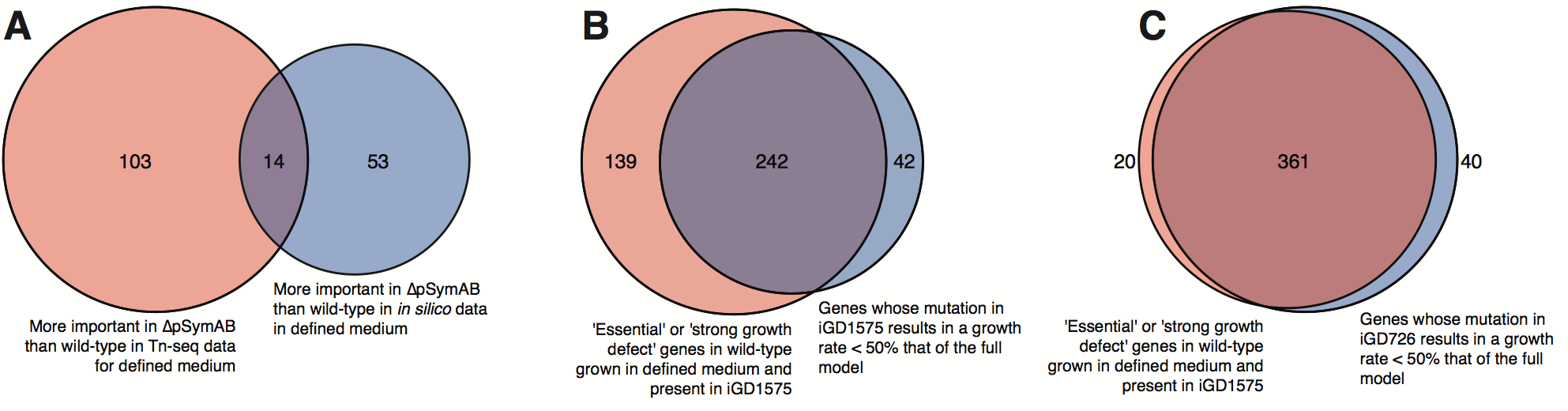

Supplement: S13 Fig — Venn Diagrams are shown to illustrate the extent of overlap between different experimental and in silico datasets. All Tn-seq data are taken for strains grown in defined medium. (A) For chromosomal genes included in iGD1575 (the original genome-scale S. meliloti metabolic model), the overlap between those observed to have a synthetic negative phenotype in the absence of pSymA and pSymB as determined with Tn-seq and metabolic modeling is shown. (B) For chromosomal genes included in iGD1575, the overlap between those observed as important to growth (group I and II genes) in the Tn-seq dataset with those predicted by metabolic modeling to result in at least a 50% decrease in growth rate when deleted in iGD1575 are shown. (C) For chromosomal genes included in iGD1575, the overlap between those observed as important for growth (group I and II genes) in the Tn-seq dataset with those predicted by metabolic modeling to result in at least a 50% decrease in growth rate when deleted in iGD726 (the core model) is shown. (TIF) [file pgen.1007357.s025.tif]

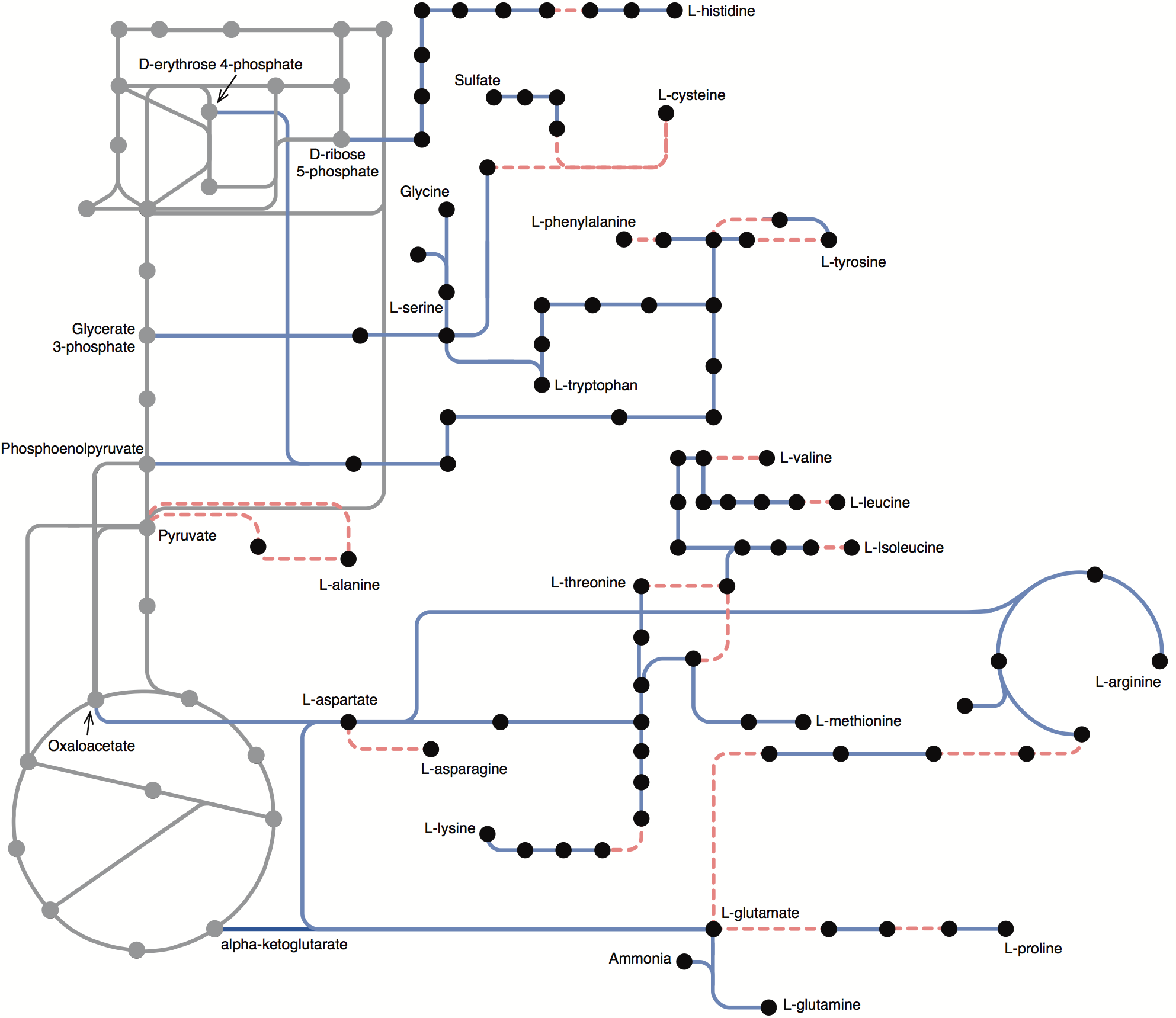

Supplement: S14 Fig — A summary schematic of the amino acid biosynthetic pathways of S. meliloti are shown, based on the iGD726 core metabolic reconstruction. Central carbon metabolism is displayed in grey. The 20 amino acids and important precursor compounds are labeled. Genes associated with the reactions in solid blue lines were supported by the Tn-seq data of this study, while the genes associated with the reactions in dashed red lines were not supported by the Tn-seq data of this study. (TIF) [file pgen.1007357.s026.tif]

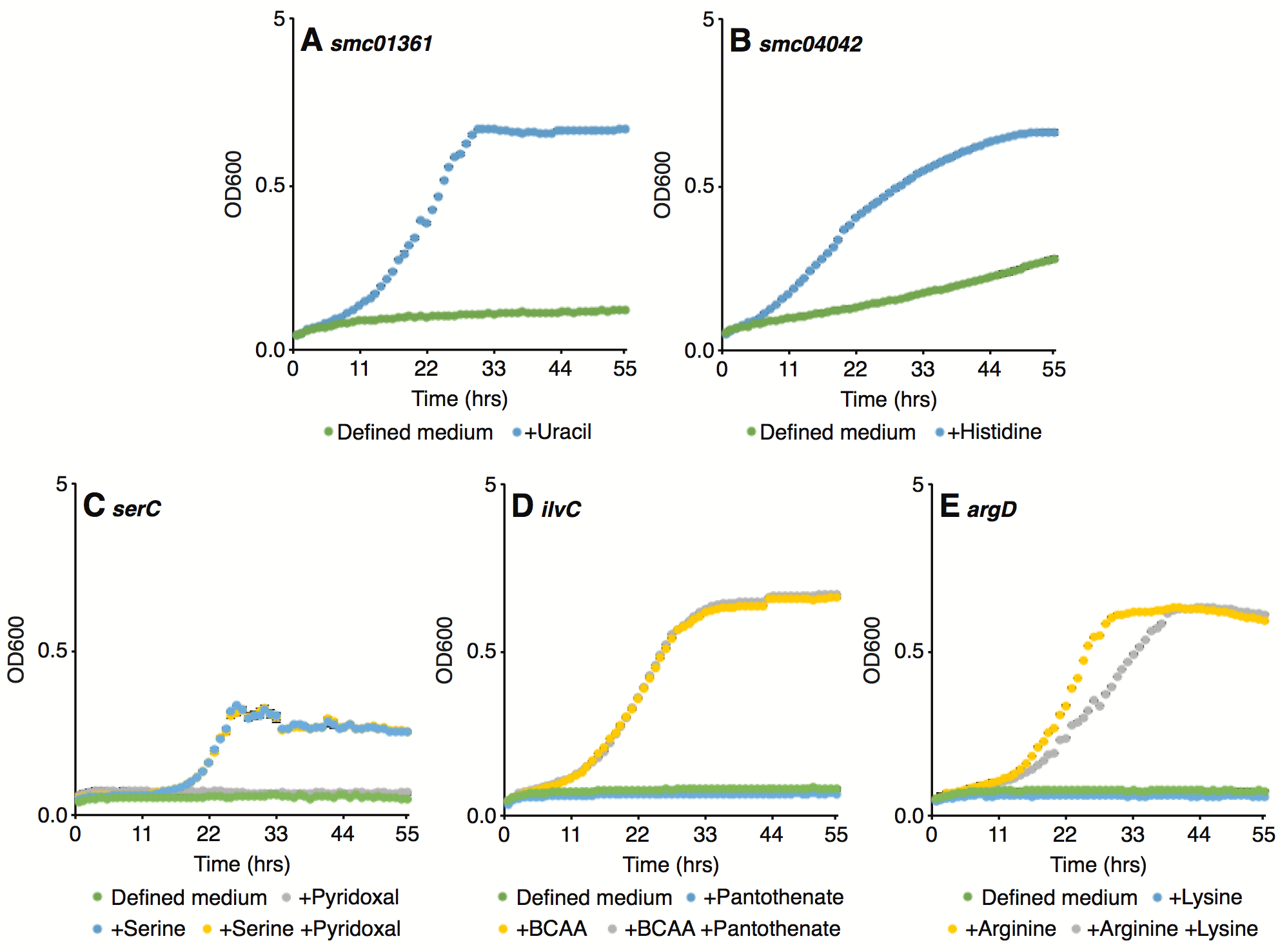

Supplement: S15 Fig — Growth curves of five S. meliloti mutants are shown in minimal medium supplemented with the indicated nutrients. ‘Defined medium’ indicates the medium was not supplemented with any nutrients aside from the base defined medium composition, while other designations indicate which nutrient(s) were added to the base defined medium. BCAA stands for ‘branched chain amino acids’. Data points represent the average of triplicate samples and do not have the blank value subtracted, while the error bars show the standard error. (A-D) The mutations are in the wild-type (RmP3499) background. (E) The mutation is in the ΔpSymAB (RmP3496) background as the argD mutation does not result in auxotrophy in the wild-type background. (TIF) [file pgen.1007357.s027.tif]

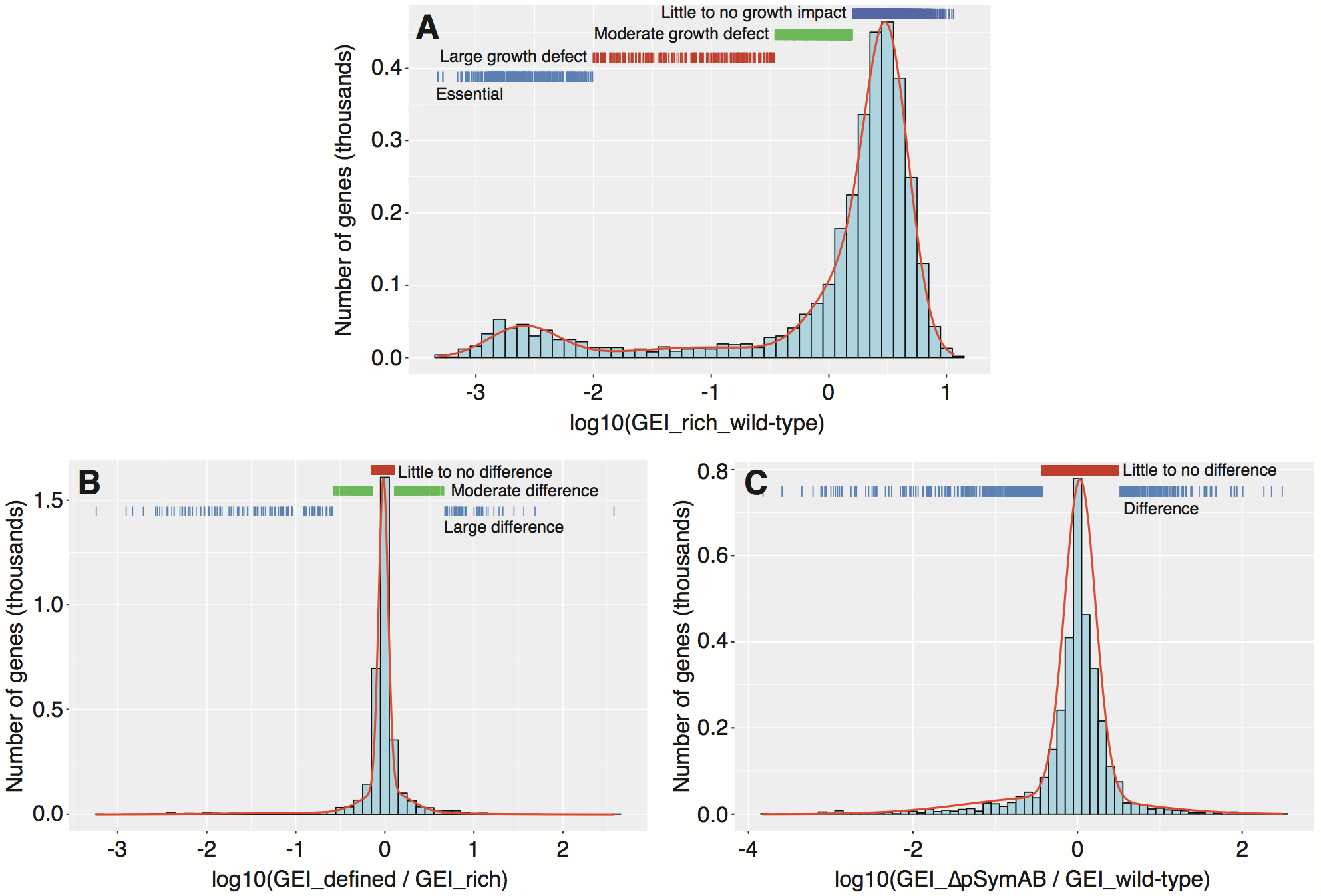

Supplement: S16 Fig — Sample input data and clustering as determined using Mclust is shown. In each figure, the bars represent a histogram that shows the complete distribution of the data. The smooth red line is the estimated density as calculated by Mclust based on the data distribution. The colored lines along the top represent the clustering as performed by Mclust; individual lines represent individual genes, with the color indicating the different clusters (names of the clusters are indicated). (A) Data and clustering for the Gene Essentiality Index (GEI) scores for the wild-type grown in the rich medium. (B) Data and clustering of the fold changes in the GEI scores of genes in the wild-type when grown in defined medium relative to rich medium. (C) Data and clustering of the fold changes in the GEI scores of genes in the ΔpSymAB strain relative to the wild-type strain when grown in defined medium. (TIF) [file pgen.1007357.s028.tif]
